# Manganese-Templated Nontrivial Structures for MRI and Therapy

**DOI:** 10.1021/jacs.5c19016

**Published:** 2026-04-01

**Authors:** Farah Benyettou, Thirumurugan Prakasam, Mostafa Khair, Osama Abdullah, Matteo Lusi, Haidee Paterson, Maryam Alkaabi, Sneha Thomas, Rainer Straubinger, Nosayba Al Damook, Maylis Boitet, Mamoun Abelbaki, Judalyn Del Monte, Diana Yu, Rick E. Heinz, Sheri L. Holmen, Edward Hsu, Carlos Platas-Iglesias, Gennaro Esposito, Ali Trabolsi

**Affiliations:** † Chemistry Program, New York University Abu Dhabi (NYUAD), Abu Dhabi 129188, United Arab Emirates; ‡ Core Technology Platforms, New York University Abu Dhabi (NYUAD), Abu Dhabi 129188, United Arab Emirates; § Department of Chemical Science & Bernal Institute, University of Limerick, Limerick V94 T9PX, Republic of Ireland; ∥ PHRC, New York University Abu Dhabi (NYUAD), Abu Dhabi 129188, United Arab Emirates; ⊥ Department of Surgery, University of Utah Health Sciences Center, Salt Lake City, Utah 84112, United States; # Huntsman Cancer Institute, University of Utah Health Sciences Center, Salt Lake City, Utah 84112, United States; ∇ Department of Biomedical Engineering, University of Utah, Salt Lake City, Utah 84112, United States; ○ Centro Interdisciplinar de Química e Bioloxía (CICA) and Departamento de Química, Facultade de Ciencias, Universidade da Coruña, A Coruña Galicia 15071, Spain

## Abstract

Manganese (Mn)-based metal–organic architectures offer a unique avenue for integrating magnetic resonance imaging (MRI) and cancer therapy within a single molecular platform. We report three topologically distinct Mn-templated structures—Mn-[2]­Catenate (Mn-[2]­C), Mn-Trefoil Knot (Mn-TK), and Mn-Borromean Rings (Mn-BR)—that combine high relaxivity with tumor-selective cytotoxicity. The design leverages their geometrical complexity and electropositive, pH-labile coordination framework to ensure kinetic stability and lipophilicity at physiological pH while enabling Mn^2+^ release in the acidic tumor microenvironment. Among the three, Mn-BR and Mn-TK exhibit superior longitudinal relaxivities (*r*_1_ = 10.1 and 6.8 mM^–1^.s^–1^ at 3 T) and produce bright *T*_1_-weighted contrast exceeding that of Gd-DTPA and Mn-DPDP. In vitro, they show high cancer selectivity and potency in glioblastoma (U251-MG) cells, with IC_50_ values of 3.0 ± 0.9 μM (Mn-BR) and 5.6 ± 1.9 μM (Mn-TK), outperforming cisplatin (12.7 ± 2.5 μM) while sparing normal cells (SI > 3.9 for Mn-TK; SI > 9.4 for Mn-BR). Mechanistically, their uptake proceeds via energy-dependent endocytosis—caveolae-mediated for Mn-TK and clathrin/macropinocytosis-driven for Mn-BR—culminating in lysosomal acidification, pH-triggered disassembly, Mn^2+^ release, ROS accumulation, and caspase-dependent apoptosis. In vivo, Mn-TK and Mn-BR achieve tumor-specific accumulation, strong MRI contrast, and pronounced growth inhibition in subcutaneous glioblastoma models, while Mn-[2]C shows minimal selectivity and higher systemic toxicity. Importantly, in a spontaneous orthotopic glioblastoma model, both Mn-TK and Mn-BR provided robust BBB permeability and persistent, tumor-specific MRI enhancement, confirming their potential for precise MRI-guided tumor visualization. This research marks a major leap forward in medical nanotechnology, unveiling a new class of metal–organic structures that seamlessly integrates imaging and therapy. By unlocking their full potential, these structures promise to revolutionize MRI diagnostics, precision medicine, and next-generation cancer treatments, paving the way for unparalleled clinical outcomes.

## Graphical abstract

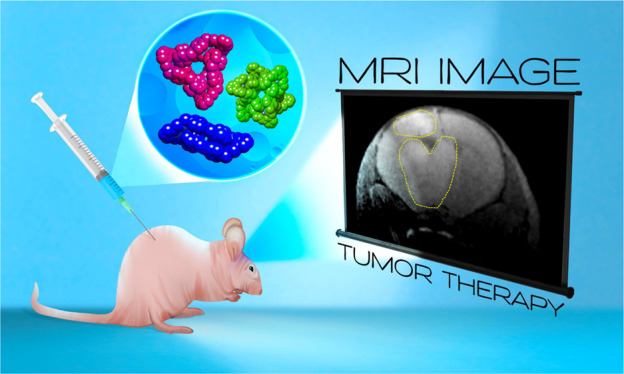

## 1 Introduction

Magnetic resonance imaging (MRI) remains a cornerstone of modern diagnostics due to its noninvasive nature and high spatial resolution. However, the performance of MRI often depends on contrast agents that enhance tissue differentiation by altering the relaxation times of surrounding water protons.^1^ For decades, gadolinium­(Gd)-based contrast agents (GBCAs) have dominated clinical use.^2^ Despite their effectiveness, GBCAs are associated with critical safety limitations: free Gd^3+^ ions are highly toxic, and even chelated forms have been linked to nephrogenic systemic fibrosis (NSF) in patients with impaired renal function.^3^ Moreover, Gd deposition in brain and bone tissues, detected even after a single administration, raises concerns about long-term bioaccumulation and neurological risks.^4^

These issues are particularly critical in brain imaging. The blood–brain barrier (BBB), while essential for neuroprotection, severely limits the delivery of contrast agents to brain tumors.^5^ In highly infiltrative glioblastoma (GBM), where tumor margins are poorly defined, this limitation hampers accurate tumor visualization and surgical planning.^5^ Conventional GBCAs accumulate predominantly in regions of disrupted BBB, failing to capture the full extent of tumor infiltration. To overcome these challenges, there is increasing interest in developing safer, more effective MRI agents that can traverse the BBB, selectively accumulate in tumors, and reduce systemic toxicity.^5^

In this context, manganese (Mn^2+^)-based contrast agents have emerged as promising Gd-free alternatives.^6–9^ Mn^2+^ is a paramagnetic ion with five unpaired electrons, making it well suited for *T*_1_-weighted MRI. As a naturally occurring trace element in the body,^10^ when appropriately chelated, Mn^2+^ offers a favorable toxicity profile than Gd^3+^. However, earlier Mn-based agents such as Mn-DPDP (Teslascan)^11^ and MnCl_2_ (LumenHance)^12^ demonstrated limited clinical utility due to their low kinetic stability, insufficient targeting capacity, and the risk of free Mn^2+^ release, which can cause neurotoxic effects resembling manganism.^13–15^ These drawbacks underscore the need for next-generation Mn platforms with enhanced stability and tumor specificity.

Here, we present three manganese-templated nontrivial topological structures (Figure 1a), including [2]­Catenate (Mn-[2]­C), Trefoil Knot (Mn-TK), and Borromean Rings (Mn-BR), as dual-function MRI contrast agents and therapeutics. These topologically complex metal–organic architectures exhibit high thermodynamic stability at physiological conditions, resistance to transmetalation, lipophilicity, and favorable in vivo clearance profiles.^16–20^ Unlike conventional chelates, these compounds are engineered to offer a labile inner-sphere water coordination site, essential for high relaxivity and signal intensity in *T*_1_-weighted MRI.^21^ Moreover, their polynuclear Mn cores and electropositive, lipophilic surfaces promote interactions with tumor cell membranes, enhancing cellular accumulation and internalization, while their acid-labile topological structure enables pH-triggered disassembly and Mn^2+^ release in the tumor microenvironment, supporting selective cytotoxicity and imaging within the acidic tumor microenvironment.

**Figure 1:**
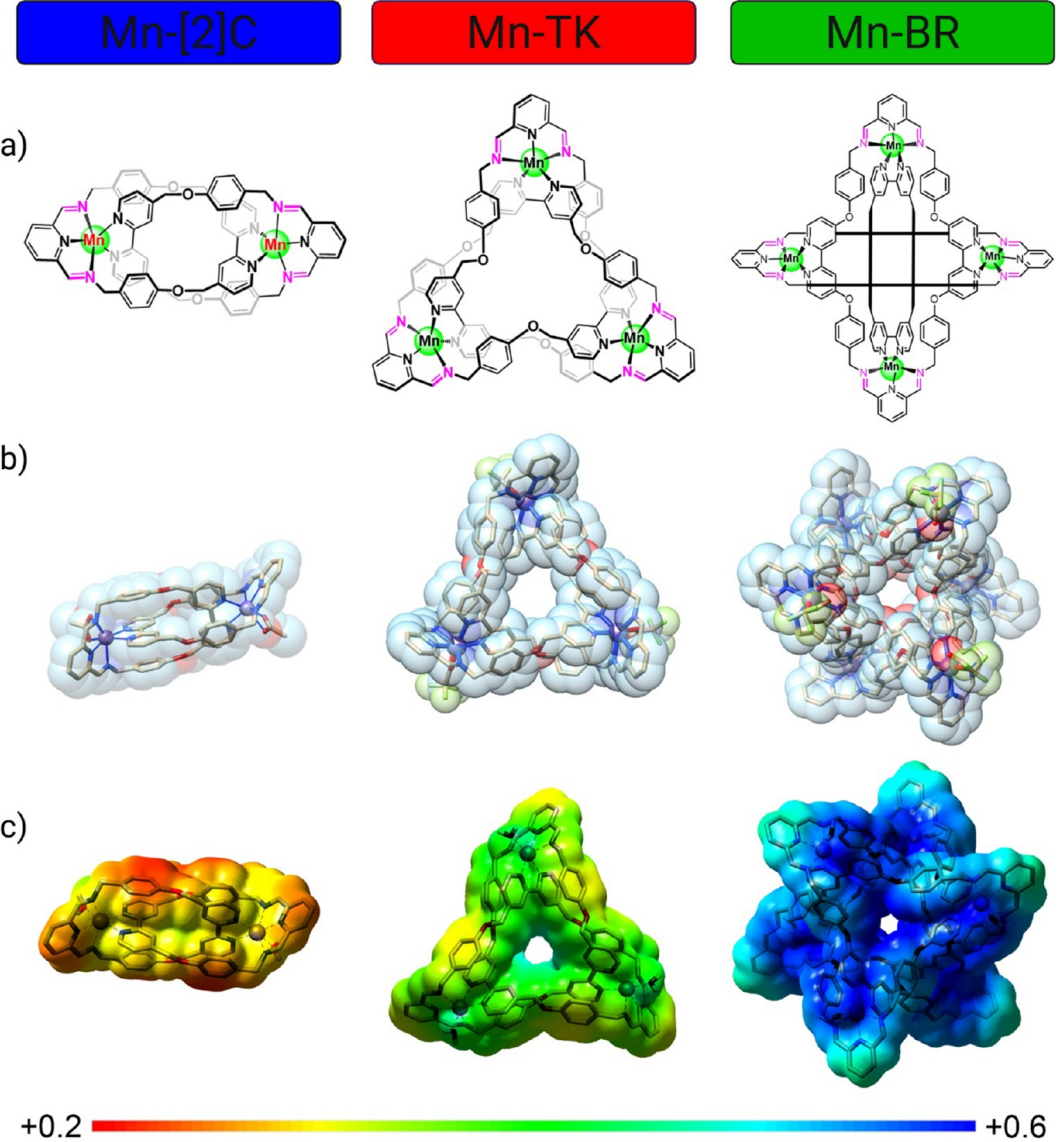
Topology, structure, and electrostatic profiles of manganese-organic nontrivial structures. (a) Topological schematics of the three Mn­(II) assemblies—Mn-[2]­Catenate (Mn-[2]­C), Mn-Trefoil Knot (Mn-TK), and Mn-Borromean Rings (Mn-BR)—with Mn centers indicated in green. (b) X-ray crystal structures of the three manganese-based nontrivial structures: Mn-[2]­Catenate (Mn-[2]­C), Mn-Trefoil Knot (Mn-TK), and Mn-Borromean Rings (Mn-BR). (c) Corresponding molecular electrostatic potential maps, color-coded in atomic units (a. u.) or Hartrees (1 hartree = 627.5095 kcal.mol^–1^), for Mn-[2]­C, Mn-TK, and Mn-BR, to indicate potential ranges from low (+0.2 a.u.; red) to high (+0.6 a.u.; blue), highlighting regions of potentially different reactivity within each structure. These images visually represent the complexity of the structural and electrostatic properties of metal–organic structures, which are important for targeted therapeutic applications and MRI contrast enhancement.

These properties align with the principles of theranostics, where a single agent integrates diagnostic and therapeutic functions.^22^ Beyond enhancing contrast, Mn-templated nontrivial structures undergo pH-responsive disassembly and exhibit selective cytotoxicity against cancer cells, offering targeted therapeutic action. Prior studies from our group demonstrated that Metal-templated Trefoil Knots induce mitochondrial apoptosis via reactive oxygen species (ROS) in drug-resistant cancer cells, sparing healthy tissue.^18^ In this work, we demonstrate that these Mn-based structures (i) remain stable at physiological pH, (ii) exhibit high *T*_1_ relaxivity that exceeds clinically evaluated Mn agents such as Mn-DPDP, Mn-NOTA, and Mn-PyC3A,^23^ and (iii) enable tumor-specific Mn^2+^ release, driving selective anticancer activity both in vitro and in vivo. MRI-guided studies in glioblastoma models confirm enhanced tumor localization, strong contrast enhancement, and therapeutic efficacy, establishing Mn-TK and Mn-BR as next-generation theranostic platforms. Although Mn-[2]C demonstrated activity in vitro, it failed to show selectivity toward cancer cells and produced significant toxicity in preliminary animal studies. This was likely due to diffuse, nonspecific uptake, low tumor retention, and off-target accumulation, resulting in poor tolerability at imaging doses. These observations highlight the critical role of molecular topology in achieving selective targeting and acceptable safety profiles. This study offers a framework for integrating topological coordination chemistry into precision diagnostics and targeted cancer therapy.

## 2 Results and Discussion

### 2.1 Synthesis, Characterization, and Physiological Stability of Manganese-Based Metal–Organic Nontrivial Structures

#### 2.1.1 Manganese Templated Nontrivial Structures

The three structures, Mn-[2]­Catenate (Mn-[2]­C), Mn-Trefoil Knot (Mn-TK), and Mn-Borromean Rings (Mn-BR), were synthesized using an optimized direct one-pot synthesis method (Figure 1a, Scheme S1).^16–20^ This process involves the coordination-driven assembly of two chelating organic ligands, diamino pyridine (DAB) and 2,6-diformylpyridine (DFP), with Mn^2+^ ions in equimolar ratios (Scheme S1).^16–20^ The successful formation of the metal-templated structures was confirmed by high-resolution mass spectrometry (HR-MS, Figure S1) and single-crystal X-ray diffraction (Figure 1b).

#### 2.1.2 Stability at Neutral and Acidic pH Levels

The chemical stability of the three structures was first assessed under physiological conditions (pH 7.4 in HEPES buffer). All three constructs remained structurally intact over 7 days, with no detectable hydrolysis of the imine bonds, as confirmed by mass spectrometry (Figure S1) and UV–vis spectroscopy (Figure S2). However, at pH 5.4, these structures disintegrate due to the hydrolysis of the imine bonds, indicating their susceptibility to acidic environments (Figures S1 and S3). Among them, Mn-[2]C exhibits the greatest resistance to acid-mediated degradation, maintaining its structural integrity for up to 2 weeks, likely due to its more compact structure.^24^ This pH-dependent instability suggests that Mn-TK and Mn-BR are selectively labile in the acidic tumor microenvironment, enabling the release of their active components. Such environmentally triggered disassembly supports their utility for targeted cancer therapy, enabling the controlled release of therapeutic metals at the tumor site while preserving systemic stability under physiological conditions.

#### 2.1.3 Kinetic Inertness and Transmetalation Studies

The kinetic inertness of metal complexes, often more critical than their thermodynamic stability, plays a decisive role in determining their in vivo performance and safety.^25,26^ To assess the stability of the three Mn structures, we investigated their susceptibility to transmetalation with zinc ions (Zn^2+^),^25,26^ one of the most abundant trace metals in the human body. Transmetalation by endogenous Zn^2+^ poses a significant risk, as it can displace Mn^2+^ from its coordination environment, potentially compromising contrast efficacy and safety (Figure S4). Mass spectrometry analyses revealed that all three Mn architectures, Mn-[2]­C, Mn-TK, and Mn-BR, exhibited strong resistance to Zn^2+^-induced transmetalation, with no detectable dissociation over a 24-h incubation period. In stark contrast, mangafodipir (Mn-DPDP), a previously approved Mn-based liver imaging agent, readily releases free Mn^2+^ upon administration, contributing to systemic toxicity and limiting its clinical utility.^23^ The enhanced kinetic inertness of the Mn-templated topological structures is attributed to their rigid, nontrivial coordination frameworks, which confer both steric shielding and multivalent metal–ligand interactions. This exceptional stability underscores their promise as safer, more durable MRI agents for translational biomedical applications.

#### 2.1.4 Lipophilicity

The lipophilicity of Mn-[2]­C, Mn-TK, and Mn-BR was experimentally determined using the standard *n*-octanol/water shake-flask method followed by UV–Vis quantification. The resulting logP values were −0.795 for Mn-[2]­C, −0.463 for Mn-TK, and −0.491 for Mn-BR (Figure S5). These results indicate that Mn-TK and Mn-BR exhibit slightly higher lipophilicity than Mn-[2]­C, though all three structures remain moderately hydrophilic compared to many reported Mn-based MRI contrast agents (Table S1). Clinical agents such as Mn-DPDP or Mn-EDTA derivatives generally display logP values below −2,^27^ whereas structural modifications with aromatic or alkyl substituents, as in Mn-PyC3A-3-OBn^28^ or Mn-BnO-TyrEDTA,^29^ can shift logP into the positive range. The values measured here place Mn-[2]­C, Mn-TK, and Mn-BR in an intermediate regime, where adequate aqueous solubility is preserved while allowing moderate affinity for nonpolar phases. This physicochemical balance is favorable for formulation stability and the ability to engage transiently with lipid bilayers, which may promote passive diffusion or adsorptive-mediated membrane crossing, including penetration of the blood–brain and blood–tumor barriers.

### 2.2 ^17^O NMR Study

For Mn^2+^ structures to function effectively as *T*_1_-weighted MRI contrast agents (CAs), the paramagnetic center must retain at least one inner-sphere water coordination site.^30^ To assess this requirement, we investigated the hydration number and water exchange dynamics of the Mn-templated structures using temperature-dependent ^17^O NMR spectroscopy. Transverse relaxation rates (1/*T*_2_) of ^17^O-enriched water were measured at 11.7 T over a temperature range of 1–42 °C to evaluate the structural integrity and hydration properties of Mn-BR, Mn-TK, and Mn-[2]­C. From these measurements, reduced transverse relaxation rates (1/*T*_2*r*_) were calculated by normalizing 1/*T*_2_ values to the mole fraction of coordinated water.^31^ Calculations assumed each Mn^2+^ ion coordinates with one water molecule, based on single-crystal X-ray diffraction data, in which a trifluoroacetate (TFA) anion occupies a coordination site. In aqueous solution, TFA is expected to be displaced by water, enabling effective hydration. Mn-BR, Mn-TK, and Mn-[2]­C exhibited comparable *T*_*2r*_ values, indicating similar water exchange dynamics across the three Mn-structures (Figure S6). The observed increase in ln­(1/*T*_2r_) with temperature is characteristic of the slow exchange regime. Kinetic parameters extracted by least-squares fitting of the data using the standard Swift-Connick equations,^32,33^ are summarized in Table 1.

**Table 1:** Mn-Coordinated Water Exchange Rate (*k*_ex_^298^), Activation Energy (Δ*H* ^‡^), and Scalar Hyperfine Coupling Constant (A_O_/ℏ) of Each of the Metal-Organic Structures, Calculated from the Temperature-Dependent Reduced ^17^O NMR Transverse Relaxation Rates Using Swift-Connick Equations^28,29^

	*k*_ex_^298^ (×10^6^ s^–1^)	Δ*H* ^‡^ (kJ mol^–1^)	*A*_O_/ℏ (×10^6^ rad s^–1^)
Mn-2[C]	50.1 ± 0.9	28.7 ± 0.5	–71.4 ± 1.0
Mn-TK	47.8 ± 0.8	35.7 ± 0.5	–69.7 ± 0.6
Mn-BR	41.3 ± 0.5	28.8 ± 0.5	–55.0 ± 0.9

The water exchange rates (*k*_ex_) determined for the Mn-structures are comparable to those reported for the hexaaqua-ion [Mn­(H_2_O)_6_]^2+^ (*k*_ex_^298^ = 28.2 × 10^6^s^–1^)^34^ and other positively charged Mn^2+^ complexes.^35^ Analysis of the *T*_*2r*_ data confirms that contributions from the electron spin relaxation time to the overall correlation time are negligible, allowing reliable determination of *k*_ex_ from ^17^O NMR data.^36^

In Mn^2+^ structures, transverse relaxation rates are primarily governed by the scalar mechanism, which depends on the scalar hyperfine coupling constant *A*_O_/ℏ. Data fitting provided slightly different *A*_O_/ℏ values across the three structures, with Mn-BR exhibiting a somewhat lower hyperfine coupling constant relative to Mn[2]C and Mn-TK. Nonetheless, all values fall in the upper range typically observed for Mn^2+^ systems (−28 s^–1^ × 10^6^ to −75 × 10^6^ rad.s^–1^),^31,37,38^ and are consistent with a single water molecule coordinated to the Mn^2+^ center.

Further validation of the Swift-Connick model fitting parameters was performed by determining the number of metal-coordinated water molecules represented by the *q* value, as described by Gale et al.,^31^ and detailed in the SI section 4. Briefly, after recalibration with MnCl_2_, to establish the limiting normalization constant for transverse relaxivity maxima and account for pH and concentration conditions, *q* values deviating from unity were observed for Mn-[2]C and Mn-TK. These deviations are attributed to variations in the hyperfine coupling constants and exchange rates observed in these systems relative to [Mn­(H_2_O)_6_]^2+^ reference complex. When the relaxivity data were fitted using the experimentally determined hyperfine couplings and exchange rates for each compound (Table 1; Figure S6), the best-fit inner-sphere hydration numbers were *q* ≈ 1.0 ± 0.1 for all three complexes (95% Confidence Interval = 0.9–1.1), confirming the presence of a single, coordinated water molecule. However, if the same data set is refitted using the fixed reference parameters of [Mn­(H_2_O)_6_]^2+^ as reported by Gale, Zhu, and Caravan, the apparent hydration numbers increase to *q*_app_ = 1.6 for Mn-[2]C and *q*_app_ = 1.9 for Mn-TK, while Mn-BR remains ∼1.0. This shift reflects parameter coupling in the Swift–Connick formalism, where enforcing reference values that differ from experimentally measured hyperfine and exchange constants artificially elevates *q* to reproduce the observed relaxivity behavior.

### 2.3 Relaxivity Studies and MRI Contrast Optimization

To evaluate the efficacy of the Mn-organic structures as MRI contrast agents, relaxivity measurements were performed using a 3T clinical MRI scanner. Manganese-based agents enhance MRI imaging by shortening the *T*_1_ (spin–lattice) relaxation time of water protons, thereby improving image quality.^39–41^ The performance of these agents is quantified by their *T*_1_ relaxivity (*r*_1_), which measures the increase in water relaxation rate per unit manganese concentration, expressed in mM^–1^.s^–1^. Relaxivity values (*r*_1_) were obtained from the linear correlation between the relaxation rate (1/*T*_1_, s^–1^) and the total concentration of Mn^2+^ in solution (SI section 5). The concentration of Mn^2+^ in each sample was determined by inductively coupled plasma mass spectrometry (ICP-MS). All measurements were conducted in HEPES buffer using the saturation-recovery spin–echo sequence on the 3T scanner. In addition to the three Mn-based nontrivial structures, MnCl_2_ was measured as a reference monomeric Mn^2+^ salt and representative of conventional contrast agents. This systematic evaluation enables a direct comparison of the three Mn structures in terms of their potential for clinical MRI applications. The resulting *r*_1_ values are summarized in Table 2.

**Table 2:** Comparative Relaxivity Profiles of Mn-Based Structures under Different pH Conditions^a^

	*r*_1_ (Mn mM^–1^.s^–1^)			*r*_2_ (Mn mM^–1^.s^–1^)	*r*_2_ */r*_1_
	pH 7.4	pH 7.4 + BSA	pH 5.4		
MnCl_2_	5.8 ± 0.04	9.7 ± 0.13	3.47 ± 0.20	64.1 ± 4.0	11.05
Mn-[2]C	5.4 ± 0.25	7.4 ± 0.20 ****	6.3 ± 0.20 ***	35.78 ± 1.3	6.62
Mn-TK	6.8 ± 0.18	8.9 ± 0.18 ****	4.6 ± 0.25 ****	59.78 ± 5.2	8.79
Mn-BR	10.1 ± 0.30	11.8 ± 0.21 ****	3.6 ± 0.30 ****	55.43 ± 1.2	5.48

a This table summarizes the longitudinal (*r*_1_) and transverse (*r*_2_) relaxivities of Mn-based structures (MnCl_2_, Mn-[2]­C, Mn-TK, and Mn-BR) at 3 T, along with the corresponding r_2_/r_1_ ratios. Measurements were performed in HEPES buffer (100 mM) under three conditions: physiological pH 7.4, pH 7.4 supplemented with bovine serum albumin (BSA), and acidic pH 5.4. These data highlight the pH- and protein-responsive behavior of the agents, relevant to tumor microenvironments and blood circulation. All measurements were conducted at 20 °C using a spin-echo sequence (TE = 11 ms, TR = 150–8000 ms). Data are presented as mean ± SEM from triplicate measurements. Statistical significance levels, comparing each condition against pH 7.4, are denoted as follows: *p < 0.05, **p < 0.01, ***p < 0.001, and ****p < 0.0001.

At physiological pH (7.4), the longitudinal relaxivities (*r*_1_) for Mn-[2]­C, Mn-TK, and Mn-BR, were 5.4, 6.8, and 10.1 mM^–1^.s^–1^, respectively, compared to 5.8 mM^–1^.s^–1^ for MnCl_2_ (Table 2 and Figure S7). These values compare favorably with conventional Mn-based MRI agents reported at 3 T (Table S2), such as Mn-DPDP (1.5 mM^–1^.s^–1^), Mn-EDTA and Mn-DTPA (2–4 mM^–1^.s^–1^), macrocyclic chelates such as Mn-NOTA or Mn-PyC3A (2.8–4.2 mM^–1^.s^–1^), and even Mn-porphyrins (5–10 mM^–1^.s^–1^)*.* ^23^ While MnCl_2_ displays relaxivity values comparable to some linear and macrocyclic chelates, it suffers from rapid clearance and nonselective biodistribution.^42^ All three structures maintained consistent *r*_1_ values (variation <0.2%) and exhibited no detectable spectral or mass changes over 1 week, confirming that the Mn centers remain tightly coordinated and that the architectures are kinetically and thermodynamically stable under physiological conditions (Table S3).

To further characterize the imaging profile of these Mn-based structures, we evaluated their transverse relaxivities (r_2_) at 3 T and calculated the *r*_2_/*r*_1_ ratios (Table 2). The *r*_2_/*r*_1_ ratio is widely used to indicate whether a contrast agent behaves predominantly as a *T*_1_ (positive) or *T*_2_ (negative) agent. MnCl_2_ exhibited a high ratio of 11.05, consistent with its established *T*_2_-weighted behavior.^43,44^ In contrast, Mn-[2]C and Mn-TK showed intermediate ratios of 6.6 and 8.8, suggesting mixed but still *T*_1_-dominant characteristics. Notably, Mn-BR displayed the lowest ratio of 5.5, identifying it as the most *T*_1_-selective compound in the series. These findings confirm that the topologically complex Mn structures, particularly Mn-BR, are well suited for bright *T*_1_-weighted imaging applications.

The superior relaxivity of Mn-[2]­C, Mn-TK, and Mn-BR can be attributed to several factors. First, their water solubility and chemical inertness at physiological pH of 7.4 prevent the premature release of Mn^2+^, ensuring sustained imaging efficacy.^21^ Second, these structures exhibit relatively fast water exchange kinetics, essential for optimizing MRI contrast. This rapid exchange contributes to the inner-sphere relaxivity effect, which arises from the incomplete coordination environment of the Mn^2+^ ion, allowing for direct interaction with a water molecule. Furthermore, the outer-sphere component, which results from the diffusion of bulk water molecules around the framework, may also provide a sizable contribution.^21^ The architecture of these molecules further contributes to their high performance. The rigid coordination environment, imparted by the pyridine chelating and π-π stacking interactions, reduces internal molecular motion and increases relaxivity.^45,46^ Furthermore, an increase in signal intensity is observed as the number of Mn^2+^ centers increases from 2 (Mn-[2]­C) to 3 (Mn-TK) to 6 (Mn-BR). This trend can be attributed to the increase in molecular size, which slows rotational dynamics in solution. At a magnetic field of 3T, the rotational correlation time (τ_R_) is generally a limiting factor for the inner-sphere relaxivity contribution, provided water exchange is sufficiently fast. As τ_R_ generally increases with molecular weight, relaxivity follows the sequence Mn[2]C < Mn-TK < Mn-BR.^21^ Additionally, the high positive electrostatic potentials of these structures (Figure 1c) may result in a significant second-sphere contribution due to strong hydrogen bonding networks involving water molecules.^46^ Finally, the progressive decrease in Mn–O water distances with increasing the number of metal centers, as suggested by the computationally derived geometries of the three metal–organic structures, has a minor impact on water exchange, which remains fast enough to facilitate high relaxivities (Table 1).^30^ Collectively, these features underscore the potential of Mn-templated topological architectures as high-performance MRI contrast agents, surpassing current clinical standards.

The relaxivity of Mn-[2]­C, Mn-TK, and Mn-BR is significantly influenced by their interaction with biological macromolecules. When exposed to a bovine serum albumin solution (BSA, 0.67 mM) at pH 7.4, the relaxivity values increased to 7.4, 8.9, and 11.8 Mn mM^–1^.s^–1^ for Mn-[2]­C, Mn-TK, and Mn-BR, respectively (Table 2 and Figure S8). For comparison, MnCl_2_ under identical conditions showed a relaxivity of 9.7 mM^–1^.s^–1^, confirming that macromolecular binding can significantly enhance the relaxivity of even simple Mn^2+^ salts. This increase is attributed to low-affinity protein interaction, which slows down the overall tumbling of the Mn structures. In vitro, such behavior may be explained by the noncovalent interaction of Mn^2+^ structures with serum albumin, known to affect the effective rotational correlation time (τ_R_) of Mn^2+^ complexes.^21,47,48^ The percentage increase in relaxivity is inversely proportional to the size of the Mn-structure, with Mn-[2]­C, Mn-TK, and Mn-BR exhibiting 37, 31, and 17%, respectively. This strong affinity for albumin is advantageous, as it extends the circulation time of the contrast agents, enhances their distribution to tissues, and improves relaxivity even in plasma.^21,47,48^

The relaxivity of the Mn^2+^ structures not only enhances contrast under neutral pH conditions but also exhibits significant alterations in acidic environments, a crucial factor for targeted anticancer applications.^30–32^ At pH 5.4, a pronounced reduction in relaxivity was observed, with decreases of approximately 32% for Mn-TK and 64% for Mn-BR (Table 2 and Figure S9). Under the same conditions, MnCl_2_ showed a smaller ∼40% decrease, serving as the acidic-medium baseline for simple Mn^2+^ salts. This decrease is attributed to the dissociation of the structures at lower pH, and the subsequent formation of aqua structures of the metal ion, as confirmed by mass spectrometry (Figure S1) and UV–vis spectroscopy (Figure S3). Such pH-sensitive disassembly is particularly advantageous for therapeutic applications, where controlled release of metal ions within the acidic tumor microenvironment is desirable.^49–52^ The structural lability of Mn-TK and Mn-BR under mildly acidic conditions supports their capacity to release bioactive Mn^2+^ selectively at tumor sites, thereby enhancing therapeutic efficacy while minimizing systemic toxicity.^53^ In contrast, Mn-[2]­C exhibited only minimal changes in relaxivity at pH 5.4, confirming slower hydrolysis kinetics and greater stability in acidic environments. The enhanced stability may limit the therapeutic utility of Mn-[2]­C compared to Mn-TK and Mn-BR in applications where controlled and rapid metal ion release is crucial.

Incorporating manganese into molecular knots and links offers a dual advantage for targeted cancer therapy: a pH-responsive release mechanism and simultaneous MR imaging capability. These combined functionalities are foundational for the development of effective theranostic agents. Given that the Mn-based nontrivial structures have both of these critical features, we investigated their selective cytotoxicity and imaging performance in glioblastoma cells and noncancerous cells (HEK-293) to determine their potential as dual-function therapeutic.

### 2.4 In Vitro Theranostic Applications

Glioblastoma is among the most aggressive and lethal brain cancers, with poor prognosis attributed to a combination of late-stage diagnosis, high postsurgical recurrence, chemoresistance, tumor heterogeneity, and the challenge of distinguishing postsurgical necrosis from true disease progression.^54^ Early and accurate diagnosis is essential to improving outcomes and can be significantly improved through MRI, which uses specific or nonspecific contrast agents to enhance tumor delineation. These agents improve tumor delineation by enhancing contrast between tumors and healthy tissue and within various tumor components, including edema, necrosis, and blood vessels.^55^ The efficacy of contrast agents is most pronounced in well-vascularized tumors, where increased perfusion enhances imaging resolution. Effective glioblastoma treatment relies on the successful delivery of therapeutic agents to the tumor site, which remains a major hurdle due to the restrictive nature of the blood–brain barrier (BBB). Overcoming this dual challenge of imaging precision and therapeutic delivery has become a central focus in glioblastoma research, motivating the development of integrated theranostic platforms capable of crossing the BBB, targeting tumors, and providing both diagnostic and therapeutic benefits. In this context, U251-MG cells were selected as an in vitro model because they are a well-established and clinically relevant glioblastoma line, widely used to study aggressive growth, therapeutic resistance, and brain tumor biology.

#### 2.4.1 Magnetic Resonance Imaging and Cytotoxicity Analysis of Metal–Organic Compounds in U251-MG and HEK-293 Cells

The differentiation between cancerous and noncancerous tissue in MRI diagnostics depends significantly on variations in *T*_1_ relaxation times, which are influenced by factors such as increased tissue hydration in tumors, reduced intracellular water organization in malignant tissue, and altered interactions between water molecules and cellular macromolecules.^56^ However, despite these inherent differences, the contrast between pathological and normal tissue remains insufficient, limiting the effectiveness of MRI for tumor detection and characterization^56^

To assess *T*_1_ contrast enhancement, we compared the performance of manganese-based structures in glioblastoma (U251-MG) and noncancerous HEK-293 cells, the latter serving as a low-tumorigenic control. Cells were incubated with 10 μM Mn of each structure for 24 h, and *T*_1_ relaxation times were measured on a 3.0 T MRI scanner, with MnCl_2_ included as a monomeric clinical benchmark. The results (Figure 2a,b) revealed pronounced, cancer-selective signal enhancement: relaxation rate increases in U251-MG cells reached 43.4 ± 8.7% for Mn-[2]­C, 69.5 ± 8.7% for Mn-TK, and 139.1 ± 13.0% for Mn-BR (*p* < 0.0001), while HEK-293 cells showed only modest, nonsignificant changes. By comparison, MnCl_2_ produced minimal and nonselective enhancement in both cell types. These findings highlight the importance of topological complexity in promoting tumor-selective uptake and targeted *T*_1_ contrast enhancement.

**Figure 2:**
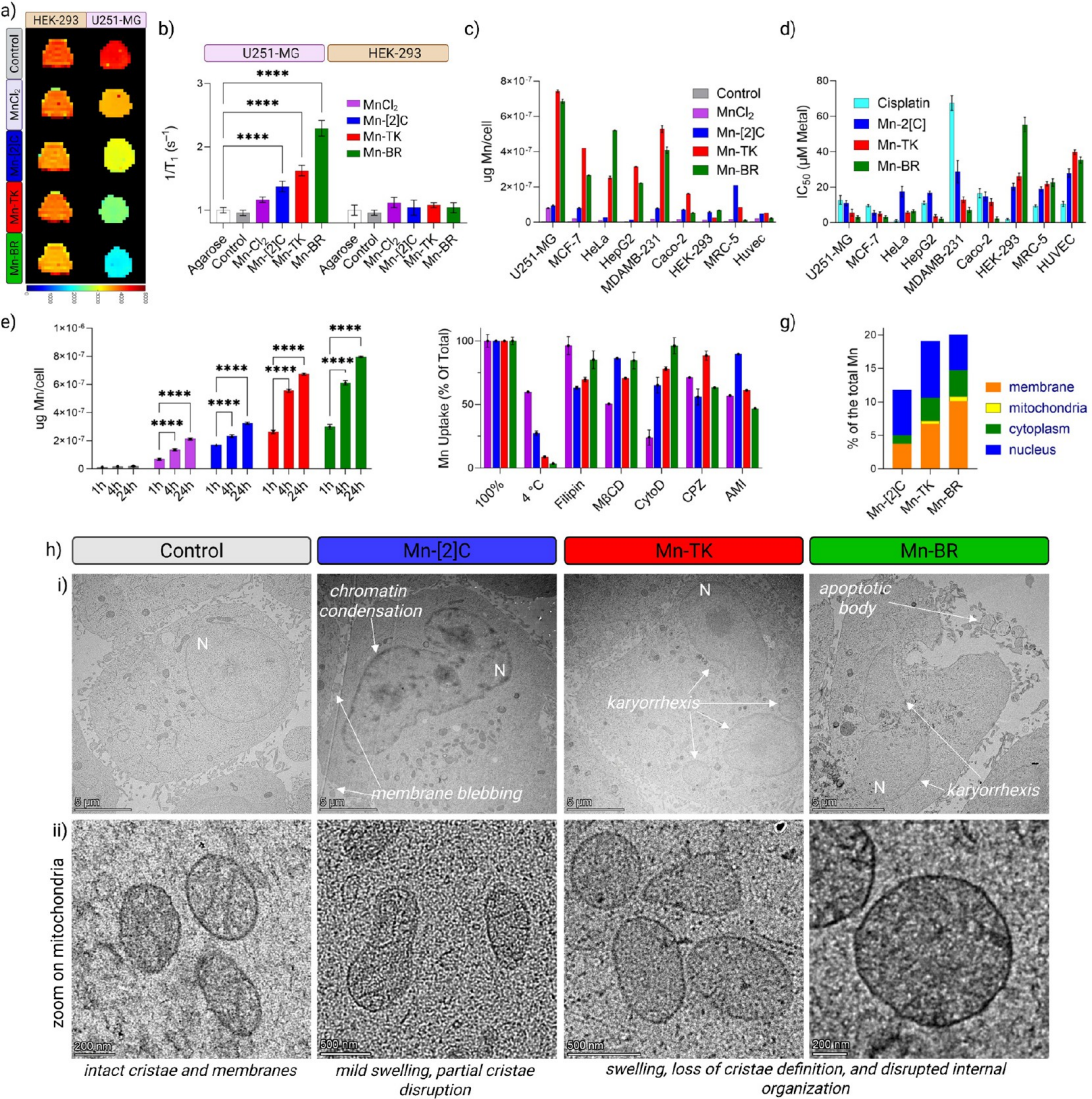
In vitro theranostic evaluation of Mn-based topological structures. (a) Representative *T*_1_ maps (3 T) of U251-MG and HEK-293 cell pellets after 24 h incubation with MnCl_2_, Mn-[2]­C, Mn-TK, or Mn-BR (10 μM Mn, shorter *T*_1_ = stronger contrast). (b) Quantified relaxation rate 1/*T*_1_ (s^–1^, 3 T) in U251-MG, strongest for Mn-TK/Mn-BR, with minimal effect in HEK-293 (mean ± SD, *n* = 3; *p* < 0.05, *p < 0.01, **p < 0.001 vs control). (c) ICP-MS intracellular Mn (10 μM Mn, 24 h) across cancer and normal lines incubated with MnCl_2_, Mn-[2]­C, Mn-TK, or Mn-BR. (d) IC_50_ (48 h) comparison of Mn-[2]­C, Mn-TK, Mn-BR, and cisplatin across cancer and normal lines. (e) Time-dependent cellular uptake of Mn-based compounds in U251-MG glioblastoma cells. Cells were treated with MnCl_2_, Mn-[2]­C, Mn-TK, or Mn-BR (10 μM Mn) for 1, 4, and 24 h, and intracellular Mn was quantified by ICP-MS. Data are mean ± SD (*n* = 3); ****p < 0.0001 vs 1h. (f) Endocytosis-inhibitor assay (4 h, 10 μM): energy depletion (4 °C) and disruption of rafts/caveolae (filipin, MβCD), clathrin (CPZ), or macropinocytosis (amiloride, cytochalasin D) reduce uptake of Mn-TK/Mn-BR, consistent with active endocytic entry (mean ± SD, *n* = 3; *p* < 0.05 to ***p < 0.0001). (g) Subcellular localization of manganese by ICP-MS: Manganese distribution within various subcellular compartments of U251-MG cells (4 h, 10 μM) incubated with Mn-[2]­C, Mn-TK, or Mn-BR. Quantified components include the cytoplasm, mitochondria, nucleus, and lipid-rich debris, with the latter comprising mainly cell membrane fragments and other lipid-rich cellular components. (h) TEM: (i) whole-cell views show chromatin condensation, membrane blebbing, karyorrhexis, and apoptotic bodies after treatment; (ii) higher-magnification images reveal intact mitochondria in control, mild swelling with Mn-[2]­C, and severe swelling/cristae loss with Mn-TK/Mn-BR.

Following the MRI studies, manganese uptake was quantified by ICP-MS after 24 h of incubation with each structure at 10 μM Mn (Figures 2c and S11). In U251-MG cells, uptake levels followed the order Mn-BR > Mn-TK > Mn-[2]­C, mirroring their *T*_1_ relaxivity profiles. Mn-BR and Mn-TK accumulated significantly more Mn than Mn-[2]­C. In contrast, HEK-293 cells showed minimal Mn accumulation, confirming tumor-preferential uptake. This trend was consistent across additional cancer cell lines (MCF-7, HeLa, HepG2, MDA-MB-231, Caco-2) and normal (MRC-5, HUVEC) cell lines, where malignant cells exhibited greater uptake of Mn-BR and Mn-TK compared to Mn-[2]­C. MnCl_2_, by contrast, displayed uniformly low and nonselective cellular accumulation across all tested lines.

Cytotoxicity screening across nine human cell lines revealed a clear structure- and cell-type–dependent hierarchy (Figures 2d and S12, S13, Table 3), with cisplatin included as the clinical benchmark. In U251-MG, Mn-BR (3.0 ± 0.9 μM) and Mn-TK (5.6 ± 1.9 μM) both outperformed cisplatin (12.7 ± 2.5 μM) and were significantly more potent than Mn-[2]­C. This trend extended to other cancer types: in HepG2, Mn-BR (2.1 ± 0.8 μM) and Mn-TK (3.6 ± 0.8 μM) showed superior activity compared to cisplatin (11.0 ± 0.9 μM) and Mn-[2]­C; in Caco-2, Mn-BR (2.3 ± 0.9 μM) remained highly active relative to cisplatin (16.5 ± 2.6 μM) and Mn-[2]­C. Notably, in drug-resistant MDA-MB-231, Mn-BR (7.1 ± 1.4 μM) and Mn-TK (12.9 ± 1.5 μM) retained considerable activity, whereas cisplatin (67.5 ± 4.2 μM) was largely ineffective and Mn-[2]C showed limited potency.

**Table 3:** IC_50_ Values (μM Metal, Mean ± SD) of Cisplatin, Mn-[2]­C, Mn-TK, and Mn-BR across Cancer and Normal Cell Lines, with Mean IC_50_, Selectivity Indices, and Hemolysis Percentages

IC_50_ (μM, mean ± SD)	cisplatin	Mn-[2]C	Mn-TK	Mn-BR
U251-MG	12.70 ± 2.50	11.01 ± 1.40	5.60 ± 1.90	3.00 ± 0.90
MCF-7	9.40 ± 0.65	5.54 ± 1.30	4.90 ± 1.10	3.12 ± 0.80
HeLa	1.04 ± 0.70	17.40 ± 3.00	5.70 ± 0.60	6.40 ± 0.90
HepG2	11.04 ± 0.90	16.80 ± 1.05	3.60 ± 0.80	2.14 ± 0.80
MDA-MB-231	67.50 ± 4.20	28.70 ± 6.30	12.90 ± 1.50	7.09 ± 1.40
Caco-2	16.49 ± 2.60	14.76 ± 2.50	11.60 ± 1.90	2.31 ± 0.90
HEK-293	1.80 ± 0.50	20.27 ± 1.96	26.09 ± 1.90	55.30 ± 4.10
MRC-5	9.30 ± 0.60	18.90 ± 1.30	21.90 ± 1.20	22.60 ± 2.10
HUVEC	10.40 ± 1.40	27.90 ± 2.40	39.70 ± 1.30	35.30 ± 1.70
mean IC_50_ (cancer, μM)	19.7	15.7	7.4	4
mean IC_50_ (normal, μM)	7.2	22.4	29.2	37.7
selectivity index (normal/cancer)	0.36	1.42	3.96	9.41
hemolysis (%)	∼14%	<5%	<5%	<5%

Therapeutic windows were assessed by the selectivity index SI defined as the ratio of the mean IC_50_ in normal cells to the mean IC_50_ in cancer cells. The calculation included the three normal (HEK-293, MRC-5, HUVEC) and six cancer lines (U251-MG, MCF-7, HeLa, HepG2, MDA-MB-231, Caco-2) (Table 3). Mn-BR showed the broadest therapeutic window with an SI of 9.41 (37.7 μM in normal cells vs 4.0 μM in cancer cells). Mn-TK also demonstrated favorable selectivity, with an SI of 3.96 (29.2 μM vs 7.4 μM). In contrast, Mn-[2]­C showed only a modest selectivity, with an SI of 1.42 (22.4 μM vs 15.7 μM). Notably, cisplatin displayed inverse selectivity with an SI of 0.36 (7.2 μM vs 19.7 μM), indicating a narrow therapeutic window.

Taken together, these results establish a consistent performance hierarchy: Mn-BR > Mn-TK ≫ Mn-[2]C ≈ cisplatin. Mn-BR and Mn-TK clearly outperform both Mn-[2]C and the clinical benchmark cisplatin across multiple criteria, including cellular uptake, cytotoxic potency, selectivity, and biocompatibility, underscoring the advantages conferred by topological design. While Mn-[2]C demonstrates modest activity and weak selectivity, it still provides a broader therapeutic window than cisplatin.

To complement the cytotoxicity studies, hemocompatibility was assessed using a standard hemolysis assay with human red blood cells (Table 3, Figure S14). Mn-TK and Mn-BR exhibited hemolysis rates below 5%, meeting the ASTM F756–08 criteria for nonhemolytic materials.^57–59^ Mn-[2]C showed similarly low hemolysis. In stark contrast, cisplatin induced ∼14% hemolysis,^60^ underscoring its known systemic toxicity. Collectively, these results confirm the excellent blood compatibility of our Mn-based nontrivial structures and further support their suitability for intravenous administration.

We next sought to clarify whether the distinct contrast and cytotoxic behaviors stem from differences in cellular uptake.

#### 2.4.2 Mechanistic Basis of Cellular Uptake, Subcellular Localization, and Selectivity

The differences in contrast enhancement and cytotoxicity between cancerous and noncancerous cells exposed to these metal–organic structures can largely be attributed to variations in cellular uptake and retention mechanisms.^61,62^ Cancer cells exhibit enhanced permeability compared to normal cells due to several distinctive features, including a higher surface charge density, thinner cell membranes, and a faster turnover rate.^63–65^

To investigate internalization and trafficking, we used kinetic uptake measurements, pathway-specific pharmacological inhibition, and subcellular ICP-MS fractionation. The results were then interpreted in the context of molecular size, surface electrostatic potential, and experimentally determined lipophilicity.

##### 2.4.2.1 Kinetic Uptake Profiles

ICP-MS quantification of intracellular manganese following treatment with 10 μM Mn revealed distinct, topology-dependent uptake kinetics among the three Mn-based complexes and MnCl_2_ in U251-MG glioblastoma cells (Figure 2e). Manganese accumulation increased progressively over time for all compounds, following the order Mn-BR > Mn-TK > Mn-[2]­C > MnCl_2_ > control. After 1 h, Mn-BR showed the fastest uptake, with accumulation 1.7-fold higher than Mn-TK, 1.8-fold higher than Mn-[2]­C, and 4.3-fold higher than MnCl_2_. At 4 h, uptake continued to rise for all topologies while maintaining the same relative ranking. After 24 h, both Mn-BR and Mn-TK reached near-saturation levels, clearly exceeding Mn-[2]C and MnCl_2_. These final values correspond to 3.8-fold (Mn-BR) and 3.2-fold (Mn-TK) higher accumulation relative to MnCl_2_, whereas Mn-[2]C remained only 1.5-fold above the ionic baseline. These results demonstrate a clear time- and topology-dependent uptake mechanism, with Mn-BR showing the fastest internalization and highest intracellular retention across all time points, correlating with its superior MRI contrast performance and enhanced therapeutic activity.

##### 2.4.2.2 Endocytic Inhibitor Assays

The cellular pathways underlying these kinetic differences were examined using energy depletion and pharmacological inhibition (Figures 2f and S15). At 4 °C, the uptake of Mn-TK and Mn-BR was strongly reduced to 8.8 and 3.5% of control, respectively, confirming that their internalization is strictly energy-dependent and requires active endocytic processes. In comparison, Mn-[2]C and MnCl_2_ also showed clear temperature sensitivity, decreasing to 27.4 and 59.9% of the control, respectively, indicating partial reliance on energy-dependent transport and surface interactions in addition to passive diffusion. Further dissection using pathway-specific inhibitors revealed distinct internalization routes for the topological complexes. Uptake of Mn-TK was only modestly affected by chlorpromazine (88.4% of control), suggesting minimal involvement of clathrin-mediated endocytosis. In contrast, filipin (69.7% of control) and methyl-β-cyclodextrin (70.6% of control) produced stronger inhibition, pointing to a dominant contribution from caveolae- and lipid raft–mediated processes. For Mn-BR, the most pronounced reductions were caused by chlorpromazine (63.3% of control) and amiloride (46.9% of control), indicating primary dependence on clathrin-mediated and macropinocytic uptake. Filipin and MβCD had comparatively minor effects (85.2 and 84.6% of control), implying limited involvement of caveolae or raft-associated entry routes. By contrast, Mn-[2]C and MnCl_2_ exhibited broad but nonspecific inhibition across several conditions (50–90% of control), consistent with low internalization efficiency and the absence of any dominant endocytic mechanism.

These results collectively demonstrate that Mn-TK enters cells mainly through caveolae- and lipid-raft–mediated endocytosis, whereas Mn-BR relies primarily on clathrin- and macropinocytosis-driven uptake. Both pathways are energy-dependent and typically upregulated in malignant cells, which helps explain the preferential accumulation of Mn-BR and Mn-TK in tumor tissues and their limited uptake in normal cells.^61,62,66,67^

##### 2.4.2.3 Subcellular Localization

To determine the intracellular fate of the manganese-based structures, we performed cell fractionation followed by ICP-MS quantification in U251-MG cells after 24 h of exposure to 10 μM of each compound (Figure 2g). Manganese levels were measured in the cytoplasm, mitochondria, nucleus, and lipid-rich debris, the latter consisting of membranes, endoplasmic reticulum, lysosomal vesicles, and peroxisomal fragments, making it a sensitive indicator of organelle-associated accumulation. Mn-BR showed predominant localization in the lipid-rich fraction (10.1%), followed by the nucleus (5.3%), cytoplasm (3.9%), and mitochondria (0.7%). Mn-TK exhibited dual accumulation in the nucleus (8.5%) and lipid-rich debris (6.7%), with moderate levels in the cytoplasm (3.4%) and mitochondria (0.5%). In contrast, Mn-[2]­C accumulated predominantly in the nucleus (6.8%) with lower levels in lipid fractions (3.7%) and minimal amounts in mitochondria (0.1%) and cytoplasm (1.2%). These data suggest that Mn-BR and Mn-TK preferentially associate with membrane-rich organelles.

##### 2.4.2.4 Mechanistic Interpretation and Selectivity

Our findings establish that the physicochemical properties of the Mn-based structures—specifically molecular size, electrostatic potential, and lipophilicity—govern both their cellular uptake mechanisms and their selectivity toward cancer cells. Mn-[2]C is a relatively small cation (1396 Da, + 0.2 au, corresponding to 125.5 kcal·mol^–1^) with the lowest lipophilicity (log *P* = −0.80). This profile favors nonspecific passive diffusion across membranes and weak electrostatic interaction with the cell surface, resulting in a diffuse, nonselective intracellular distribution similar to that of MnCl_2_. The absence of efficient active uptake limits its tumor-targeting capability and therapeutic performance. In contrast, Mn-TK (2418 Da, + 0.4 au, 251.0 kcal.mol^–1^, log *P* = −0.46) and Mn-BR (4668 Da, + 0.6 au, 376.5 kcal.mol^–1^, log *P* = −0.49) are larger, more electropositive, and moderately more lipophilic. These characteristics promote preferential interactions with the negatively charged, fluid membranes of cancer cells. Mn-TK internalizes predominantly through caveolae- and lipid raft–mediated endocytosis, with minimal contribution from clathrin-mediated uptake. Mn-BR, owing to its higher Mn coordination number, multivalency, and stronger positive surface potential, is mainly internalized via clathrin-mediated endocytosis and macropinocytosis, with only minor participation of caveolae- or raft-dependent routes. Since clathrin-mediated and macropinocytic pathways are strongly upregulated in malignant cells but largely quiescent in normal tissues, these topology-specific entry mechanisms explain the pronounced tumor selectivity observed, particularly for Mn-BR.^63–65^

Subcellular fractionation further confirmed that Mn-TK and Mn-BR accumulate in lipid-rich membrane and organelle fractions, consistent with their localization at membrane–organelle interfaces. Lysosomal trafficking subsequently promotes pH-dependent degradation and Mn^2+^ release, initiating mitochondrial dysfunction and apoptosis. In contrast, Mn-[2]C lacks these features, remains poorly accumulated, and exhibits nonselective behavior.

In summary, Mn-BR functions as a rapid-uptake, clathrin/macropinocytosis-driven agent; Mn-TK as a moderate-uptake, caveolae/lipid-raft–dependent agent; and Mn-[2]C as a low-uptake, diffusion-limited agent. This classification provides a coherent mechanistic framework linking their topological structure to differential selectivity, MRI contrast, and therapeutic efficacy.

#### 2.4.3 Morphological and Ultrastructural Changes Analysis Using TEM

To study the morphological and ultrastructural changes associated with cell death, we performed transmission electron microscopy (TEM) on U251-MG cells treated for 4 h with Mn-[2]­C, Mn-TK, or Mn-BR (Figures 2h and S16, S17). In untreated controls, we observed intact nuclei, elongated mitochondria with well-preserved cristae, and smooth plasma membranes. In contrast, all treated groups exhibited features characteristic of apoptosis, including membrane blebbing, cytoplasmic vacuolization, and formation of apoptotic bodies. These features were especially prominent in cells exposed to Mn-TK and Mn-BR. Notably, plasma membranes remained intact in all samples, ruling out necrosis and supporting a regulated apoptotic mechanism.

At the nuclear level, Mn-[2]C treatment led to chromatin condensation and marginalization along the nuclear envelope, which are recognized hallmarks of early apoptosis (Figures 2h and S16).^68^ In contrast, Mn-TK and Mn-BR induced more advanced nuclear alterations, including dense chromatin clumping, karyorrhexis, and incorporation of nuclear fragments into apoptotic bodies. These observations indicate a progression from early to late-stage nuclear apoptosis.^68^

Mitochondria were also significantly affected in a structure-dependent manner. While mitochondria in control cells retained intact cristae and double membranes, Mn-[2]C induced swelling, partial cristae disruption, and vesicular transformations, all indicative of early mitochondrial stress.^68^ Cells treated with Mn-TK and Mn-BR exhibited much more severe alterations, including extensive swelling, cristae depletion, and the appearance of ghost-like spherical mitochondria lacking internal architecture. High-magnification TEM confirmed this sequence: Mn-[2]­C-treated cells displayed translucent matrices and fragmented cristae, whereas Mn-TK and Mn-BR treatment resulted in ballooned mitochondria with empty interiors, consistent with advanced apoptotic collapse. Quantitative analysis further supported these findings. Mitochondria in control cells averaged 550 ± 7 nm in diameter. This increased to 604 ± 27 nm after Mn-[2]­C treatment, and further to 799 ± 23 nm and 787 ± 13 nm for Mn-TK and Mn-BR, respectively (Figures 2h-ii and S17, S18). The increased mitochondrial size corresponds with progressive apoptotic damage. Additionally, Mn-TK and Mn-BR treatment resulted in a 1.7-fold increase in overall cell diameter compared to control, reinforcing their strong apoptotic potential.^68^

In addition to nuclear and mitochondrial alterations, cells treated with Mn-based structures exhibited pronounced cytoplasmic vesiculation. Large intracellular vacuoles and vesicles were evident, particularly in Mn-TK- and Mn-BR-treated cells, suggesting lysosomal and autophagic involvement in the apoptotic cascade. Notably, budding cytoplasmic fragments detaching from the main cell body, hallmarks of apoptotic body formation, were observed in these treatment groups, indicating progression to the terminal stages of apoptosis.

Collectively, these TEM analyses confirm that Mn-[2]­C, Mn-TK, and Mn-BR each induce a distinct but overlapping spectrum of apoptotic features in U251-MG cells. These include early chromatin condensation and mitochondrial swelling to advanced nuclear fragmentation, mitochondrial collapse, and apoptotic body formation. While these ultrastructural changes strongly implicate apoptosis as the primary mode of cell death, the biochemical basis of this process, including ROS accumulation, mitochondrial depolarization, and caspase activation, is addressed through complementary assays presented below.

#### 2.4.4 Detection of Apoptosis in U251-MG Glioma Cells Treated with Metal–Organic Structures

Apoptosis is a highly regulated form of programmed cell death that enables the selective elimination of cancer cells while preserving healthy tissue and minimizing necrosis-related inflammation. Following the ultrastructural evidence of apoptosis observed by TEM, we sought functional validation that apoptosis is the predominant mechanism of cell death triggered by the Mn-based nontrivial structures. To this end, we conducted a combination of Annexin V assays and caspase-3 activity measurements. Cisplatin was included as a clinically approved apoptotic agent for benchmarking, and MnCl_2_ was used as a negative control to represent a nonspecific, low-toxicity Mn^2+^ source.

##### 2.4.4.1 Apoptosis as the Predominant Death Mechanism

Annexin V/PI flow cytometry performed after 24 h treatment at 10 μM (Figure 3a) demonstrated a marked and statistically significant increase in apoptotic cell populations following Mn-TK and Mn-BR treatment. Untreated control cells exhibited low basal apoptosis (7.35% total apoptotic; 2.99% early and 4.36% late). Cisplatin induced moderate apoptosis (12.15% total), while Mn-[2]C produced a modest increase (14.76% total apoptotic cells). In contrast, Mn-TK and Mn-BR triggered pronounced apoptosis, reaching 58.70 and 60.90% total apoptotic cells, respectively, driven predominantly by early apoptosis. Necrotic populations remained low across all treatment groups (≤3.6%), confirming that cell death occurred primarily through apoptosis rather than nonspecific necrosis. To further confirm these results, a live-cell Annexin V assay was performed with real-time quantification at 24 and 48 h (Figures 3b and S20). Both Mn-TK and Mn-BR induced robust, statistically significant, and time-dependent increases in Annexin V fluorescence relative to Mn-[2]­C, MnCl_2_, and controls (****p < 0.0001, *n* = 3). Notably, Mn-BR and Mn-TK surpassed cisplatin in apoptotic signal intensity by 24 h, and both remained significantly more potent than cisplatin at 48 h. In contrast, Mn-[2]­C showed a modest increase at 24 h and remained below cisplatin at 48 h. These results demonstrate that Mn-TK and Mn-BR induce rapid and sustained apoptosis, with a potency that exceeds cisplatin. Western blot analysis further provided molecular confirmation (Figure 3c). After 24 h treatment, total caspase-3 levels decreased to 56.2% for Mn-TK and 71.2% for Mn-BR relative to GAPDH-normalized controls (100%), while Mn-[2]C reduced caspase-3 to 71.0%. Caspase-8 levels were similarly reduced (≈78–81% of control), consistent with caspase activation and proteolytic processing. Together with the Annexin and ultrastructural alterations observed by TEM, these results confirm that caspase-dependent apoptosis is the primary cytotoxic pathway engaged by the Mn-based topological structures, with Mn-TK and Mn-BR emerging as the most potent apoptotic inducers.

**Figure 3:**
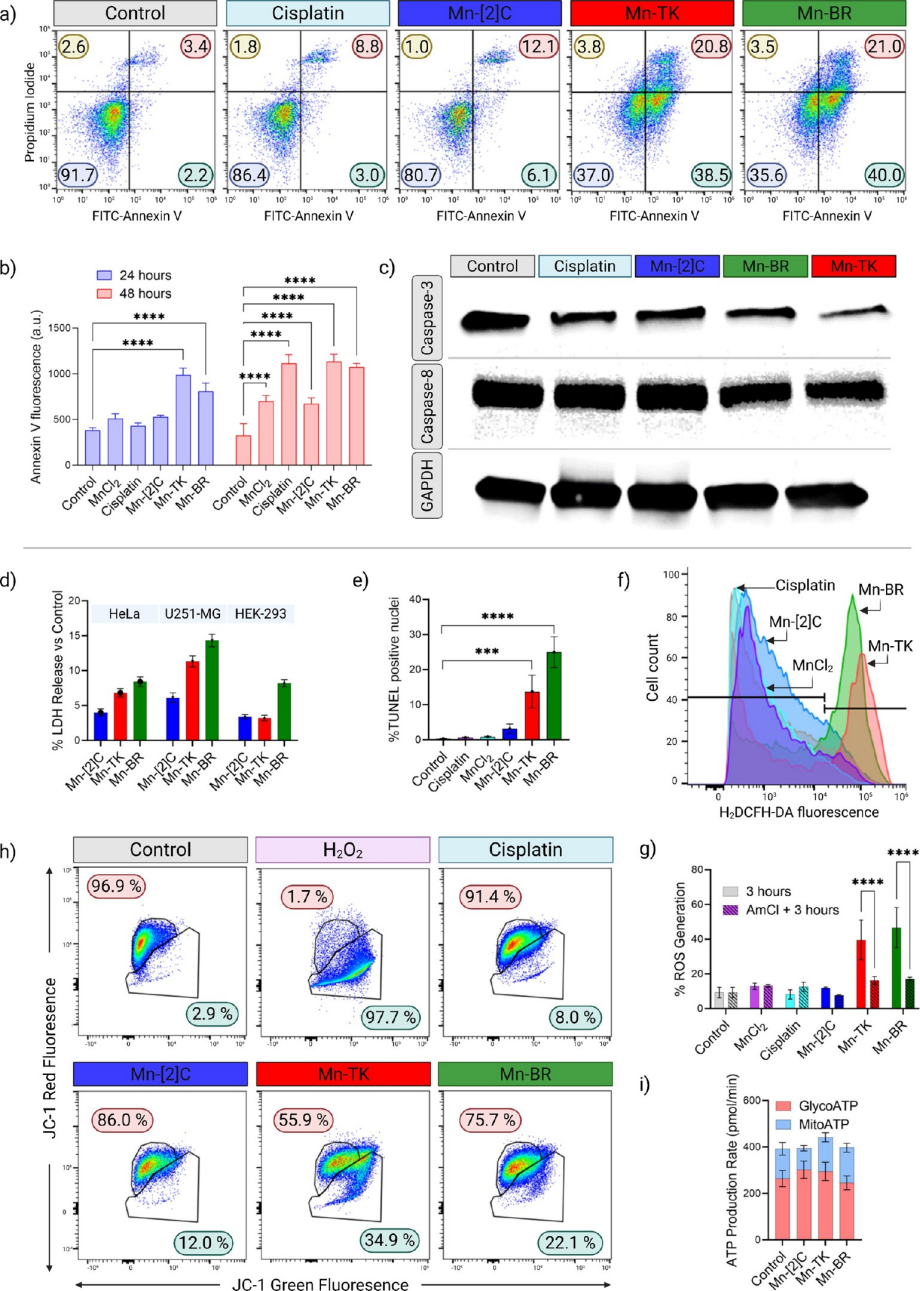
Mn topologies trigger mitochondria-centered, ROS-driven apoptosis in U251-MG cells. (a) Annexin V-FITC and PI staining: Flow cytometry results depicting the effects of treatments Mn-[2]­C, Mn-TK, or Mn-BR and cisplatin (10 μM Mn or equivalent drug concentration) on U251-MG cell apoptosis. The quadrants indicate live cells (lower left), early apoptotic cells (lower right), late apoptotic cells (upper right), and necrotic cells (upper left). This assay was performed in triplicate. (b) Quantification of Annexin V fluorescence (a.u.) at 24 and 48 h; Mn-TK and Mn-BR are significantly higher than control and other Mn species (mean ± SD, *n* = 3; ****p < 0.0001). (c) Western blot analysis of apoptosis-related proteins in U251-MG cells. Representative immunoblots showing total caspase-3 and caspase-8 expression in U251-MG cells treated for 24 h with cisplatin, Mn-[2]­C, Mn-TK, or Mn-BR (10 μM Mn or equivalent drug concentration). GAPDH was used as a loading control. A reduction in total caspase levels relative to control indicates caspase activation and apoptotic processing. Blots are representative of three independent experiments. (d) LDH release after 24 h (10 μM Mn) in U251-MG, HeLa, and HEK-293 (mean ± SD, *n* = 3). (e) TUNEL assay at 3 h of U251-MG cells treated with MnCl_2_, Mn-[2]­C, Mn-TK, Mn-BR, and cisplatin (10 μM Metal, mean ± SD, *n* = 3; ***p < 0.001, **p < 0.0001). (f) H_2_DCFH-DA flow-cytometry histograms at 3 h of U251-MG cells treated with MnCl_2_, Mn-[2]­C, Mn-TK, Mn-BR, and cisplatin (10 μM Metal) reveal rightward fluorescence shifts for Mn-TK and Mn-BR, indicating elevated intracellular ROS. (g) Percentage of ROS-positive cells at 3 h with or without NH_4_Cl preincubation (1 h) shows that NH_4_Cl attenuates ROS induced by Mn-TK/Mn-BR, implicating a lysosome-dependent component (mean ± SD, *n* = 3; ****p < 0.0001). (h) Mitochondrial membrane potential (ΔΨm) assessed by JC-1 staining. Representative flow cytometry plots of JC-1 red (polarized mitochondria) versus green (depolarized mitochondria) fluorescence in U251-MG cells treated for 3 h with cisplatin, Mn-[2]­C, Mn-TK, or Mn-BR (10 μM). H_2_O_2_ served as a positive control for complete depolarization. A shift from red to green fluorescence indicates loss of ΔΨm. Percentages represent polarized versus depolarized cell populations. (i) Seahorse XF ATP-rate analysis at 24 h of U251-MG cells treated with Mn-[2]­C, Mn-TK, Mn-BR shows treatment-specific bioenergetic remodeling: Mn-BR increases the OXPHOS contribution; Mn-TK elevates both mitochondrial and glycolytic ATP (highest total ATP); Mn-[2]C reduces mitochondrial ATP with glycolytic compensation (mean ± SEM, *n* = 3). Statistical tests: one-way ANOVA with appropriate posthoc comparisons; ns, *p* < 0.05, *p < 0.01, **p < 0.001, and ***p < 0.0001.

To elucidate the upstream triggers of this apoptotic program, we assessed membrane integrity, DNA fragmentation, oxidative stress, and mitochondrial function.

Plasma membrane integrity was evaluated by measuring lactate dehydrogenase (LDH) release in U251-MG glioblastoma, HeLa, and HEK-293 cells after 24 h treatment with Mn-[2]­C, Mn-TK, and Mn-BR, all at 10 μM in Mn. As shown in Figure 3d, LDH release followed a structure- and cell line–dependent pattern. In U251-MG cells, Mn-BR induced the highest LDH release (+14% relative to control), followed by Mn-TK (+11%), whereas Mn-[2]C remained close to baseline (+6%). A similar but attenuated trend was observed in HeLa cells, with Mn-BR again producing the greatest LDH increase (+8%). In contrast, normal HEK-293 cells showed minimal membrane damage, with Mn-BR causing a modest +8% change and Mn-TK and Mn-[2]C yielding values nearly comparable to the untreated control. LDH release levels across all conditions remained well below those associated with necrosis, indicating that membrane disruption is a late and secondary event rather than the primary mechanism of cytotoxicity. This interpretation is supported by transmission electron microscopy (TEM), which revealed intact plasma membranes during early and midstage apoptosis, despite extensive nuclear fragmentation and mitochondrial damage.

To determine whether the Mn-based nontrivial structures induce DNA damage, we performed a TUNEL assay in U251-MG glioblastoma cells after 3 h of treatment with Mn-[2]­C, Mn-TK, Mn-BR, MnCl_2_ (10 μM in Mn), and cisplatin (10 μM). As shown in Figure 3e, control cells, MnCl_2_, and cisplatin produced minimal DNA fragmentation, with ≤ 1% of nuclei staining positive after 3 h (0.33 ± 0.02%, 0.85 ± 0.14%, and 0.65 ± 0.14%, respectively). Mn-[2]­C caused only a modest increase (3.17 ± 1.31%), consistent with its low cellular uptake and weak cytotoxicity. In contrast, Mn-TK significantly elevated the proportion of TUNEL-positive nuclei to 13.73 ± 4.67%, and Mn-BR induced the most extensive DNA damage, with 24.98 ± 4.39% of cells exhibiting nuclear fragmentation. These structure-dependent differences were statistically significant (***p < 0.001–*p < 0.0001 vs control). The TUNEL results corroborate TEM observations of chromatin condensation, nuclear disintegration, and apoptotic body formation, reinforcing that apoptosis is the primary mechanism of cell death induced by Mn-TK and Mn-BR.

To investigate whether reactive oxygen species (ROS) contribute to the anticancer activity of our Mn-based structures, intracellular ROS levels were quantified in U251-MG glioblastoma cells using DCFDA staining and flow cytometry after 3 h of treatment with Mn-[2]­C, Mn-TK, Mn-BR, MnCl_2_, or cisplatin (Figure 3f,g). Quantitative analysis revealed that untreated cells exhibited baseline ROS levels of 9.2 ± 3.1%. MnCl_2_ (12.8 ± 1.8%) and Mn-[2]C (11.9 ± 0.5%) induced only modest increases relative to control, while cisplatin (8.2 ± 2.7%) remained close to baseline. In contrast, Mn-TK and Mn-BR triggered substantial ROS generation, with levels rising to 39.6 ± 11.4% and 46.7 ± 11.6%, respectively (Figure 3f,g). These values confirm a strong structure-dependent effect, with Mn-BR producing the highest ROS levels, consistent with its superior cytotoxic activity. Elevated ROS is a well-established trigger of apoptosis in cancer cells, particularly in response to redox-active metal complexes capable of Fenton-like and redox cycling reactions. These processes promote mitochondrial depolarization, DNA fragmentation, and caspase activation.^69,70^ In this context, the pronounced ROS induction observed for Mn-TK and Mn-BR likely plays a central role in their cytotoxic mechanism.

To examine the role of lysosomal processing, we pretreated cells with NH_4_Cl (20 mM, 1 h), an inhibitor of lysosomal acidification. This intervention significantly reduced ROS levels generated by Mn-TK and Mn-BR (Figures 3g and S22). In the presence of NH_4_Cl, ROS levels decreased from 39.6 ± 11.4% to 16.3 ± 2.3% for Mn-TK, and from 46.7 ± 11.6% to 17.1 ± 0.9% for Mn-BR, representing more than a 2-fold reduction. In contrast, Mn-[2]­C, MnCl_2_, and cisplatin showed minimal changes, consistent with their lower or non-pH-dependent ROS activity. These results demonstrate that the high ROS levels induced by Mn-TK and Mn-BR are at least partially dependent on acidic lysosomal conditions, which promote Mn^2+^ release. These observations align with our uptake studies showing that Mn-TK and Mn-BR are predominantly internalized through endocytic pathways and trafficked to endo/lysosomes. Collectively, the data support a model in which lysosome-mediated, pH-responsive ROS generation serves as a key upstream event linking molecular topology and physicochemical properties to selective anticancer activity.

Mitochondrial dysfunction emerged as a central component of the cytotoxic mechanism induced by Mn-TK and Mn-BR. JC-1 staining was used to assess mitochondrial membrane potential (ΔΨm) in U251-MG cells after 3 h treatment (10 μM). Control cells exhibited predominantly polarized mitochondria, while H_2_O_2_ induced near-complete depolarization and cisplatin caused a modest shift toward JC-1 green fluorescence, validating the assay. Mn-[2]C produced only a limited depolarization, whereas Mn-TK and Mn-BR induced a pronounced shift toward the JC-1 green-high population, indicating substantial loss of ΔΨm, with Mn-TK showing the strongest effect (Figures 3h and S23). These results are consistent with the ultrastructural TEM observation of swollen, cristae-depleted mitochondria in Mn-TK- and Mn-BR-treated cells, as well as with subcellular ICP-MS subcellular fractionation confirming enrichment of both compounds in lipid-rich and mitochondrial compartments.

Collapse of mitochondrial membrane potential was further corroborated by bioenergetic profiling using the Seahorse XF Real-Time ATP Rate assay (Figure 3i, Table S7). This analysis partitions total ATP production into glycolytic (cytosolic) and mitochondrial (OXPHOS) components, providing insight into energy metabolism and stress responses. Mn-[2]C reduced mitochondrial ATP production by 27.6% (128.4→92.9 pmol.min^–1^) with a compensatory +14.4% increase in glycolytic ATP (264.0→302.0 pmol.min^–1^), resulting in no significant change in total ATP (+0.7%, 392.4→395.0 pmol.min^–1^), consistent with mild mitochondrial impairment buffered by glycolysis. In contrast, Mn-TK increased both mitochondrial ATP (+14.4%, to 146.9 pmol.min^–1^) and glycolytic ATP (+11.7%, to 294.8 pmol.min^–1^), raising total ATP by +12.6% (to 441.7 pmol.min^–1^). The distribution of energy production remained similar to control (33.3% OXPHOS/66.7% glycolysis), indicative of a high-energy, preapoptotic state. Mn-BR treatment redistributed ATP production toward mitochondria, increasing mitochondrial ATP by 17.7% (to 151.1 pmol.min^–1^) and decreasing glycolytic ATP by 6.9% (to 245.9 pmol.min^–1^) while preserving total ATP (+1.2%, reaching 397.0 pmol.min^–1^). The mitochondrial contribution to total ATP rose to 38.1%, compared to 32.5% in control cells.

Taken together, these findings show that Mn-TK and Mn-BR actively engage and stress mitochondria. Initially, this leads to increased or sustained ATP output, followed by mitochondrial membrane depolarization and apoptotic collapse. In contrast, Mn-[2]C exerts a milder mitochondrial effect, effectively counterbalanced by glycolysis. This bioenergetic hierarchy mirrors the results from subcellular localization and Annexin V assays, reinforcing the mechanistic model in which topological structure dictates intracellular trafficking, organelle targeting, and downstream apoptotic responses.

##### 2.4.4.2 Mechanistic Interpretation of Apoptosis Induction

The data collectively support a mitochondria-centered, ROS-driven apoptotic mechanism initiated by the unique structural and physicochemical properties of Mn-TK and Mn-BR. Their larger molecular size, higher Mn content, increased positive surface potential, and moderate lipophilicity enhance their interaction with negatively charged cancer cell membranes. These features promote active internalization through endocytic pathways preferentially upregulated in tumor cells. In contrast, Mn-[2]­C, with its smaller size, less charge, and more hydrophilic, enters cells through a combination of passive and clathrin-mediated mechanisms. This results in a diffuse and less selective intracellular distribution, similar to MnCl_2_. Once internalized, the structural complexity and electrostatic properties of Mn-TK and Mn-BR facilitate trafficking to endolysosomal compartments, as supported by subcellular ICP-MS fractionation showing enrichment in membrane- and vesicle-associated fractions. These acidic vesicles serve as critical processing sites where partial degradation occurs. The chemical composition of these compounds governs their pH-responsive disassembly, enabling controlled release of redox-active Mn^2+^. This lysosomal release step is essential for cytotoxicity, as pharmacological inhibition of acidification significantly reduces ROS generation. The liberated Mn^2+^ ions catalyze the formation of reactive oxygen species through redox cycling and Fenton-like reactions.^69,70^ Elevated ROS acts as the first intracellular stress signal, initiating oxidative damage that destabilizes multiple organelles. Among these, mitochondria are primary targets. ROS compromise the integrity of the mitochondrial inner membrane, leading to depolarization of the mitochondrial membrane potential (ΔΨm) and impairing oxidative phosphorylation. JC-1 staining and Seahorse XF bioenergetic profiling revealed that Mn-TK and Mn-BR engage mitochondria early in the apoptotic cascade. These compounds maintain or transiently increase total ATP levels, with a greater contribution from OXPHOS, suggesting a stressed but active mitochondrial state prior to full collapse. By contrast, Mn-[2]C reduces mitochondrial ATP production, which is offset by increased glycolysis. These distinct profiles reflect varying degrees of mitochondrial engagement and apoptotic commitment.

In parallel, ROS-mediated damage extends to the nucleus. TUNEL assays showed significant DNA fragmentation following treatment with Mn-TK and especially Mn-BR. This nuclear damage aligns with TEM observations of chromatin condensation, marginalization, and karyorrhexis, consistent with downstream apoptotic progression. The culmination of mitochondrial dysfunction, ROS accumulation, and nuclear damage triggers the activation of executioner caspases. Western blot analysis confirmed caspase-3 and 8 cleavages in Mn-TK- and Mn-BR-treated cells, solidifying their role in caspase-dependent apoptosis.

Importantly, LDH assays and TEM analysis confirmed that plasma membranes remained intact during early and intermediate phases of treatment. Membrane disruption occurred only during late-stage apoptosis with the formation of apoptotic bodies. This indicates that necrosis is not the dominant mode of cell death. The superior efficacy of Mn-BR relative to Mn-TK can be directly linked to its Borromean ring topology. This structure confers higher manganese loading, greater electrostatic potential, and increased lipophilicity, all of which enhance membrane association, endocytic uptake, lysosomal Mn^2+^ release, and subsequent ROS generation. These properties result in stronger mitochondrial disruption and faster apoptotic execution. Mn-TK follows the same mechanistic cascade with moderate intensity, while Mn-[2]­C lacks the necessary features to initiate this sequence effectively.

In summary, Mn-TK and Mn-BR trigger a topologically regulated, mitochondria-centered apoptotic program in glioblastoma cells. Their architectures promote selective uptake, lysosomal disassembly, Mn^2+^ release, ROS accumulation, mitochondrial depolarization, DNA fragmentation, and caspase activation. This mechanistic pathway underpins their tumor selectivity and distinguishes them from conventional agents such as MnCl_2_ and cisplatin, offering a promising foundation for the development of structurally defined theranostic agents.

Growth curve analysis further examined the effects of Mn-[2]­C, Mn-TK, and Mn-BR on U251-MG glioma cell proliferation. The results showed marked differences in growth rates (Figure S24 and Table S8). Mn-BR treatment increased doubling time to 148.2 h compared to the 34.66 h of the control, while Mn-TK treatment led to severe growth arrest, with a doubling time of 837.8 h. Mn-[2]­C treatment moderately inhibited proliferation, increasing doubling time to 69.49 h. These results demonstrate the potent antiproliferative effects of Mn-TK and Mn-BR, further highlighting their potential as targeted therapeutic agents for glioma treatment.

The distinct physicochemical properties of Mn-TK and Mn-BR contribute to their enhanced therapeutic efficacy, as evidenced by MRI contrast enhancement in vitro, and selective cytotoxicity against cancer cells while sparing healthy cells. These findings establish Mn-TK and Mn-BR as superior candidates for integrated diagnostic-therapeutic (theranostic) platforms, warranting further in vivo studies to explore their clinical translation. In contrast, Mn-[2]C exhibited lower efficacy and specificity, limiting its potential for therapeutic applications.

### 2.5 Evaluating the Biodistribution, Biosafety, and Therapeutic Efficacy of Metallo-Drugs Mn-TK and Mn-BR in Preclinical Models

#### 2.5.1 Biodistribution in Healthy Animals

The biosafety of Mn-TK and Mn-BR metallo-drugs is crucial for their clinical translation. To assess their systemic distribution and clearance, we performed biodistribution studies in athymic NU/J nude mice using quantitative post-mortem *T*_1_-mapping with a 3.0 T clinical MRI system and ICP-MS analysis to measure manganese concentration per gram of tissue. Mice received a single intraperitoneal (IP) injection of 0.05 mmol-Mn.kg^–1^ body weight, with controls receiving saline. This dosage is double that intended for subsequent toxicity studies. Mn-TK and Mn-BR were well-tolerated, with no observed weight loss or abnormal behavior, indicating minimal in vivo toxicity.^71^ At 4, 24, 72, and 120 h postinjection, mice were euthanized, and post-mortem *T*_1_-maps were obtained before collecting key organs (liver, spleen, kidneys, heart, lungs, and brain) for *ex vivo* ICP-MS analysis.^71^

*T*_1_-mapping MRI analysis showed a rapid reduction in liver and kidney signal intensity postinjection (Figures 4a–c, S25, S26), confirming swift uptake and subsequent clearance of Mn-TK and Mn-BR. Within 4 h, liver signal intensity decreased by 83.4% ± 3.7 for Mn-TK and 84.8% ± 2.8 for Mn-BR, while kidney intensity decreased by 30.8% ± 14.5 for Mn-TK and 26.2% ± 11.4 for Mn-BR. By 24 h, further reductions were observed, with liver signal intensity decreasing to 34.1% ± 8.7 for Mn-TK and 15.5% ± 6.4 for Mn-BR, confirming that hepatic and renal clearance are the primary elimination routes. Over the following days, the signal contrast returned to control levels compared to preinjection images, indicating efficient systemic clearance of Mn-TK and Mn-BR from the mice’s bodies. No MRI signal enhancement was detected in the brain, which aligns with the intended contrast specificity and suggests minimal neurotoxicity.

**Figure 4:**
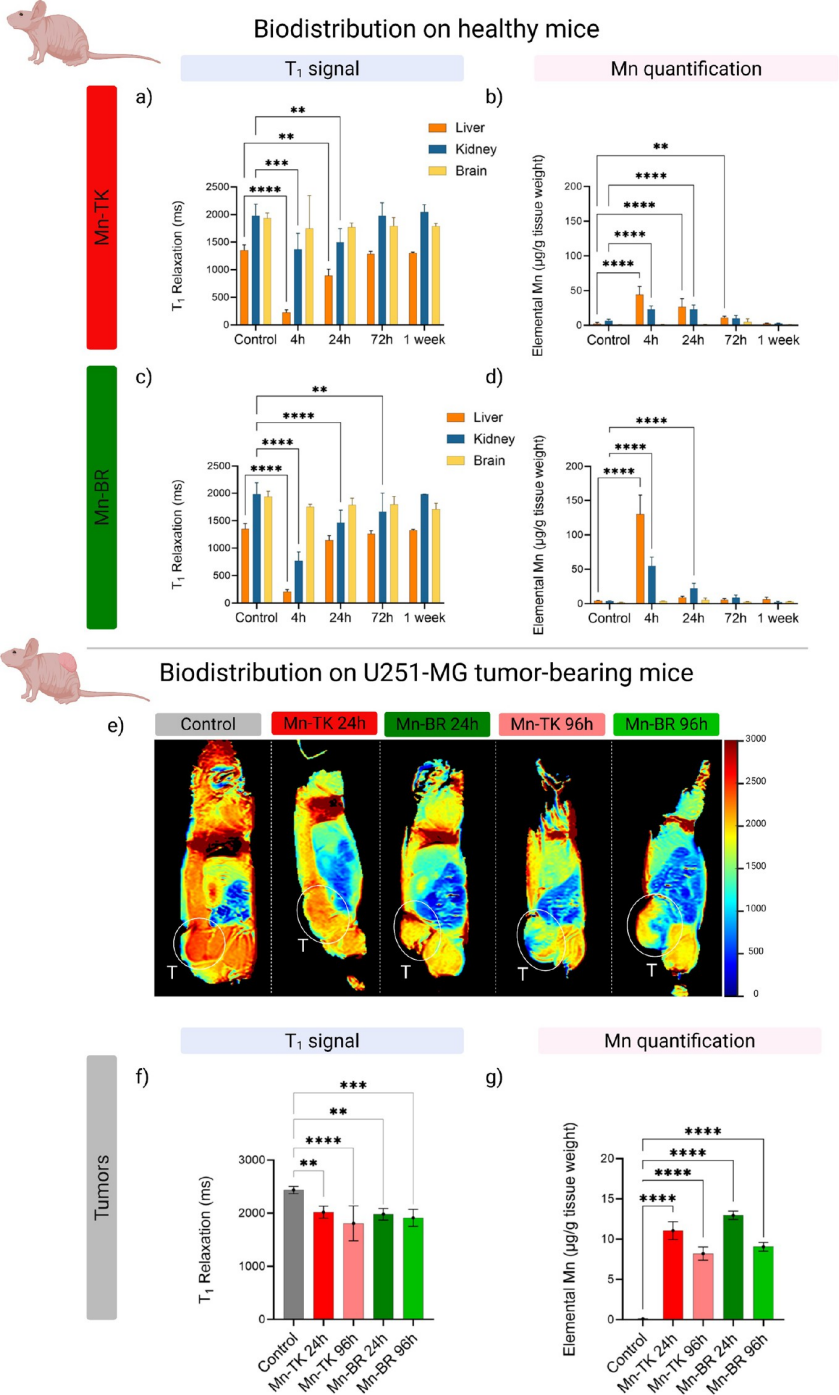
In vivo biodistribution and MRI contrast performance of Mn-TK and Mn-BR in healthy and tumor-bearing mice. (a, c) Changes in *T*_1_ relaxation times in liver, kidney, and brain tissues of mice treated with Mn-TK and Mn-BR, respectively, over 1 week (0.05 mmol-Mn.kg^–1^), compared to control. These graphs illustrate tissue-specific uptake and retention dynamics. (b, d) Manganese levels in the liver, kidney, and brain post-treatment with Mn-TK and Mn-BR, demonstrating the metabolism and clearance patterns. (e) Representative *T*_1_ maps of mice illustrating contrast enhancement in tumors at 24 and 96 h postinjection with Mn-TK and Mn-BR, compared to control (3T MRI). (f) Quantitative *T*_1_ relaxation times in tumor tissues, showing significant contrast enhancement due to Mn structures. (g) Analysis of manganese levels in tumor tissues, indicating bioaccumulation of Mn-TK and Mn-BR, which correlates with enhanced MRI contrast.

Furthermore, ICP-MS analysis provided a detailed quantitative measure of Mn uptake in various organs (Figures 4b–d and S28). Four hours after injection, manganese levels in the liver showed a dramatic increase, rising 20-fold for Mn-TK and 32-fold for Mn-BR compared to controls. This surge decreased by 24 h and returned to near-normal levels by 72 h. This pattern of rapid peak and decline supports efficient hepatic processing and clearance. Kidney analysis revealed peak manganese levels at 24 h, which decreased by half by 72 h, suggesting active renal clearance.

These findings suggest that Mn-TK and Mn-BR exhibit rapid clearance profiles (within 3 days), making them viable candidates for clinical applications with minimal long-term toxicity. In contrast, gadolinium-based contrast agents (GBCAs) are known to persist in tissues, including the brain, raising safety concerns.^5^ These metallo-drugs offer a safer alternative with improved excretion pathways, supporting their potential for clinical translation.

#### 2.5.2 Biosafety: Acute Toxicity, Blood Biochemistry, and Histopathology

Acute biosafety of Mn-TK and Mn-BR was evaluated in healthy CD-1 mice following a single intravenous administration of 0.10 mmol-Mn.kg^–1^, exceeding both imaging and therapeutic doses. Animals were monitored for 7 days, during which no mortality, behavioral abnormalities, body-weight loss, or injection-site reactions were observed (Figure S29). At the 7-day endpoint, blood biochemical parameters assessing liver (ALT, AST, ALP, bilirubin, albumin, total protein) and renal (urea, creatinine) function were all within normal physiological ranges and comparable to control animals (Figure S30 and Table S9). Histopathological examination of major organs (heart, liver, kidney, and spleen) revealed preserved tissue architecture and normal cellular morphology, with no evidence of inflammation, necrosis, or tissue damage (Figure S31). Collectively, these results demonstrate that Mn-TK and Mn-BR do not induce acute systemic toxicity in vivo, even at supratherapeutic doses, supporting their favorable safety profile and translational potential as theranostic agents.

#### 2.5.3 Tumor Selectivity In Vivo

MRI-guided chemotherapy combines diagnosis and treatment within a single platform, enabling real-time tracking of metallodrug accumulation at tumor sites. To evaluate whether the Mn-based nontrivial structures selectively target tumors in vivo, we used a subcutaneous U251-MG glioblastoma xenograft model in NU/J nude mice. Tumor-bearing animals (tumor volume 50–100 mm^3^) received a single intraperitoneal (IP) injection of either Mn-TK or Mn-BR at a dose of 0.05 mmol-Mn.kg^–1^ body weight. Biodistribution was evaluated at 0, 24, and 96 h postinjection using two independent methods: quantitative *T*_1_ mapping by MRI and elemental manganese quantification by ICP-MS across tumors and major organs, including liver, kidney, spleen, lung, heart, and brain.

MRI analysis showed clear tumor selectivity for both compounds (Figure 4e,f). In untreated control mice, tumor *T*_1_ values averaged ∼ 2435 ms. Following administration of Mn-TK and Mn-BR, *T*_1_ values in the tumor tissue decreased significantly (Table S10). For Mn-TK, *T*_1_ was shortened to 2019 ms at 24 h and 1807 ms at 96 h. For Mn–Br, values decreased to 1980 ms at 24 h and 1912 ms at 96 h. These reductions correspond to a substantial increase in relaxation rates (1/*T*_1_) and yielded bright, persistent tumor contrast up to 96 h (Figure 4e).

Importantly, off-target tissues showed minimal signal change. Brain exhibited less than 5% variation in *T*_1_ values across all time points, indicating negligible nonspecific uptake. Transient signal changes were observed in the liver and kidney, consistent with hepatic and renal clearance pathways, but *T*_1_ values in these organs returned toward baseline by 96 h. Notably, the tumor-to-liver contrast ratio improved progressively over time, a clinically valuable parameter that remains difficult to achieve with conventional manganese or gadolinium-based chelates.^28,43^ This increasing ratio enhances tumor visibility and is especially important for early stage detection and treatment monitoring. Together, these in vivo results confirm that Mn-TK and Mn-BR accumulate selectively and persistently in tumors, with minimal off-target distribution, making them strong candidates for MRI-guided chemotherapy.

ICP-MS quantification strongly corroborated the MRI results. Elemental analysis revealed a dramatic increase in manganese accumulation within tumor tissues following treatment. In control animals, baseline Mn levels were approximately 0.09 μg.g^–1^. At 24 h postinjection, tumor manganese concentrations rose to 11.1 μg.g^–1^ for Mn-TK and 13.0 μg/g for Mn-BR, corresponding to more than 100-fold enrichment compared to untreated controls (Figure 4g, Table S11). By 96 h, Mn levels in the tumors remained elevated at 8.2 μg g^–1^ for Mn-TK and 9.1 μg.g^–1^ for Mn-BR, demonstrating sustained retention at the tumor site. In contrast, manganese concentrations in clearance organs such as the liver and kidneys declined by more than 60% over the same period, confirming efficient systemic clearance. Importantly, Mn levels in the brain, heart, and lungs remained near baseline throughout the study, indicating the absence of off-target accumulation in critical organs and ruling out potential neurotoxicity or cardiotoxicity. The spleen exhibited only modest and transient manganese uptake, consistent with its role in the reticuloendothelial system.

The excellent concordance between *T*_1_-weighted MRI signal enhancement and ICP-MS–based elemental quantification provides two independent yet converging lines of evidence for the selective and sustained tumor accumulation of Mn-TK and Mn-BR.

#### 2.5.4 Comparative Analysis of In Vivo Antitumor Efficacy

To assess the anticancer effects of Mn-TK and Mn-BR, U251-MG tumor-bearing nude mice (tumor volume: 50–100 mm^3^) were treated with 0.025 mmol-Mn.kg^–1^ IP injections every 2 days for 13 days (Figure 5a). Mn-TK and Mn-BR significantly inhibited tumor growth compared to the control group injected with saline (Figure 5b). Statistical analysis confirmed that the tumor growth inhibition by Mn-TK and Mn-BR was highly significant (*p* < 0.0001), highlighting the efficacy of these treatments. Additionally, no significant weight loss was observed in any treatment groups, confirming good tolerability (Figure 5c) and minimal systemic side effects.

**Figure 5:**
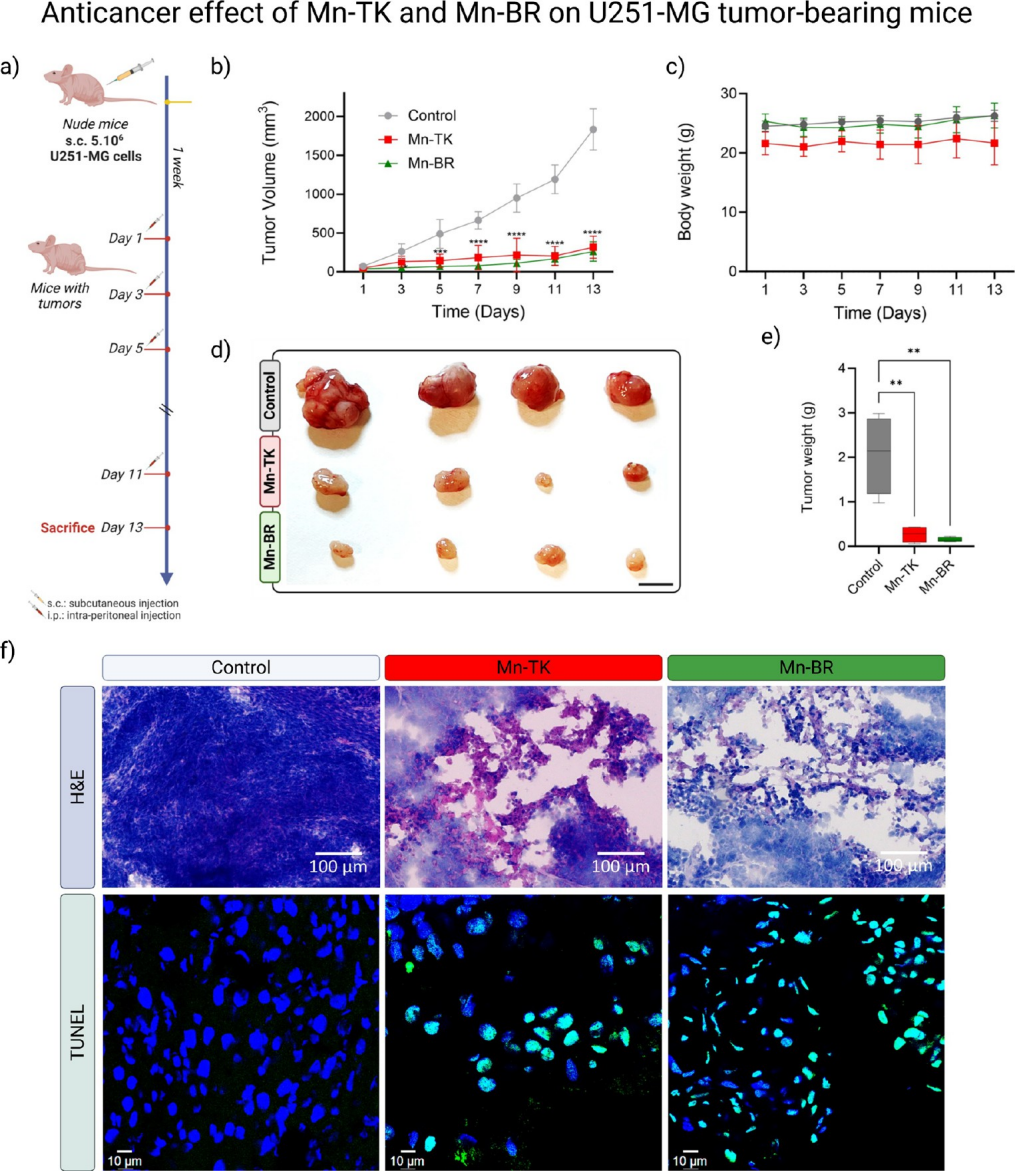
In vivo antitumor efficacy of Mn-TK and Mn-BR in a U251-MG xenograft model. (a) Experimental timeline: Treatment of nude mice implanted subcutaneously with U251-MG cancer cells, detailing the dosing schedule (0.025 mmol-Mn.kg^–1^) and duration of the study. (b) Graph depicting tumor volume progression over 13 days, highlighting significant tumor growth inhibition in mice treated with Mn-TK and Mn-BR compared to controls. (c) Body weight measurements throughout treatment, indicating the treatment’s nontoxic nature. (d) Visual comparison of excised tumors at the end of the study, showing marked reductions in tumor size in Mn-TK and Mn-BR treated groups. (e) Box plot of final tumor weights across treatment groups, with statistical analysis showing a substantial reduction in tumor weight in the Mn-TK and Mn-BR groups compared to controls. (f) Histological and apoptotic analysis of tumor tissues. Representative H&E-stained tumor sections reveal dense, highly cellular tumor architecture in control mice, whereas Mn-TK- and Mn-BR-treated tumors exhibit pronounced structural disruption and reduced cellularity. Corresponding TUNEL staining demonstrates minimal apoptosis in control tumors and markedly increased apoptotic cell death in Mn-TK- and Mn-BR-treated tumors, with the strongest apoptotic response observed in the Mn-BR group. This figure highlights the potent antitumor activity and minimal systemic toxicity of Mn-TK and Mn-BR, affirming their efficacy as theranostic agents for cancer treatment. Statistical significance is denoted by asterisks: *p < 0.05, **p < 0.01, ***p < 0.001, and ****p < 0.0001.

At the end of the treatment period, the mice were euthanized, and the tumors were excised for detailed analysis. Comparative assessments of tumor size and weight showed significant differences between the treated and control groups (Figure 5d). The Mn-TK-treated group showed an 87% (*p* < 0.001) decrease in average tumor weight, while the Mn-BR-treated group showed an even more significant 93% reduction (*p* < 0.001) relative to the control (Figure 5e). These results underscore the potent therapeutic efficacy of Mn-TK and Mn-BR in this cancer model, confirming their significant potential as effective therapeutics in cancer treatment and imaging.

To elucidate the cellular basis of tumor growth inhibition, excised tumors were further analyzed by hematoxylin and eosin (H&E) and TUNEL staining (Figure 5f). Tumors from control mice displayed dense cellular architecture with preserved nuclear morphology, consistent with rapid and aggressive tumor growth. In contrast, Mn-TK–treated tumors exhibited disrupted tissue organization, reduced cellular density, and frequent nuclear condensation and fragmentation, indicative of apoptotic cell death. Mn-BR–treated tumors showed the most severe histopathological alterations, including extensive architectural collapse and widespread nuclear fragmentation.

Apoptosis was directly confirmed by TUNEL staining. Control tumors showed negligible TUNEL-positive nuclei, whereas Mn-TK treatment induced a substantial increase in apoptotic cells distributed throughout the tumor tissue. Mn-BR–treated tumors exhibited the highest density and spatial extent of TUNEL-positive nuclei, reflecting a robust and widespread apoptotic response. Importantly, the degree of TUNEL positivity closely correlated with tumor growth inhibition and final tumor weight reduction, indicating that Mn-TK and Mn-BR suppress tumor progression predominantly through apoptosis rather than nonspecific necrosis.

Collectively, these results demonstrate that Mn-TK and Mn-BR exert potent in vivo antitumor activity with excellent tolerability. The more pronounced apoptotic response observed for Mn-BR is consistent with its higher manganese loading, enhanced intratumoral accumulation, and superior therapeutic performance. These findings establish a clear mechanistic link between molecular topology, efficient manganese delivery to tumors, and apoptosis-driven tumor suppression in vivo.

### 2.6 Enhancing MRI-Guided Chemotherapy with Metal–Organic Structures Mn-TK and Mn-BR in a Spontaneous Glioblastoma (GBM) Mouse Model

The efficacy of Mn-TK and Mn-BR in enhancing the precision of MRI-guided chemotherapy for aggressive brain tumors was evaluated using a spontaneous glioblastoma (GBM) mouse model. This model was generated through the deletion of the Ink4a/Arf and Pten genes and expression of heparin-binding epidermal growth factor-like growth factor (HBEGF), a ligand for the epidermal growth factor receptor (EGFR). These genetic modifications recapitulate the molecular signature, growth kinetics, and infiltrative behavior of the classical human GBM subtype, providing a clinically relevant system that preserves an intact blood–brain barrier (BBB) and a partially disrupted blood–tumor barrier (BTB). The tumor-initiating role of HBEGF in this context has been previously validated by Shin et al.^72^ This stringent model thus serves as an ideal platform for evaluating whether the Mn-based topological complexes can overcome physiological barriers and achieve precise, tumor-confined MRI enhancement.

A clinical gadolinium-based contrast agent (Multihance) was administered 4 days prior to manganese dosing to serve as an anatomical localizer (Figure 6). Consistent with the short blood half-life and low retention of Gd chelates in advanced GBM, the 4-h post-Gd scans displayed only faint, heterogeneous enhancement within necrotic and perivascular regions.^72^ These images were used solely as spatial references for subsequent manganese scans, not as benchmarks of efficacy. After a 4-day washout, administration of MnCl_2_ produced diffuse, nonselective hyperintensity throughout the brain, with poor lesion demarcation and a low tumor-to-contralateral ratio, reflecting passive distribution of free Mn^2+^ (Figure S38). In contrast, a subsequent 4-day washout followed by administration of Mn-TK or Mn-BR (0.05 mmol-Mn.kg^–1^, i.p.) resulted in robust and sharply defined *T*_1_-weighted enhancement confined exclusively to the tumor region as early as 4 h postinjection (Figure 6b–e), with minimal background signal. Notably, tumor-to-contralateral signal ratios increased by 136.6% ± 13.8 for Mn-TK and 141.1% ± 13.4 for Mn-BR, significantly surpassing the performance of MnCl_2_ and Gd-based agents under identical conditions. The regions of Mn-TK and Mn-BR enhancement precisely colocalized with tumor boundaries confirmed by H&E staining (Figures 6, S39), demonstrating excellent anatomical fidelity and diagnostic precision. In addition, no enhancement was observed in healthy brains after Mn-BR administration, confirming that BBB penetration and MRI signal amplification occurred only in the presence of tumor-associated vascular disruption.

**Figure 6:**
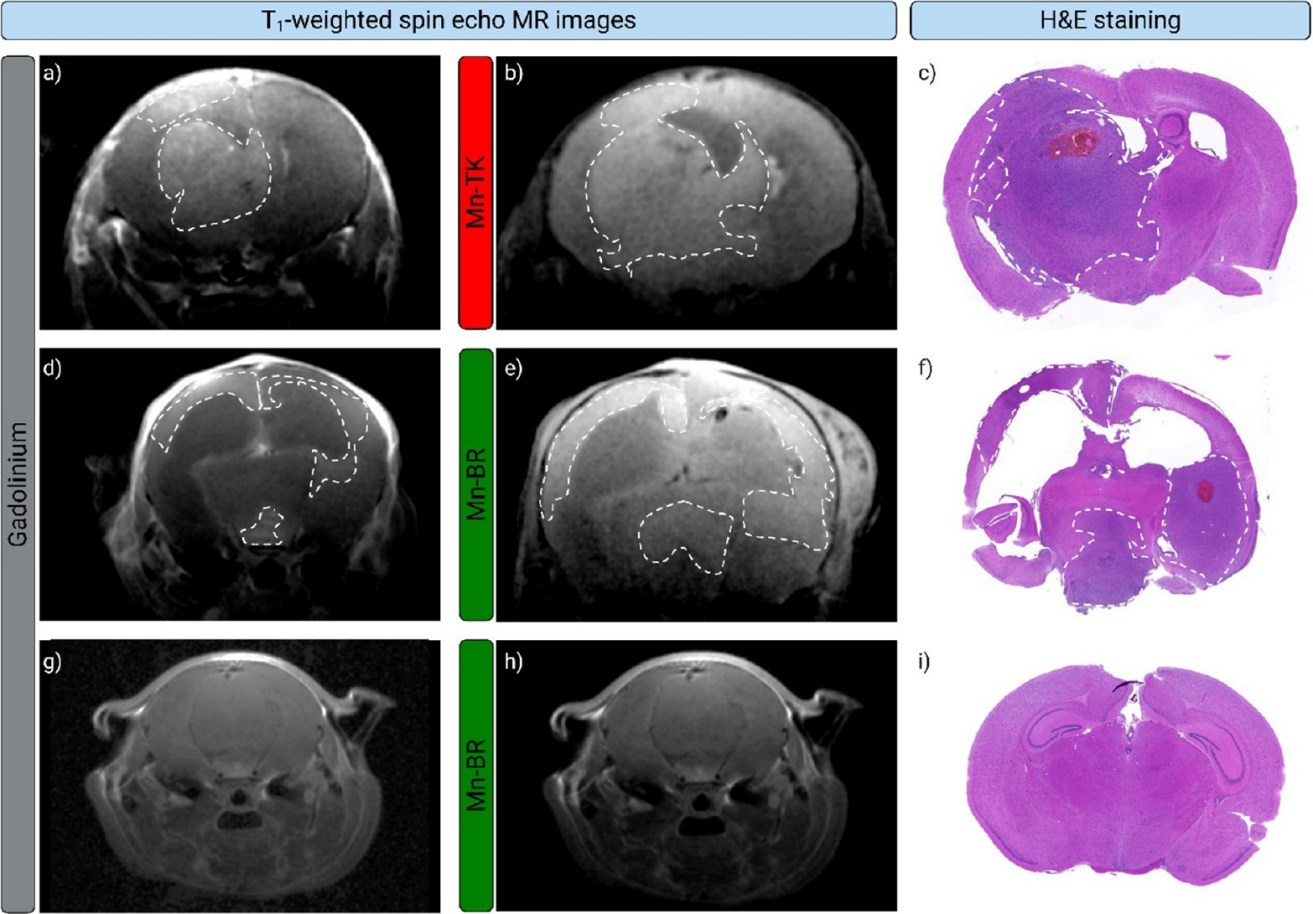
*T*_1_-weighted spin–echo MRI and histological confirmation of tumor-specific enhancement in a spontaneous glioblastoma (GBM) model. Each row represents data acquired from the same animal, first imaged with gadolinium as an anatomical localizer, then with the Mn-based structures after a 4-day washout period, followed by terminal H&E staining. Row a–c (Mn-TK): (a) Gadolinium localizer (Multihance, 0.1 mmol-Gd.kg^–1^, imaged 4 h postinjection); (b) Mn-TK (0.05 mmol-Mn.kg^–1^, i.p., imaged 4 h postdose); (c) corresponding H&E section. White dashed lines indicate tumor boundaries. Gadolinium provides faint, heterogeneous delineation, whereas Mn-TK yields strong, sharply defined enhancement that matches histology. Row d–f (Mn-BR): (d) Gadolinium localizer acquired 4 h post-Gd; (e) Mn-BR (0.05 mmol-Mn.kg^–1^, i.p., imaged 4 h postdose); (f) H&E section showing excellent colocalization between MRI contrast and tumor margins, confirming robust Mn-BR enhancement. Row g–i (Specificity control, no tumor): (g) Gadolinium and (h) Mn-BR scans show no focal signal enhancement; (i) H&E verifies the absence of tumor tissue. Gadolinium imaging was always performed 4 days prior to manganese administration to ensure complete washout. This within-subject design highlights the superior, tumor-restricted contrast achieved with Mn-TK and Mn-BR relative to Gd-based agents.

To further investigate the mechanism underlying their ability to traverse biological barriers, in vitro permeability assays were conducted using confluent bEnd.3 murine brain endothelial monolayers grown on Transwell inserts. After 4 h of incubation, 21.06 ± 2.8% of Mn-TK and 16.00 ± 0.6% of Mn-BR were detected in the basolateral chamber, confirming their efficient passage across the endothelial layer under physiological conditions. These data indicate that both compounds can cross the blood–brain barrier (BBB) through an active, energy-dependent mechanism rather than passive diffusion.

Mechanistically, this behavior is best explained by adsorptive-mediated transcytosis (AMT), a vesicular transport process driven by electrostatic interactions between the positively charged surfaces of Mn-TK (+0.4 au) and Mn-BR (+0.6 au) and the negatively charged, lipid-rich luminal membrane of brain endothelial cells. Their moderate lipophilicity (log P ≈ −0.46 to −0.49) further promotes membrane association and vesicle formation, enabling endocytic internalization and subsequent release into the abluminal compartment. The topology-driven surface charge distribution and multimetallic architecture of these structures enhance their affinity for the endothelial surface and stabilize them during vesicular transport, collectively supporting their efficient BBB and blood–tumor barrier (BTB) transcytosis.^5^ In contrast, conventional Gd-based chelates rely primarily on paracellular leakage through compromised tight junctions, resulting in heterogeneous and patchy contrast enhancement within glioblastoma tissue.^72^ This limitation was reflected in our model by a weak, uneven Gd signal at 4 h postinjection. By comparison, the consistent electrostatic profile and nanoscale topology of Mn-TK and Mn-BR enable uniform endothelial interaction and deeper, more homogeneous tumor penetration, leading to markedly improved contrast uniformity across the tumor volume. Collectively, these results demonstrate that Mn-TK and Mn-BR exploit AMT as a dominant and topology-dependent route for crossing the BBB and BTB, thereby overcoming one of the key limitations of traditional small-molecule MRI agents.

Both Mn-TK and Mn-BR exhibited rapid systemic clearance, with most of the administered dose eliminated within approximately 72 h through combined renal and hepatobiliary excretion, thereby minimizing the risk of long-term manganese retention that often limits the clinical use of gadolinium-based agents.^5^ Their biodegradable molecular design allows gradual disassembly under physiological conditions, ensuring both efficient elimination and favorable safety profiles. The unique topological and physicochemical features of these complexes not only facilitate blood–brain and blood–tumor barrier traversal but also enhance MRI sensitivity at clinically relevant doses. Collectively, these properties enable high-fidelity tumor imaging coupled with safe and timely clearance, addressing a long-standing challenge in the development of metal-based theranostic agents.

## 3 Conclusions

This study establishes Manganese-templated nontrivial topological structures, Mn-[2]­C, Mn-TK, and Mn-BR, as a fundamentally new class of theranostic agents that integrate high-precision diagnostics with targeted therapeutic efficacy. Through the deliberate application of molecular topology, these architectures exhibit distinctive physicochemical properties, including multivalent Mn centers, enhanced lipophilicity, and high electrostatic potentials, that collectively govern their stability, relaxivity, and selective biological action.

Among the three structures, Mn-TK and Mn-BR emerge as particularly promising. Their combination of higher positive charge and increased lipophilicity facilitates efficient tumor targeting via adsorptive-mediated transcytosis and membrane affinity, leading to preferential uptake into cancer cells and localization within lipid-rich subcellular compartments. These properties underpin their tumor-selective accumulation, strong and durable MRI contrast enhancement, and pH-responsive cytotoxicity, while minimizing off-target effects in healthy tissues.

Importantly, Mn-TK and Mn-BR outperform established clinical agents such as Gd-DTPA and Mn-DPDP in terms of relaxivity and tumor specificity. They also show rapid systemic clearance and minimal off-target accumulation, addressing long-standing concerns about toxicity and metal retention. Their ability to cross the blood–brain barrier and delineate glioblastoma in vivo highlights their clinical promise for neuro-oncology.

This work introduces a generalizable strategy for designing manganese-based theranostic agents by integrating topological coordination chemistry with tunable lipophilicity and electrostatics. Mn-TK and Mn-BR represent a new generation of multifunctional agents capable of simultaneous imaging, targeted delivery, and therapy, offering a safe and translational approach to precision cancer treatment.

## Supplementary Material



## Notes

General materials and methods; synthesis and characterization of Mn-[2]­C, Mn-TK, and Mn-BR; stability studies in HEPES buffer and under acidic conditions; transmetallation and lipophilicity analyses; 17O NMR relaxivity measurements and comparison with representative Mn-based MRI contrast agents; in vitro MRI studies, ICP-MS uptake, cytotoxicity, hemolysis, kinetic uptake, endocytosis inhibition, subcellular fractionation, TEM, Annexin V/PI apoptosis, live-cell apoptosis imaging, Western blot, LDH release, TUNEL assay, ROS generation, JC-1 mitochondrial membrane potential analysis, Seahorse bioenergetics, and cell proliferation assays; in vivo biodistribution, biosafety, blood biochemistry, histopathology, antitumor efficacy, and tumor apoptosis studies; spontaneous glioblastoma model experiments including MRI, histological analysis, immunohistochemistry, and Transwell permeability assay; statistical analysis; and references (1–29) (PDF)
