# Supplementary material for: Manganese-Templated Nontrivial Structures for MRI and Therapy
Source: J Am Chem Soc. 2026 Apr 1;148(15):15529–49. doi: 10.1021/jacs.5c19016 (PMC13107457; doi:10.1021/jacs.5c19016)
Supplement: Supplementary file 1 [file ja5c19016_si_001.pdf]

Manganese-Templated Non-Trivial Structures for MRI and Therapy

Farah Benyettou,[1‡*] Thirumurugan Prakasam,[1‡] Mostafa Khair,[2‡] Osama Abdullah,[2‡] Matteo Lusi,[3] Haidee Paterson,[2] Maryam Alkaabi,[1] Sneha Thomas,[2] Rainer Straubinger,[2] Nosayba Al Damook,[2] Maylis Boitet,[2] Mamoun Abelbaki,[4] Judalyn Del Monte,[4] Diana Yu,[5] Rick E. Heinz,[5] Sheri L. Holmen,[5] Edward Hsu,[6] Carlos Platas-Iglesias,[7] Gennaro Esposito,[1] Ali Trabolsi[1*]

‡ equal contribution

[1] *Chemistry Program, New York University Abu Dhabi (NYUAD), Abu Dhabi 129188, United Arab Emirates*

[2] *Core Technology Platforms, New York University Abu Dhabi (NYUAD), 129188 Abu Dhabi, United Arab Emirates*

[3] *Department of Chemical Science & Bernal Institute, University of Limerick, Limerick, Republic of Ireland*

[4] *PHRC, New York University Abu Dhabi (NYUAD), Abu Dhabi 129188, United Arab Emirates*

[5] *Department of Surgery, University of Utah Health Sciences Center, Salt Lake City, Utah 84112, USA; Huntsman Cancer Institute, University of Utah Health Sciences Center, Salt Lake City, Utah 84112, USA*

[6] *Department of Biomedical Engineering, University of Utah, Salt Lake City, Utah 84112, USA*

[7] *Centro Interdisciplinar de Química e Bioloxía (CICA) and Departamento de Química, Facultade de Ciencias, Universidade da Coruña, A Coruña 15071, Galicia, Spain*

Contents

1 - General Materials and Methods 4
2. General Synthetic Procedure 6
2.1. Synthesis of Manganese-[2]Catenate (Mn-[2]C) 7
2.2. Synthesis of Manganese-Trefoil Knot (Mn-TK) 7
3. General characterizations 8
3.1. Crystal Structure of Mn-[2]C, Mn-TK and Mn-BR 8
3.2. Mass Spectrometry 9
3.3. Stability Study 10
3.4. Transmetallation Study 13
3.5. Lipophilicity Study 14
4. ^{17}O NMR study. 17
5. MRI Contrast Agent Evaluation 19
6. *In vitro* biological studies 26
6.1. Cell culture 26
6.2. MR Imaging of U251-MG and HEK-293 Cells 26
6.3. Quantification of Manganese 27
6.4. *In vitro* Cell Toxicity Assesment 28
6.5. Hemolysis Assay 32
6.6. Kinetic of Uptake 33
6.7. Endocytic Inhibitor 34
6.8. Subcellular Fractionation of U251-MG Cells: Nuclear, Cytosolic, Cytoplasmic, Mitochondrial, and Membrane Fractions 36
6.9. Intracellular Distribution Study using TEM 37
6.10. Cell Apoptosis Detection Using Annexin V-FITC and Propidium Iodide (PI) Staining 42
6.11. Live-Cell Annexin V Assay 44
6.12. Western Blotting 45
6.13. LDH Release Experiment 46
6.14. DNA Damage Assessed by TUNEL Assay 47
6.15. Intracellular ROS Quantification and Lysosomal pH-dependence (H_2DCFDA ± NH_4Cl) 48
6.16. Mitochondrial Membrane Potential ($\Delta\Psi m$) Alterations 50
6.17. Mitochondrial Bioenergetics Assessment by Seahorse XF96 52

6.18. Cell Growth Analysis 53
7. *In vivo* Biological Studies 55
7.1. Biodistribution Study on Healthy Animals 55
7.1.1. Quantitative T_1-Mapping with Spin echo MR Imaging 55
7.1.2. Manganese Determination in Tissue 58
7.2. In Vivo Biosafety Evaluation 60
7.2.1. Acute Toxicity Study and Body-Weight Monitoring 60
7.2.2. Blood Biochemical Analysis 61
7.2.3. Histopathological Examination (Hematoxylin and Eosin Staining) 64
7.3. Tumor Model Establishment 65
7.4. Biodistribution Study on Tumor-bearing Mice 66
7.5. *In vivo* Antitumor Efficacy 69
7.6. In Vivo Tumor Apoptosis Analysis by TUNEL Staining 70
8. Enhancing MRI-Guided Chemotherapy for Brain Tumors with Metal-Organic Structures Mn-TK and Mn-BR 73
8.1. Spontaneous Glioblastoma (GBM) Mouse Model 73
8.2. MRI Imaging 73
8.3. Histological Analysis 74
8.4. Immunohistochemistry (IHC) 74
8.5. Transwell Permeability Assay 76
9. Statistical Analysis. 77
References 78

1 - General Materials and Methods

All reagents and starting materials were purchased from Sigma-Aldrich and used without further purification. Deionized water was used from Millipore Gradient Milli-Q water purification system. Thin-layer chromatography (TLC) was performed on silica gel 60 F254 (E. Merck). The plates were inspected under UV light. Column chromatography was performed on silica gel 60F (Merck 9385, 0.040–0.063 mm).

Routine nuclear magnetic resonance (NMR) spectra were recorded at 25 °C on a Bruker Advance III spectrometer, with working frequencies of 600 and 500 MHz for ^{1}H, and 151.0 and 125.0 MHz for ^{13}C nuclei. All chemical shifts are reported in ppm relative to the signals corresponding to the residual non-deuterated solvents (CD_3CN: δ= 1.94 ppm, CD_3OD: δ= 3.31 ppm).[1-2] All ^{13}C spectra were recorded with the simultaneous decoupling of proton nuclei. Coupling constant values (*J*) are given in hertz (Hz). The multiplicity of the proton spectrum is abbreviated in the following way: s (singlet), d (doublet), dd (doublet of doublets), t (triplet), q (quartet), qt (quintet), sx (sextet), m (multiplet) and a wide signal is preceded by br (broad). High resolution mass spectrometry (HRMS) analyses were performed using an Agilent 6540 UHA Accurate Mass Q-TOF / LC - MS-spectrometer in the positive mode and an acetonitrile/water used a gradient in C18 column.

UV-visible absorption spectra were recorded with an Agilent Technologies Cary 5000 Series UV-Vis-NIR Spectrophotometer in water at room temperature (298 K). Solutions were examined in 1 cm spectrofluorimetric quartz cells. The experimental error of the wavelength values was estimated to be ~1 nm.

Transmission electron microscopy (TEM) images were obtained using a Thermo Fisher Scientific (TFS) Talos F200X scanning/transmission electron microscope (S/TEM) operating at 200 kV acceleration voltage. Samples for the TEM investigation were prepared by placing a 3µL drop of the NP suspension on a carbon-coated copper grid (TED PELLA, Inc.) and allowing the solvent to evaporate. The high-resolution images of periodic structures were analyzed using Velox software.

The Manganese content was analyzed using the Inductively coupled plasma mass spectrometry (ICP-MS) on the Agilent 7800 series instrument after digesting the compounds in nitric acid. Manganese concentrations were determined by the external calibration method using Manganese calibration standard solutions in the 1–100 ppm range trace metal grade HNO_3. The digested samples were diluted in milliQ water to a final 100 to 200 ppm concentration measurements with no gas and helium mode. The ICP-MS instrument control Agilent Mass Hunter software was used under the following conditions: RF power 1550 W, nebulizer gas flow 1.03 L/min, auxiliary gas flow 0.90 L/min, and plasma gas flow 15 L/min. Data were processed using Agilent Mass Hunter software.

Flow cytometry analyses were performed on Attune NxT Flow cytometer.

2. General Synthetic Procedure

Mn-BR was synthesized according to the previously reported synthetic procedure.[3]

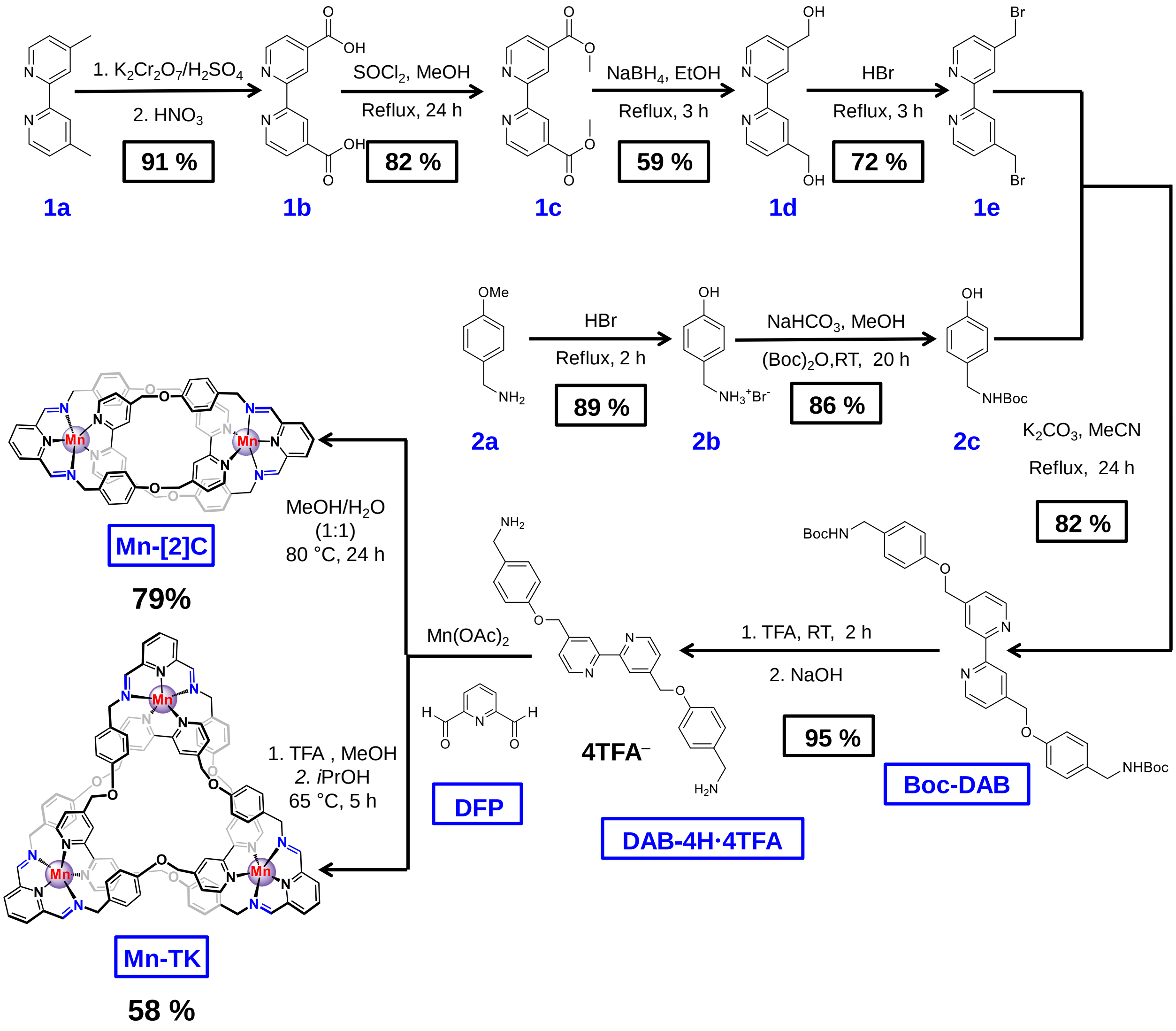

Scheme S1. Synthesis of manganese-[2]Catenate (Mn-[2]C) and manganese-Trefoil Knot (Mn-TK) using a diamino pyridine (DAB), 2,6 diformyl pyridine (DFP), and manganese(II) acetate via an eight-step synthesis.

2.1. Synthesis of Manganese-[2]Catenate (Mn-[2]C)

15.4 mg (0.117 mmol) of 2,6-diformyl pyridine (DFP), 50 mg (0.117 mmol) of neutral DAB, and 22.3 mg (0.129 mmol) of manganese (II) acetate in 10 mL of 1:1 MeOH/H_2O was refluxed at 90°C for 24 hours. After completion of the reaction, the solvent was removed from the reaction mixture, and 10 mL of isopropanol was added to the reaction mixture. The obtained yellow precipitate was filtered, washed with a minimum amount of isopropanol, and dried under vacuum overnight. Manganese [2]catenate was obtained as a pale-yellow solid.

Yield: 64 mg 79%; MS (ESI-HRMS): m/z Calcd for $(C_{70}H_{54}F_6Mn_2N_{10}O_8)^{2+}$: 693.139 [Mn-[2]C•2TFA]$^{2+}$, found: 693.138 [Mn-[2]C•2TFA]$^{2+}$, m/z Calcd for $(C_{68}H_{54}F_3Mn_2N_{10}O_6)^{4+}$: 424.430 [Mn-TK•2TFA]$^{4+}$, found: 424.429 [Mn-TK•2TFA]$^{4+}$.

2.2. Synthesis of Manganese-Trefoil Knot (Mn-TK)

Pale pink solid of freshly Boc-protected DAB·4TFA (0.21 g, 0.24 mmol) was stirred with manganese (II) acetate (0.049 g, 0.28 mmol) and 2,6-diformyl pyridine (DFP) (0.032 g, 0.24 mmol) in 10 mL of isopropanol in a 50 mL round-bottom flask. The reaction was refluxed at 70 °C for 5 hours. The warm solution was filtered, and the precipitate was washed with isopropanol. The precipitate was dried under vacuum for 6 hours and was isolated and identified as the trefoil knot using high-resolution mass spectroscopy.

Yield: 58 % (0.112 mg); MS (ESI-HRMS): m/z Calcd for $(C_{107}H_{81}F_{12}Mn_3N_{15}O_{14})^{2+}$: 1096.201 [Mn-TK•4TFA]$^{2+}$, found: 1096.200 [Mn-TK•4TFA]$^{2+}$, m/z Calcd for $(C_{105}H_{81}F_9Mn_3N_{15}O_{12})^{3+}$: 693.139 [Mn-TK•3TFA]$^{3+}$, found: 693.138 [Mn-TK•3TFA]$^{3+}$, m/z Calcd for $(C_{103}H_{81}F_6Mn_3N_{15}O_{10})^{4+}$: 491.608 [Mn-TK•2TFA]$^{4+}$, found: 491.609 [Mn-TK•2TFA]$^{4+}$.

3. General characterizations

3.1. Crystal Structure of Mn-[2]C, Mn-TK and Mn-BR

Crystal structures were solved from diffraction data collected on a Bruker Quest diffractometer equipped with a microfocus X-ray generator (copper anode, $K\alpha$ = 1.54178 Å) and a Photon detector. Unit cells were identified by the least squares method, and data were integrated using the Bruker Apex III software. Structures were solved by direct methods, and atomic positions were determined based on the electron difference map using SHELX via the X-SEED GUI, by refining the structure against the observed F^2. All heavy atoms were treated anisotropically, with the exception of hydrogen atoms, which were positioned using the riding model based on chemical knowledge and treated isotropically. In Mn-[2]C, the residual electron density was removed in PLATON using the SQUEEZE function.

The single crystal X-ray crystallographic structures of Mn-[2]C, Mn-TK, and Mn-BR are accessible through the Cambridge Crystallography Data Centre (CCDC), with deposition numbers: CCDC **2392920-2392922**. All crystallographic data are available free of charge from the Cambridge Crystallographic Data Centre via www.ccdc.cam.ac.uk/data_request/cif.

3.2. Mass Spectrometry

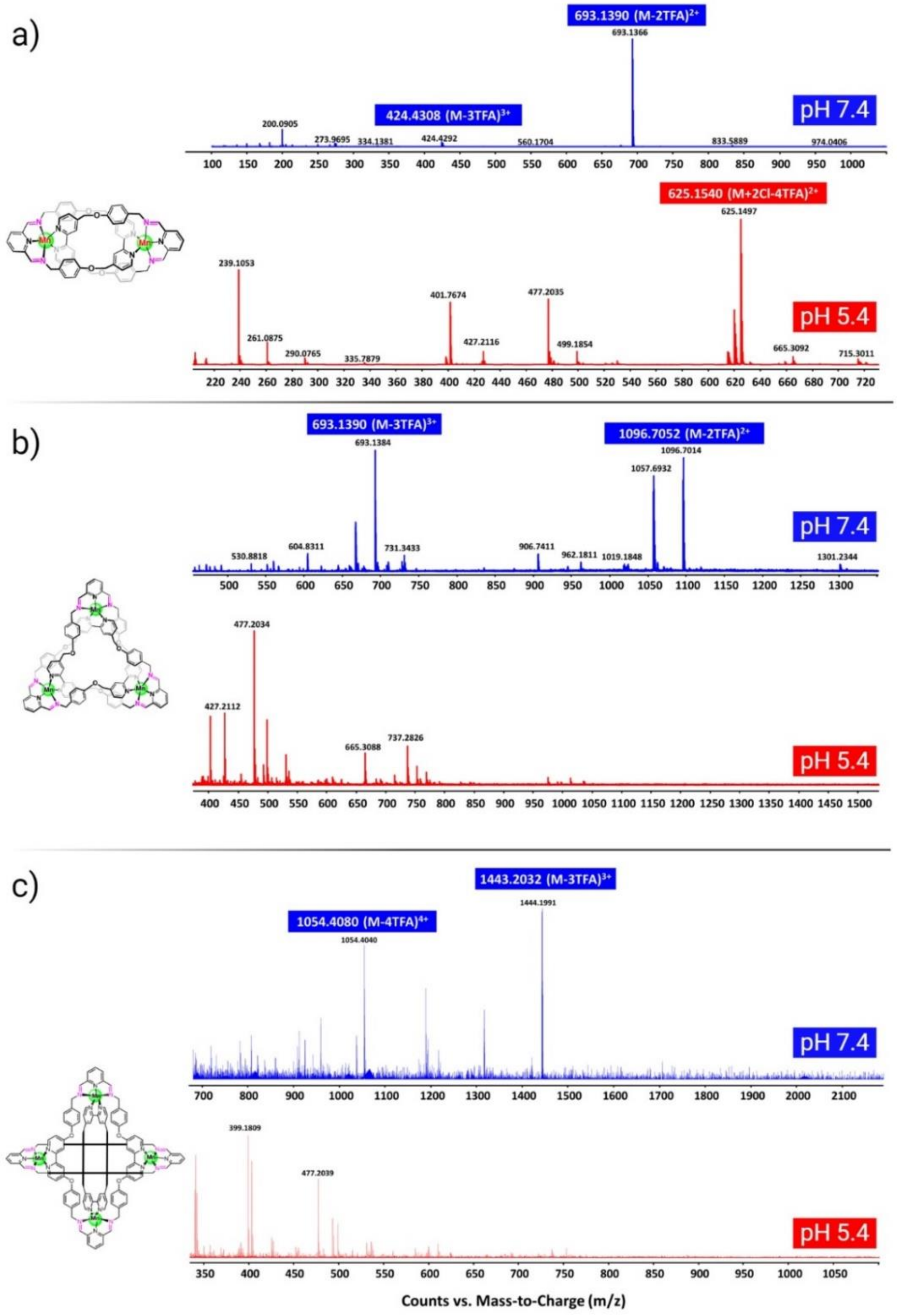

Figure S1. Mass spectrometry of a) Mn-[2]C, b) Mn-TK, and c) Mn-BR suspended 24 hours at pH 7.4 (blue) and 5.4 (red) in HEPES buffer (100 mM).

3.3. Stability Study

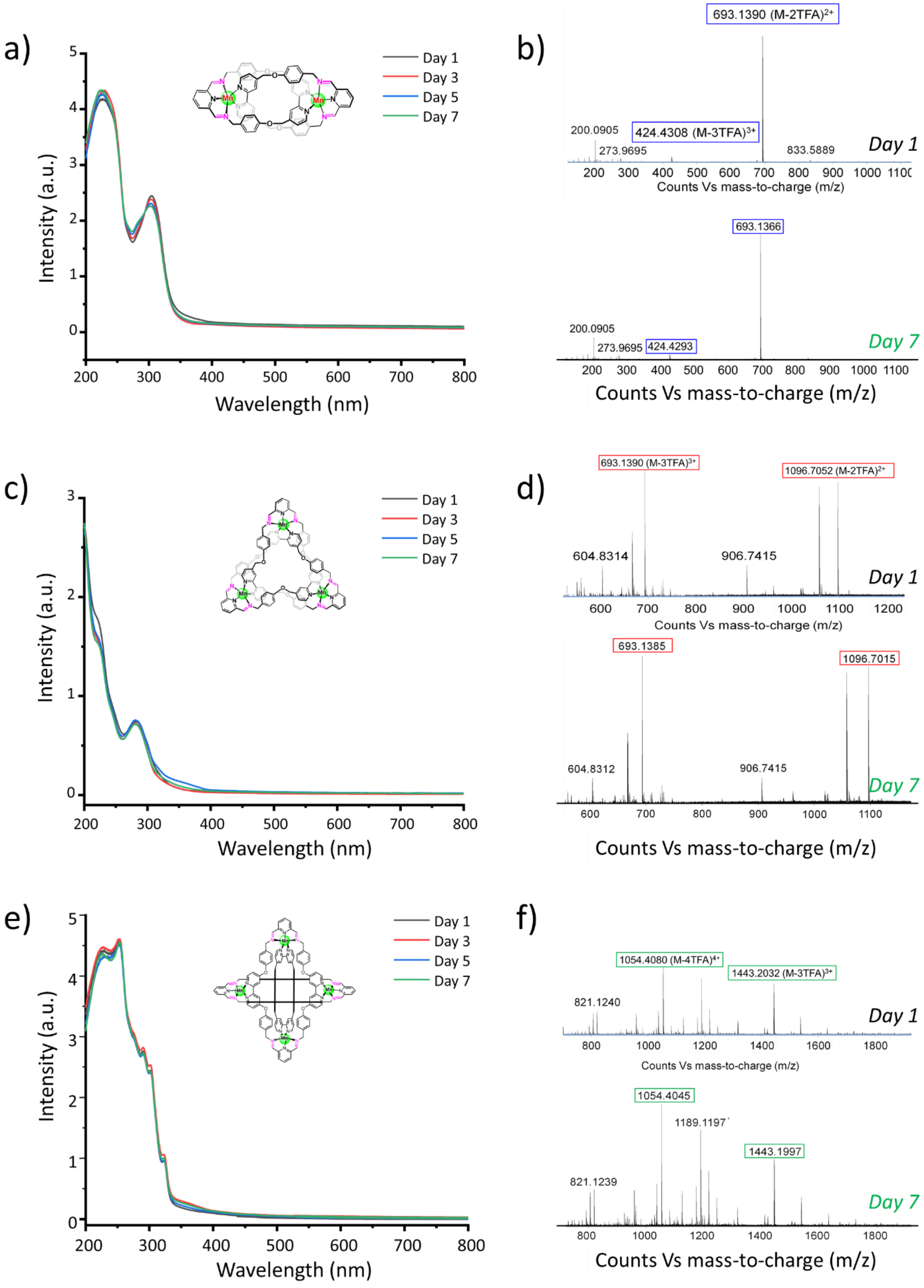

Figure S2. One week stability of Mn-base compounds in HEPES buffer (pH 7.4, 25 °C). UV-Vis absorption spectra of Mn-[2]C (a), Mn-TK (c), and Mn-BR (e) were recorded at days 1, 3, 5, and 7, showing no significant changes in peak position or intensity, indicating stable coordination environments over one week. Complementary high-resolution ESI-MS analysis performed on Day 1 and Day 7 for Mn-[2]C (b), Mn-TK (d), and Mn-BR (f) confirmed the preservation of characteristic isotopic patterns and exact m/z values without degradation products or peak shifts.

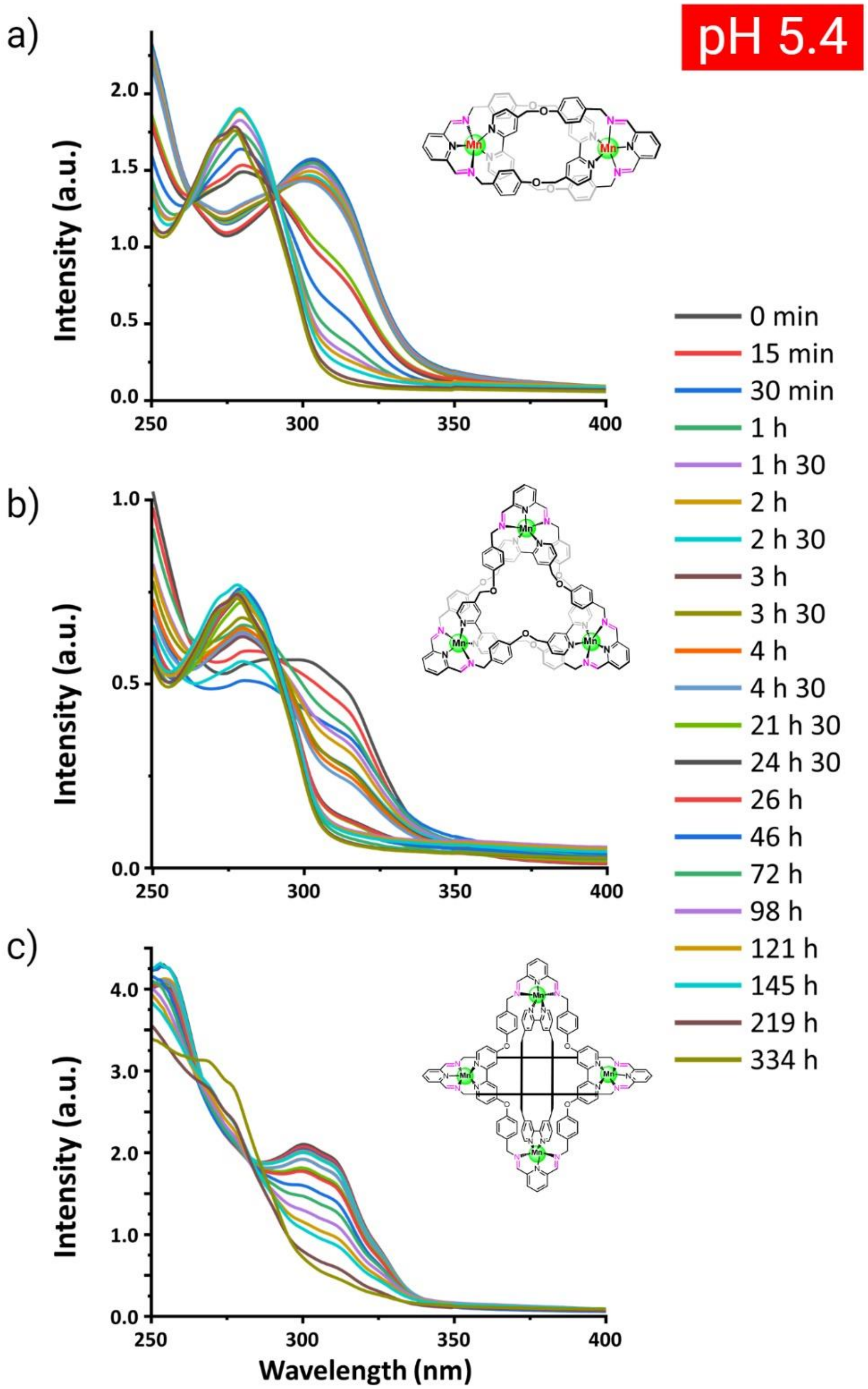

Figure S3. *In vitro* stability of a) Mn-[2]C, b) Mn-TK, and c) Mn-BR at pH 5.4 in HEPES buffer (100 mM) studied using UV-Vis spectroscopy at room temperature over 334 hours, [Mn] = 0.3 M.

3.4. Transmetallation Study

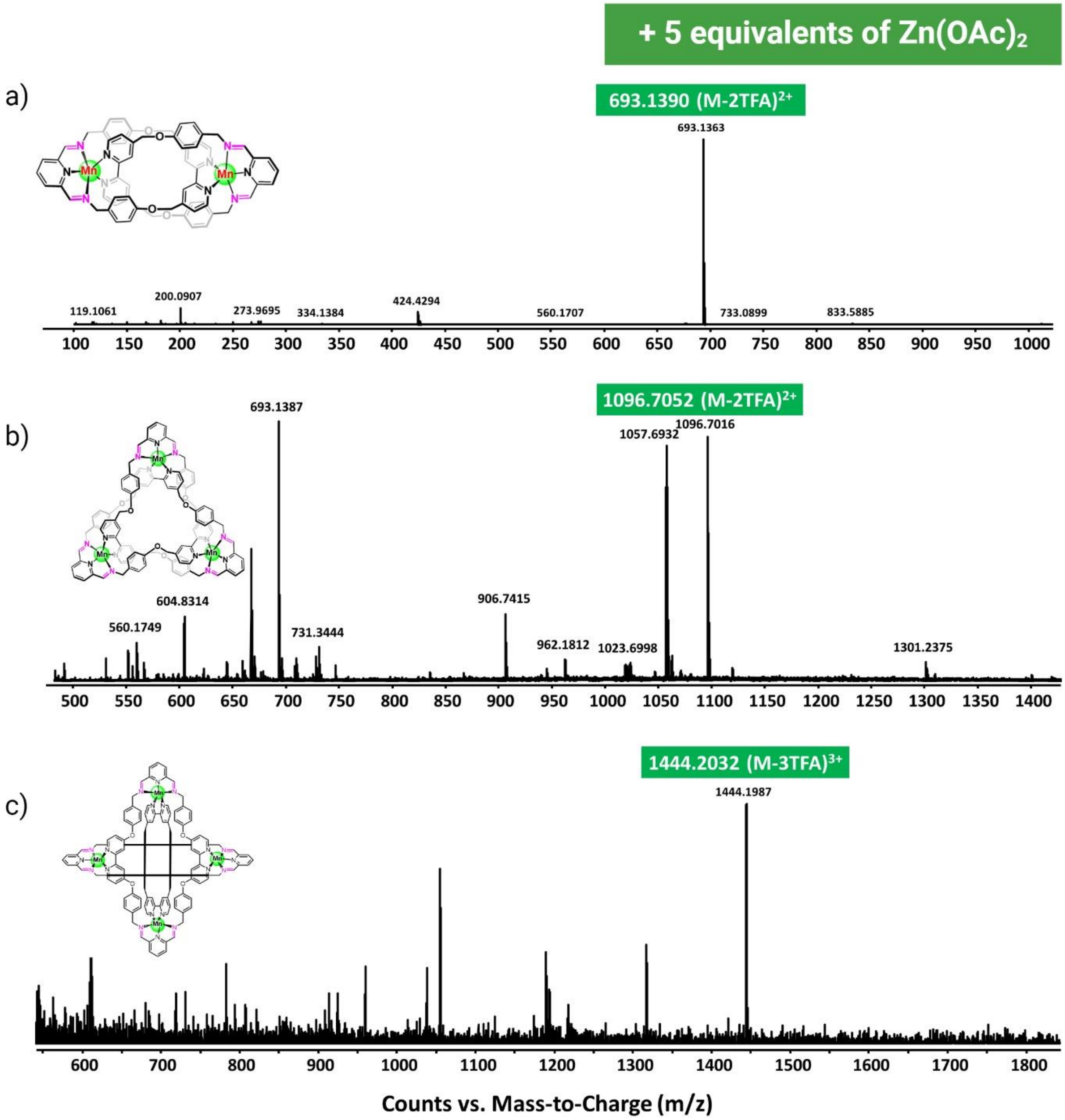

Figure S4. Mass spectrometry of a) Mn-[2]C, b) Mn-TK, and c) Mn-BR suspended for 24 hours at pH 7.4 in the presence of 5 equivalents of $ZnCl_2$ in HEPES buffer (100 mM).

3.5. Lipophilicity Study

The lipophilicity of manganese-based knots and links was assessed using UV-Vis spectroscopy by monitoring changes in their electronic absorption spectra upon interaction with solvents of varying polarity. The shake-flask method was employed to determine the partition coefficient (logP), a critical parameter for evaluating lipophilicity. This method involves equilibrating the manganese complexes between n-octanol and water phases, followed by quantification of their concentrations in each phase using UV-Vis spectroscopy.

Specifically, 4 mL of a 0.05 M solution of the manganese knots and links was vigorously shaken with 4 mL of n-octanol for 2 to 3 hours to allow phase partitioning. Subsequently, the concentration of the complexes in both the n-octanol and aqueous layers was measured. The partition coefficient (P) was calculated as the ratio of the concentration of the complex in the n-octanol phase to that in the aqueous phase, according to the equation:

$$\log P = \log \frac{[\mathrm{MnKL}]_{\mathrm{octanol}}}{[\mathrm{MnKL}]_{\mathrm{water}}}.$$

Using UV-Vis data, the concentrations of manganese knots and links in each phase were determined. The calculated logP values were -0.795, -0.463, and -0.491 for Mn-[2]C, Mn-TK, and Mn-BR, respectively.

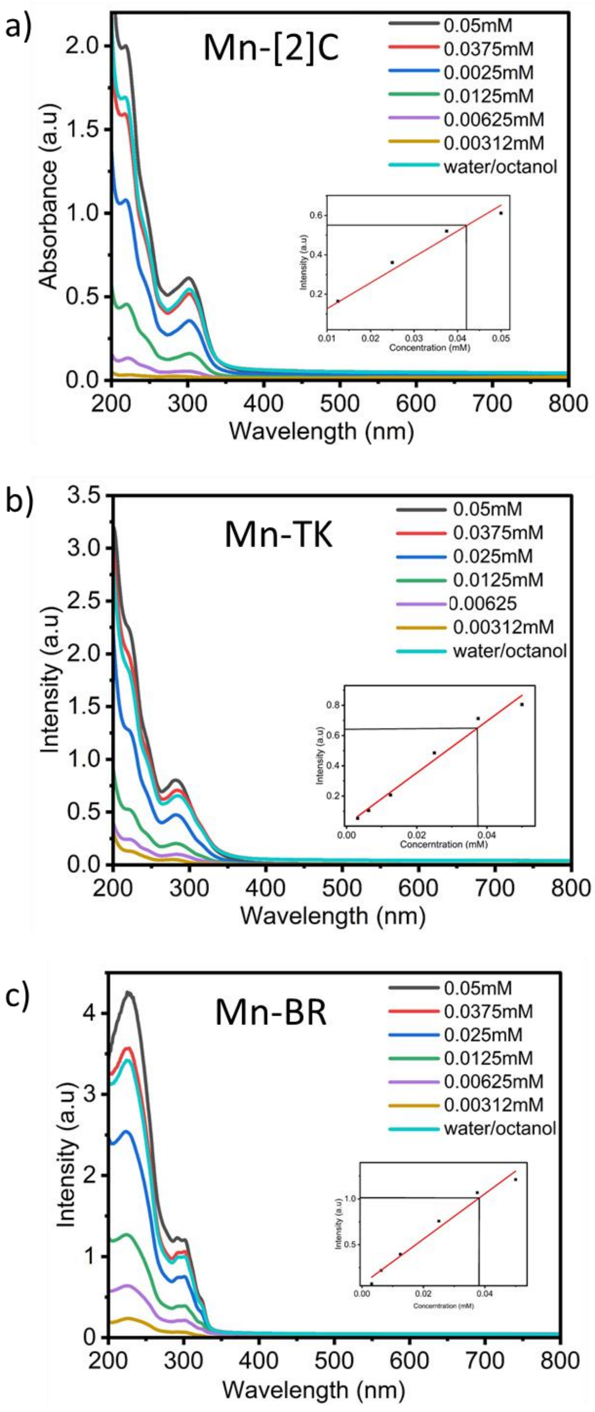

Figure S5. Lipophilicity assessment of Mn-based topological structures using the shake-flask n-octanol/water partitioning method. Representative UV–Vis absorption spectra of a) Mn-[2]C, b) Mn-TK, and c) Mn-BR recorded at different concentrations in n-octanol and water phases. Insets show calibration curves used to calculate the partition coefficients (logP) from the linear correlation between absorbance intensity and concentration.

Table S1. Comparative lipophilicity (logP) values of Mn-based MRI contrast agents, including this work

	log P	Reference
Mn-[2]C	–0.795	This work
Mn-TK	–0.463	This work
Mn-BR	–0.491	This work
Mn-PyC3A	0.575	*J Med Chem* **2018,** *61* (19), 8811-8824[4]
Mn-PyC3A-3-OBn	1.15	*J Med Chem* **2018,** *61* (19), 8811-8824[4]
Mn-EDTA	–2.72	*J. Med. Chem.* **2017***,* 60, 7, 2993–3001[5]
Mn-EDTA-BTA	–1.84	*J. Med. Chem.* **2017**, 60, 7, 2993–3001[5]
MnDPDP	–3.07	*J. Med. Chem.* **2017**, 60, 7, 2993–3001[5]
Mn-BnO-TyrEDTA	0.18	*ACS Applied Bio Materials* **2024,** *7* (3), 1831-1841[6]
Mn-NOTA-NP	–0.93	*Pharmaceuticals* **2023,** *16* (4), 602[7]
Mn-Dendrimer	–3.27	*Bioconjugate Chemistry* **2009,** *20* (4), 760-767[8]

4. ^{17}O NMR study.

The variation of the water transverse relaxation rate as a function of Mn^{2+} concentration was determined from ^{17}O line-widths. Mn^{2+} was introduced in solution as trifluoroacetate salt complexes of the different molecular knots and links or as chloride salt for control. The considered molecular knot and links – [2]Catenate (Mn-[2]C), Trefoil Knot (Mn-TK) and Borromean Ring (Mn-BR), coordinating two, three and six Mn^{2+} ions, respectively – were dissolved at concentrations varying between 0.100 and 0.400 mM due to solubility limits (especially for Mn-[2]C). $MnCl_2$ concentrations were 0.10 and 0.070 mM. ^{17}O-enriched water (0.20% w/w) was employed as solvent according to previously reported protocols.[9] The solvent also contained D_2O (5.5% w/w) for frequency lock. ^{17}O NMR spectra were obtained with a Bruker Avance 500 instrument at 11.7 T, where ^{17}O resonates at 67.79 MHz. The data were acquired on samples in 5 mm tubes unspun, by collecting 512-768 scans over a sweep with of 80 ppm digitized with 4K points (1K = 2^{10}), corresponding to a resolution of 2.6 Hz. An inter-scan relaxation delay of 0.5 s was allowed. The measurement temperature was varied between 274.7 K and 314.5 K to avoid freezing as well as thermal degradation of the metal-organic constructs. The control measurements with $MnCl_2$ were instead carried out over the range 298.0-327.1 K. A thermal equilibration interval of ~15 minutes was always allowed for changes of 2-4 K, under the control of an automatic acquisition routine for variable temperature determinations. The actual temperature values were assessed by CD_3OD calibration.[10] Prior to Fourier transformation, the spectra were apodised by exponential multiplication with line broadening (LB) of 5 Hz and zero-filled to 32K, corresponding to a digital resolution of 0.16 Hz. Half-height line widths ($\Delta\nu_{½}$) of ^{17}O spectra were measured using the dedicated tool of TopSpin 4.0.6 processing software (Bruker) to extract the transverse relaxation rates (R_2) defined as: $R_2 = \pi \times (\Delta\nu_{½} - LB)$.[11]

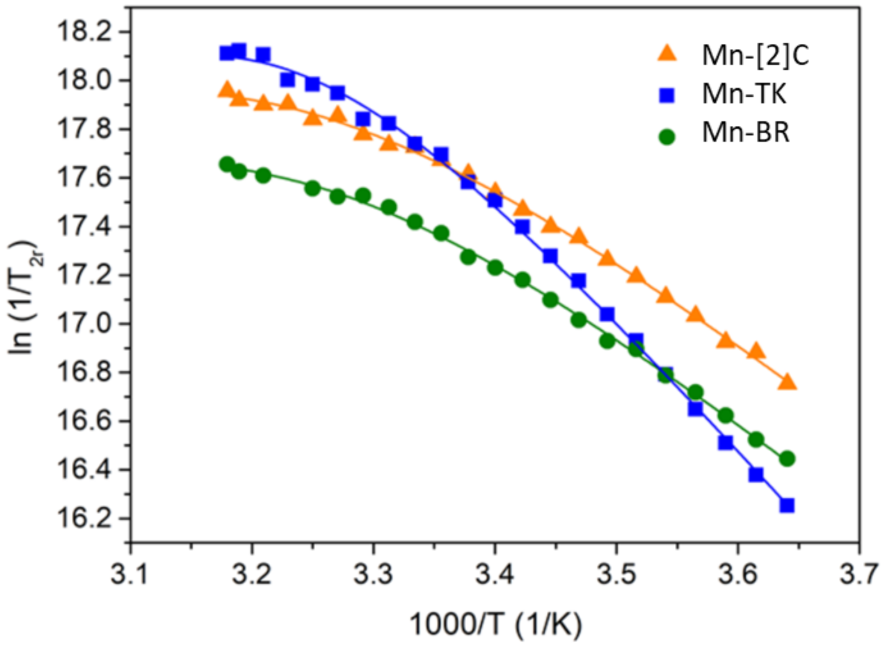

Figure S6. Reduced transverse relaxation rates calculated from the experimental data for Mn-[2]C (orange), Mn-TK (blue) and Mn-BR (green) at 11.7 T. The continuous lines correspond to the fits of the data as explained in the text.

5. MRI Contrast Agent Evaluation

Samples were placed in a transmit/receive quadrature knee coil within a clinical 3T MRI scanner (MAGNETOM Prisma, Siemens Healthineers, Erlangen, Germany) for quantitative T_1 relaxation mapping. A saturation recovery spin echo sequence was employed with the following parameters: variable repetition times (TR, in ms) of 150, 200, 300, 500, 800, 1000, 1500, 3000, 4000, and 8000 ms; echo time (TE) of 11 ms; field of view (FOV) of 130 mm × 130 mm; acquisition matrix of 320 × 320; and slice thickness of 3.5 mm.

The acquired data was processed using MATLAB 2024a (MathWorks Inc.) with a custom script for pixel-by-pixel nonlinear least squares fitting (utilizing the lsqcurvefit function). The signal at each voxel was plotted against its corresponding TR, and a T_1 recovery curve was fit using the $M(TR) = M_0\left(1 - e^{-TR/T_1}\right)$. The two unknowns, M_0 and T_1, were fitted for each voxel. T_1 values were reported in milliseconds (ms), and the relaxivity (r_1) values were determined from the plot of the $1/T_1$ against the Mn concentrations.

Linear regression was performed to estimate r_1 values according to the equation: $R_1(C) = R_1(0) + r_1 \cdot C$ where C is the concentration in Manganese of the molecules (Mn-TK, Mn-BR or Mn-[2]C). Units for r_1 is reported as $mM^{-1}s^{-1}$.

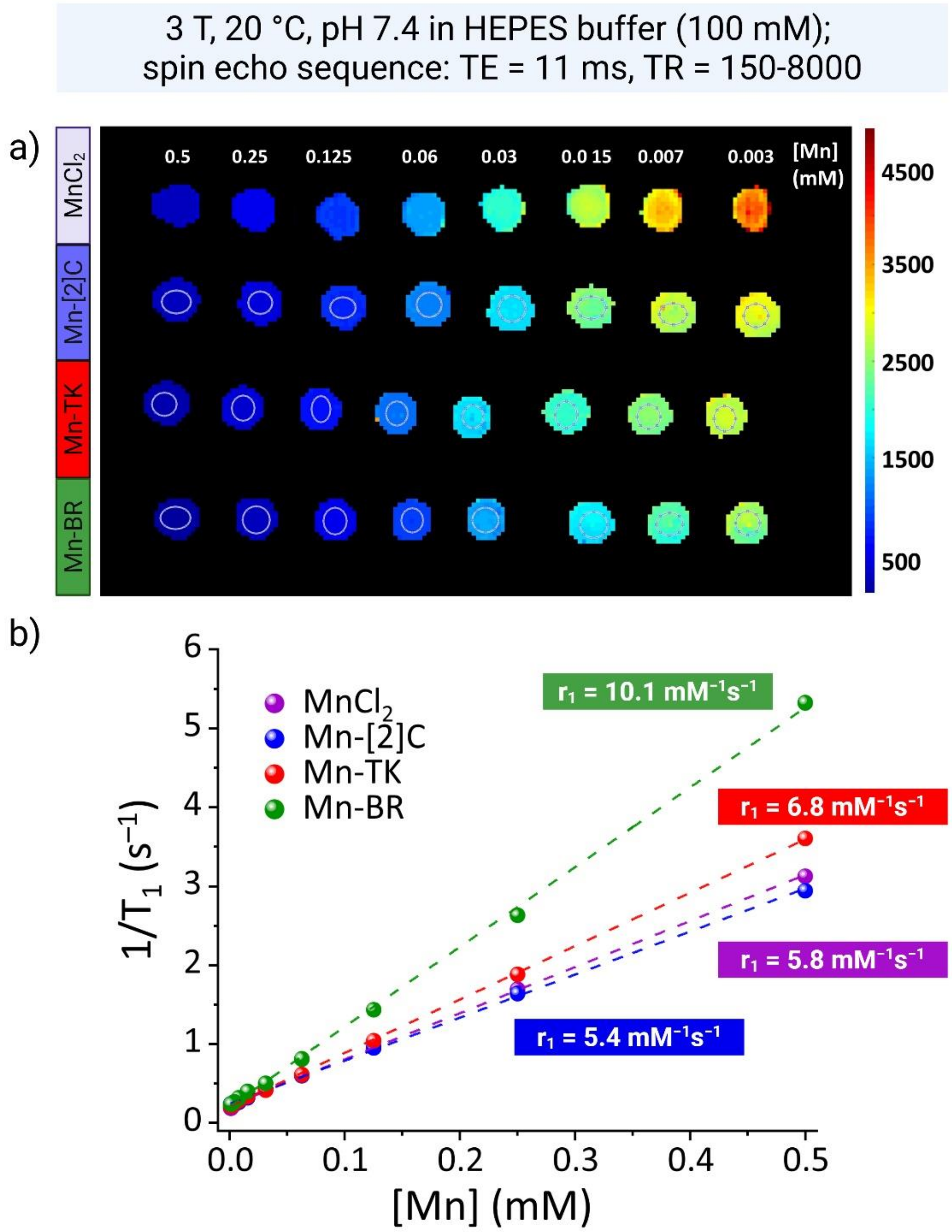

Figure S7. a) T_1-maps (units ms) and b) relaxation rate ($1/T_1$, s^{-1}) as function of Mn concentration (mM) for $MnCl_2$ (purple), Mn-[2]C (blue), Mn-TK (red) and Mn-BR (green) (3 T, 20 °C, pH 7.4 in HEPES buffer (100 mM); spin echo sequence: TE = 11 ms, TR = 150-8000

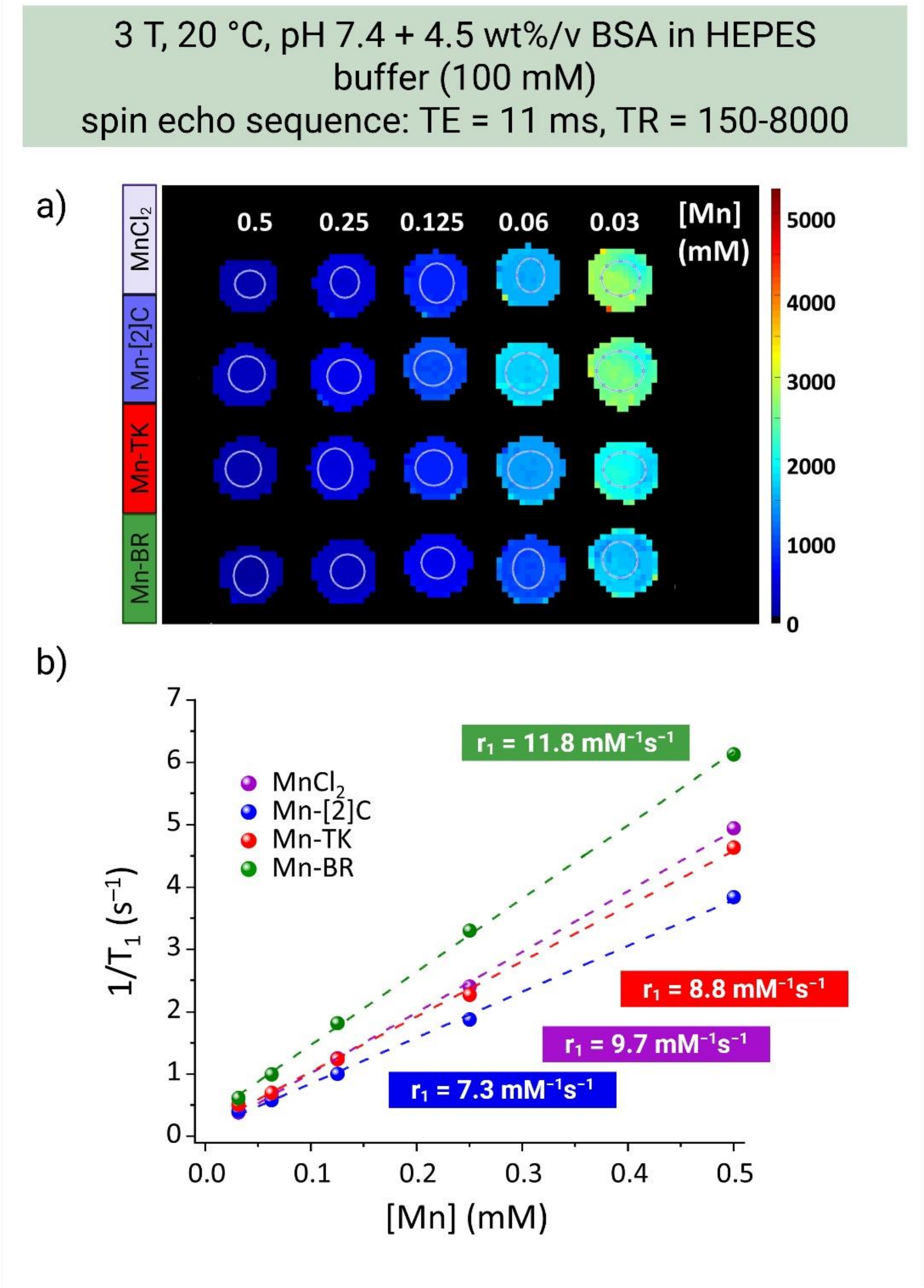

Figure S8. a) T_1-maps (units ms) and b) relaxation rate ($1/T_1$, s^{-1}) in presence of BSA as function of Mn concentration (mM) for $MnCl_2$ (purple), Mn-[2]C (blue), Mn-TK (red) and Mn-BR (green) (3 T, 20 °C, pH 7.4 in HEPES buffer (100 mM) supplemented with 4.5 wt%/m BSA; spin echo sequence: TE = 11 ms, TR = 150-8000).

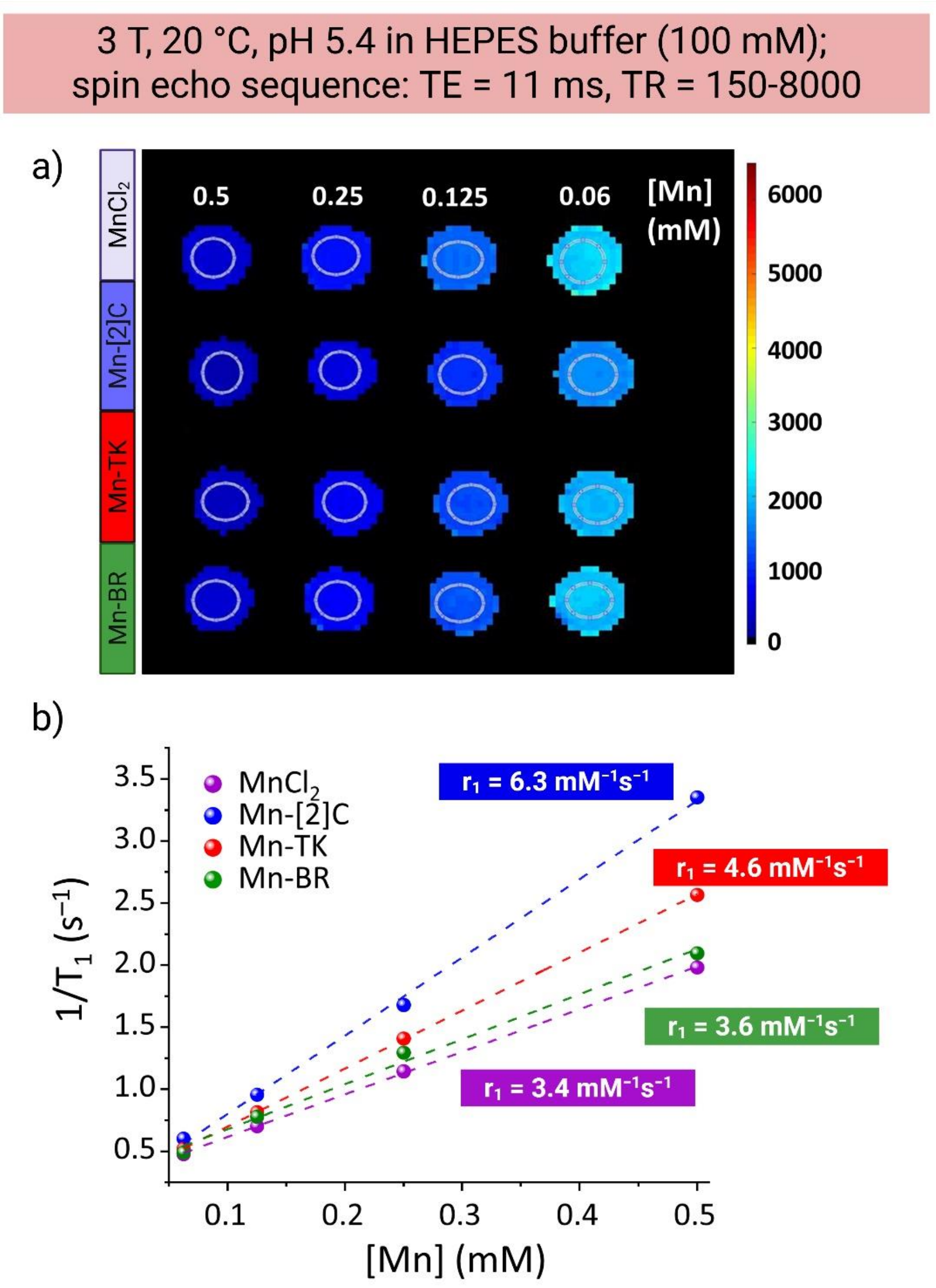

Figure S9. a) T_1-maps (units ms) and b) relaxation rate ($1/T_1$, s^{-1}) as function of Mn concentration (mM) for $MnCl_2$ (purple), Mn-[2]C (blue), Mn-TK (red) and Mn-BR (green) (3 T, 20 °C, pH 5.4 in HEPES buffer (100 mM); spin echo sequence: TE = 11 ms, TR = 150-8000).

Table S2. Comparison of Mn-based MRI contrast agents at 3 T.

Agent	Molecular Type	r_1 Relaxivity	Key Notes	Reference
$MnCl_2$ (Lumenhance)	Simple ionic salt	5–7	Non-chelated, rapid clearance, neurotoxicity risks	*PloS one* **2013,** *8* (3), e58617.[12]
Mn-DPDP (Teslascan)	Hepatobiliary chelate	1.5	Approved for liver imaging (withdrawn); high hepatic retention	*Investigative radiology* **2005,** *40* (11), 715-24.[13]
Mn-EDTA / Mn-DTPA	Linear chelates	2–4	Limited *in vivo* stability, rapid transmetallation	*Journal of Medicinal Chemistry* **2017,** *60* (7), 2993-3001.[14]
Mn-EDTA-BTA / Mn-DTPA copolymers	Polymeric chelates	3.5	Polymeric scaffold increases relaxivity & circulation	*Future Medicinal Chemistry* **2019,** *11* (12), 1461-1483[15]
Mn-NOTA	Macrocyclic chelate	2.8–3.5	Clinically relevant; stable macrocyclic chelate; moderate relaxivity	P*harmaceuticals (Basel, Switzerland)* **2023,** *16* (4)[16]
Mn-PyC3A	Macrocyclic chelate	3.5–4.2	New generation agent with high stability and improved excretion profile	*Molecules* **2023,** *28* (21)[17]
Mn(II) dendrimer conjugates	Dendrimer	6	Higher MW slows tumbling, boosting relaxivity	*Chemistry – A European Journal* **2014,** *20* (44), 14507-14513[18]
Macrocyclic Mn(II) complexes	Macrocyclic chelate	3–5	T_1 MRI agents via inner-sphere water relaxation	*Journal of Inorganic Biochemistry* **2022,** *228*, 111684[19]
Mn-porphyrins	Metalloporphyrin	5–10	Dual ROS scavenger + MRI probe	*Coordination Chemistry Reviews* **2021,** *445*, 214069[20]

Amino-Mn-porphyrin	Modified porphyrin	10	Enhanced membrane affinity	*Molecules* **2023,** *28* (21).[17]
Mn_3O_4 nanoparticles	MnO nanoparticle	1–5	High r_1 but aggregation & clearance issues	*Biomaterials* **2010,** *31* (14), 4073-8.[21]
Mn-NMOFs	MnO MOF-coated NP	4	Target-specific MR imaging *in vitro*	*J Am Chem Soc* **2008,** *130* (44), 14358-9.[22]
mX(Mn-dpama)$_2$/$_3$	Dipicolinic acid chelates	6–8	Binds human serum albumin, enhanced circulation	*Inorg Chem* **2015,** *54* (19), 9576-87.[23]
Mn-[2]C	Catenate	5.4	Minimal uptake, weak cancer selectivity	*This study*
Mn-TK	Trefoil Knot	6.8	High relaxivity, selective tumor uptake, pH-responsive	*This study*
Mn-BR	Borromean Ring	10.1	Multivalent Mn, strongest relaxivity, enhanced membrane targeting	*This study*

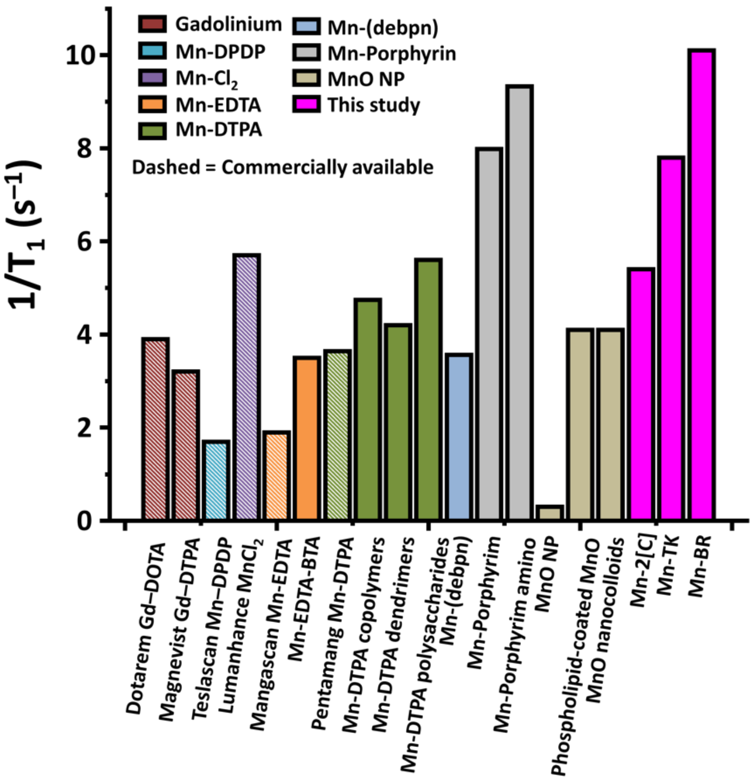

Figure S10. Comparison of r_1 relaxivities at 3 T for clinically used Mn chelates, polymeric and macrocyclic complexes, and our Mn-based non-trivial structures. Mn-[2]C, Mn-TK, and Mn-BR exhibit higher relaxivities than most conventional Mn chelates, with Mn-BR showing the strongest performance, matching or exceeding the best reported Mn porphyrins.

Table S3. Stability of longitudinal relaxivity (r_1) of Mn-based topological structures in HEPES buffer (pH 7.4) over 7 days. The longitudinal relaxivity (r_1) of $MnCl_2$, Mn-[2]C, Mn-TK, and Mn-BR was measured on Day 1 and Day 7 after incubation in HEPES buffer (pH 7.4).

Compound	Day 1 r_1 (mM^{-1} s^{-1})	Day 7 r_1 (mM^{-1} s^{-1})	Δr_1 (%)
$MnCl_2$	3.98	3.97	−0.25 %
Mn-[2]C	4.69	4.69	0.00 %
Mn-TK	6.13	6.13	0.00 %
Mn-BR	11.99	11.97	−0.17 %

6. *In vitro* biological studies

6.1. Cell culture

Human malignant glioblastoma (U251-MG, ATCC No. 09063001), breast adenocarcinoma (MCF-7, ATCC No. HTB-22), triple-negative breast adenocarcinoma (MDA-MB-231, ATCC No. HTB-26), cervical carcinoma (HeLa, ATCC No. CCL-2), hepatocellular carcinoma (HepG2, ATCC No. HB-8065), and colorectal adenocarcinoma (Caco-2, ATCC No. HTB-37) cell lines, along with non-cancerous controls including human embryonic kidney cells (HEK-293, ATCC No. CRL-1573), human lung fibroblasts (MRC-5, ATCC No. CCL-171), and human umbilical vein endothelial cells (HUVEC), were cultured in Dulbecco's Modified Eagle's Medium (DMEM) supplemented with 10% fetal bovine serum (FBS), 1% penicillin/streptomycin, and 20 mM L-glutamine at 37 °C in a humidified atmosphere containing 5% CO_2.

6.2. MR Imaging of U251-MG and HEK-293 Cells

HEK-293 or U251-MG cells were seeded in a 6-well culture plate in DMEM for 24 hours. Cells were incubated for 24 hours with no additives (control), $MnCl_2$, Mn-[2]C, Mn-TK and Mn-BR [Mn] = 10 µM). Cells were then washed with PBS three times and fixed with paraformaldehyde. Next, agarose solution (5 mL, PBS, 2 wt%) was added to each well and allowed to solidify at 4 °C.

Samples were placed in a transmit/receive quadrature knee coil within a clinical 3T MRI scanner (MAGNETOM Prisma, Siemens Healthineers, Erlangen, Germany) for quantitative T_1 relaxation mapping. A saturation recovery spin echo sequence was employed with the following parameters: variable repetition times (TR, in ms) of 150, 200, 300, 500, 800, 1000, 1500, 3000, 4000, and 8000 ms; echo time (TE) of 11 ms; field of view (FOV) of 130 mm × 130 mm; acquisition matrix of 320 × 320; and slice thickness of 3.5 mm.

The acquired data was processed using MATLAB 2024a (MathWorks Inc.) with a custom script for pixel-by-pixel nonlinear least squares fitting (utilizing the lsqcurvefit function). The signal at each voxel was plotted against its corresponding TR, and a T_1 recovery curve was fit using the $M(TR) =$

$M_0(1 - e^{-TR/T_1})$. The two unknowns, M_0 and T_1, were fitted for each voxel. T_1 values were reported in milliseconds (ms).

6.3. Quantification of Manganese

The quantitative analysis of manganese uptake was conducted using ICP-MS (Inductively Coupled Plasma Mass Spectrometry) using human cell lines, spanning five cancer types—MCF-7, HeLa, HepG2, MDA-MB-231, and Caco-2—and three non-cancerous lines—HEK-293, MRC-5, and HUVEC. Cells were cultured in 6-well plates using DMEM and incubated for 24 hours. The cells were treated for an additional 24 hours with either no additives (control), $MnCl_2$, Mn-[2]C, Mn-TK, or Mn-BR, maintaining a manganese concentration of 10 µM. Following treatment, cells were harvested, counted, and washed twice with PBS. The harvested cells were dissolved in nitric acid, and to prevent anomalously high readings, all samples were diluted tenfold. Manganese concentrations within the cells were subsequently measured using a Agilent 7800 series ICP-MS, and results were expressed as micrograms of manganese per cell.

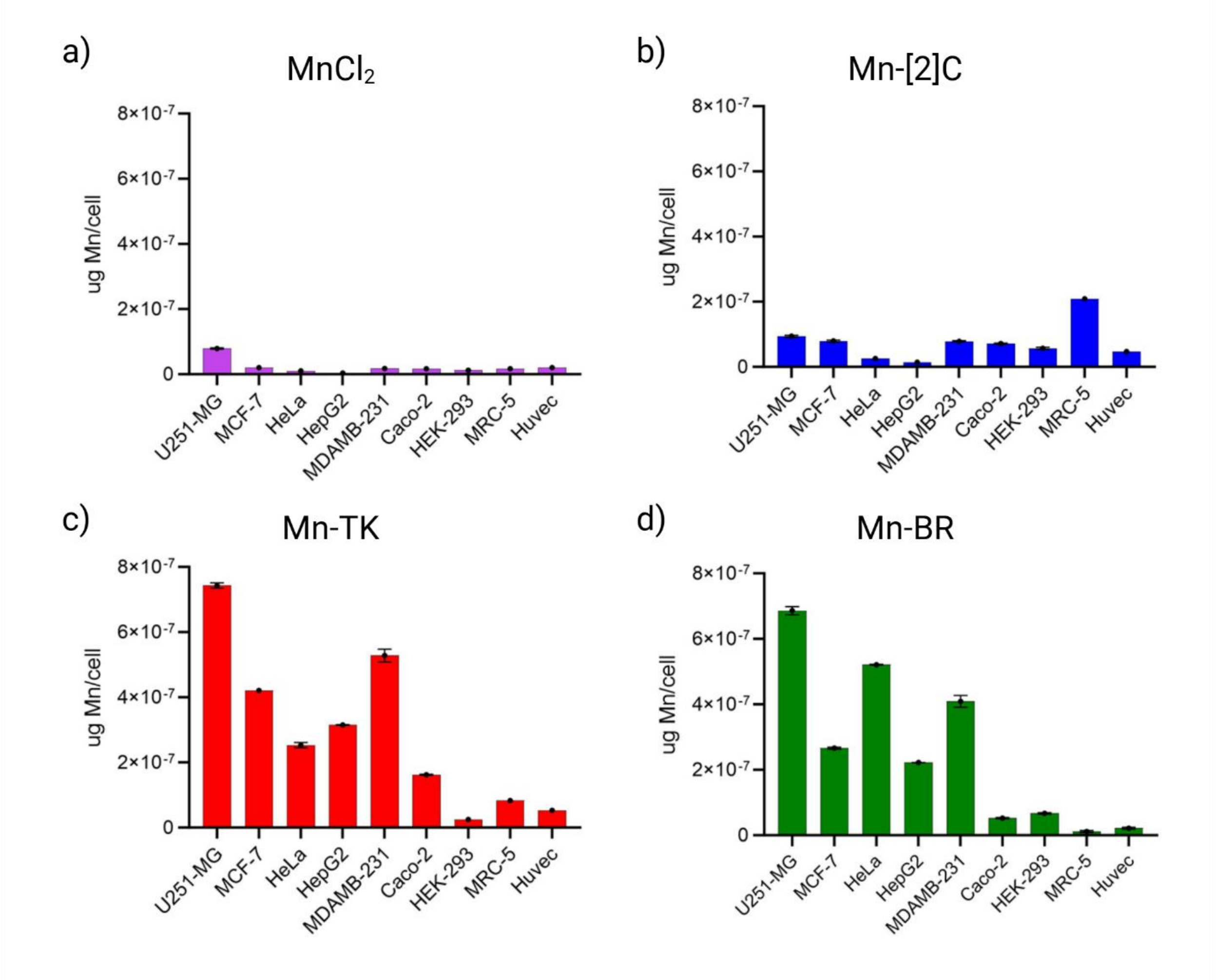

Figure S11. Quantitative ICP-MS analysis of intracellular manganese uptake across cancer and normal human cell lines after 24 h incubation with 10 µM Mn-based compounds. a) $MnCl_2$, b) Mn-[2]C, c) Mn-TK, and d) Mn-BR. Each plot shows Mn content (µg Mn $cell^{-1}$) in cancer cell lines—U251-MG (glioblastoma), MCF-7 (breast), HeLa (cervical), HepG2 (hepatocellular), MDA-MB-231 (triple-negative breast), and Caco-2 (colorectal)—compared with non-cancerous lines HEK-293 (embryonic kidney), MRC-5 (lung fibroblast), and HUVEC (endothelial). Data represent mean ± SD (n = 3).

6.4. *In vitro* Cell Toxicity Assesment

Cell viability was assessed using CellTiter-Blue® Cell Viability assay (CTB, Promega). The assay measures the metabolic reduction of a non-fluorescent compound, resazurin, into a fluorescent

product, resofurin, in living cells. As non-viable cells rapidly lose their metabolic activity, the amount of the resofurin product can be used to estimate the number of viable cells following treatment. Once produced, resofurin is released from living cells into the surrounding medium. Thus, the fluorescence intensity of the medium is proportional to the number of viable cells present.

We assessed cytotoxicity in cancer cell lines (MCF-7, HeLa, HepG2, MDA-MB-231, Caco-2) and non-cancer lines (HEK-293, MRC-5, HUVEC). Cells were seeded in 96-well plates at ~5×10^3 cells/well in 100 µL growth medium and allowed to adhere for 24 h (37 °C, 5% CO_2). Medium was then replaced with fresh medium containing no additive (control) or serial dilutions of test compounds and incubated for 48 h. Viability was measured with CellTiter-Blue by adding 20 µL reagent to 80 µL medium per well and incubating 6 h at 37 °C; resorufin fluorescence was recorded (λ_{ex} 560 nm/λ_{em} 590 nm). Signals were background-subtracted (medium + reagent, no cells), normalized to the no-additive control (100%), and fitted to a four-parameter logistic model to obtain IC_{50} values.

Viability and inhibition were calculated as:

$$\text{Viability (\%)} = [(F_{treated} - F_{blank}) / (F_{control} - F_{blank})] \times 100$$

$$\text{Inhibition (\%)} = 100 - \text{viability (\%)}$$

Data are reported as mean ± SD; each condition was tested in technical triplicate and reproduced in ≥3 independent experiments. IC_{50} values are the mean ± SD from these replicates, determined from plots of inhibition (%) versus compound concentration (µM).

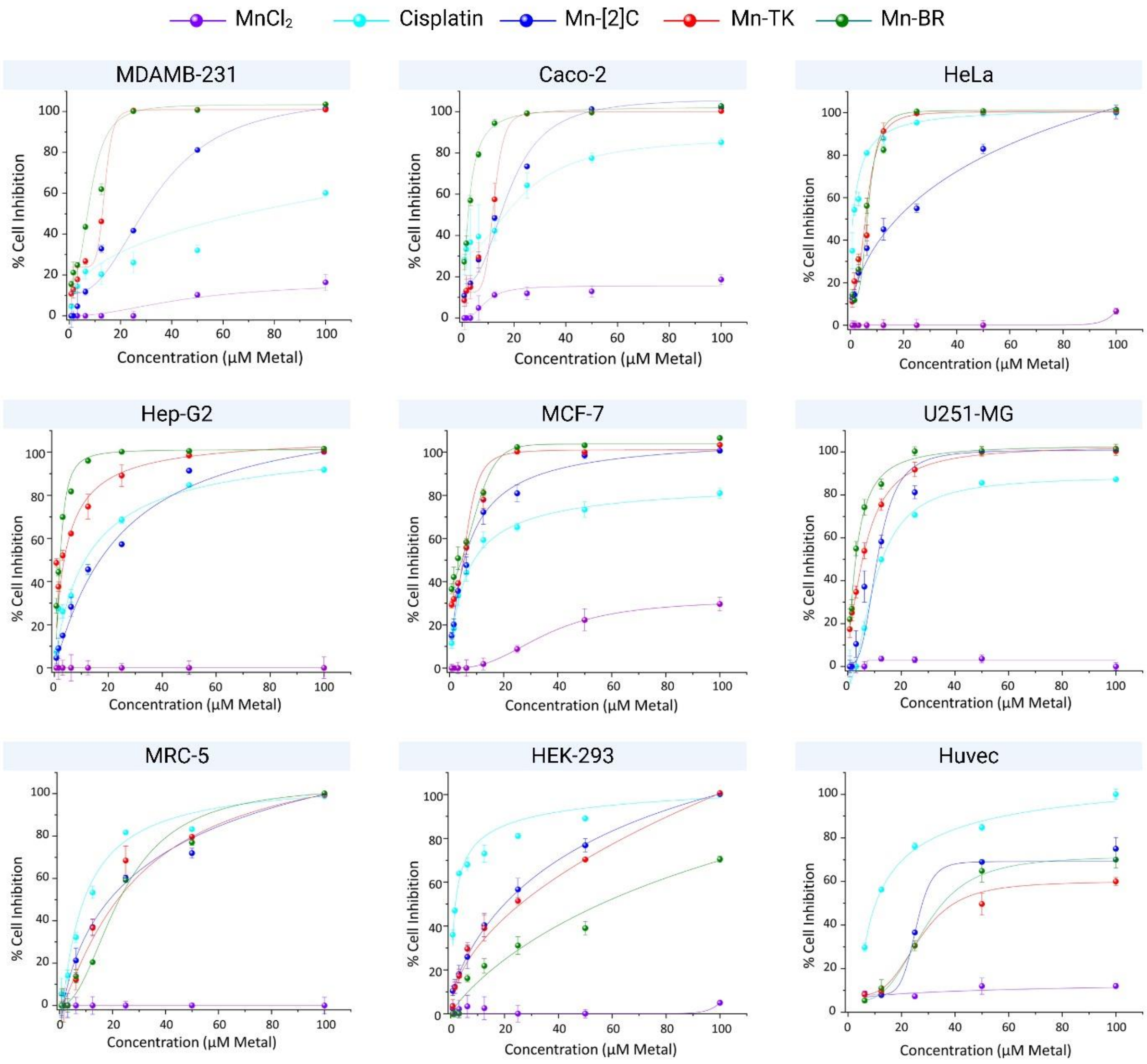

Figure S12. Dose–response curves for $MnCl_2$, cisplatin, Mn-[2]C, Mn-TK, and Mn-BR in nine human cell lines. Cancer cell lines: MDAMB-231, Caco-2, HeLa, Hep-G2, MCF-7, and U251-MG. Normal cell lines: MRC-5, HEK-293, and HUVEC. Cells were treated for 48 h, and viability was measured by CTB assay. Data are expressed as mean ± SD (n = 3).

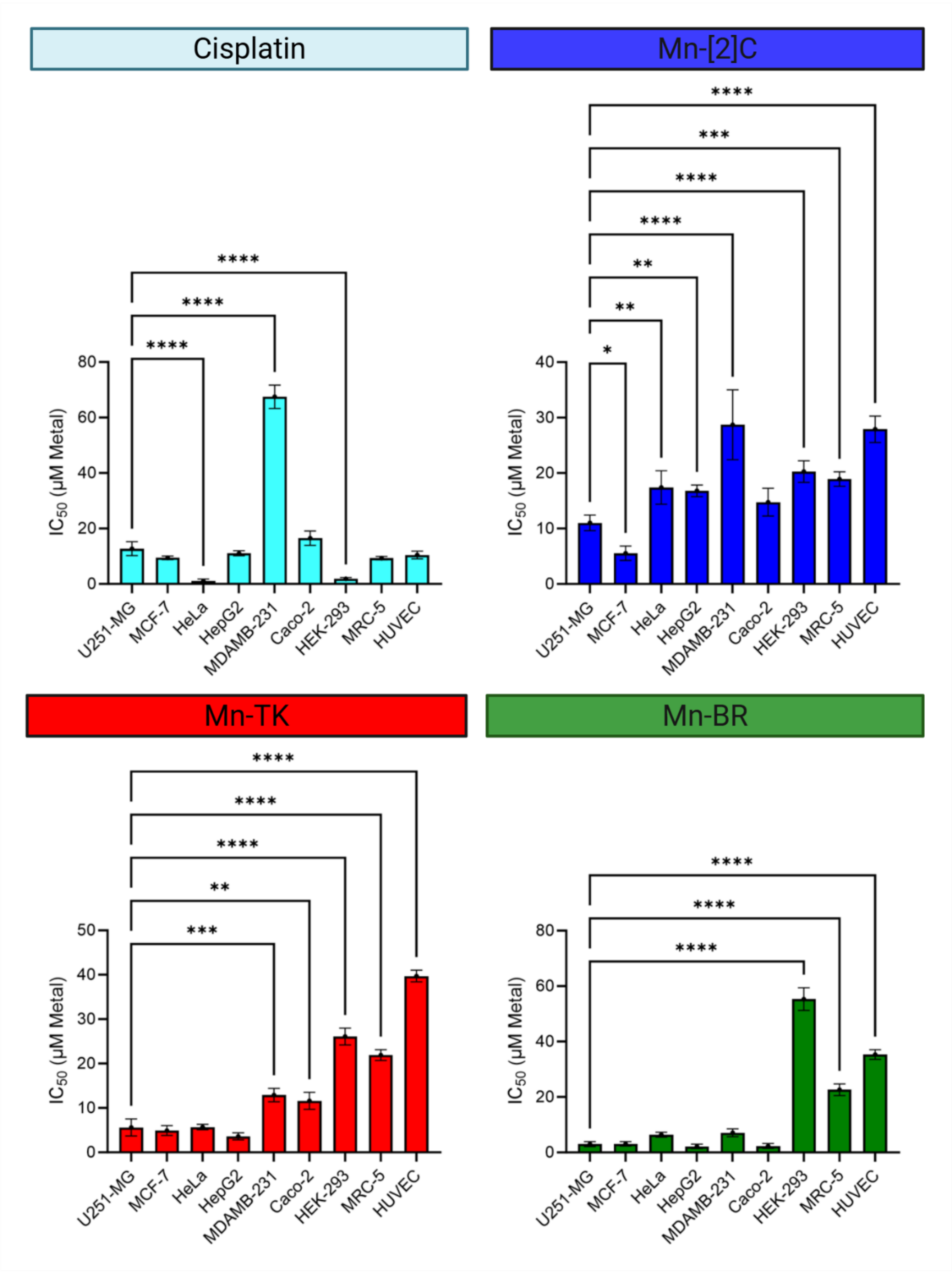

Figure S13. Comparison of IC$_{50}$ values across cancer and normal cell lines for cisplatin and Mn-based compounds. Bar graphs show IC$_{50}$ values (µM metal) of cisplatin, Mn-[2]C, Mn-TK, and Mn-BR, measured in nine human cell lines after 48 h treatment. Cancer cell lines include U251-MG,

MCF-7, HeLa, HepG2, MDA-MB-231, and Caco-2; normal cells include HEK-293, MRC-5, and HUVEC. Data are presented as mean ± SD from three independent experiments. Statistical comparisons were made relative to U251-MG for each compound using one-way ANOVA with Tukey's post-test. *$p < 0.05$, **$p < 0.01$, ***$p < 0.001$, ****$p < 0.0001$.

6.5. Hemolysis Assay

When the external membrane of the erythrocytes is destroyed, hemoglobin is released.[24-26] It is possible to estimate the amount of destroyed erythrocytes in a given test by measuring the quantity of hemoglobin in a sample by spectrophotometry.[27]

Human blood was obtained from 3 healthy donors. 2.0 mL of an ethylenediaminetetraacetate-stabilized blood sample was added into 4 mL of physiological saline buffer (PBS), and then red blood cells were isolated by centrifugation (3000 rpm, 8 min). The red blood cells were washed five times with physiological saline and diluted into 2 % red blood cell suspensions.

Subsequently, $MnCl_2$, cisplatin, Mn-[2]C, Mn-TK, or Mn-BR (10 μM Metal) were added into the red blood cell suspensions at the predetermined concentration and mixed using a gentle vortex. Meanwhile, physiological saline with or without Triton X-100 (0.3 %) was added into the red blood cell suspensions as negative and positive controls, respectively. Samples were placed in a static condition at 37 °C for 1 h. Finally, all samples were centrifuged at 5000 rpm, and 100 μL of the supernatant was placed into a 96-well plate for detection at the wavelength of 540 nm. The hemolysis ratio (HR) represents the degree of red blood cell membranes destroyed in the samples.

$$\mathbf{HR\ (\%)} = \frac{A_{sample} - A_{negative\ control}}{A_{positive\ control} - A_{negative\ control}} \times 100$$

A_{sample}, $A_{positive\ control}$, and $A_{negative\ control}$ represented the absorbance of the sample, the positive control, and the negative control, respectively. These tests were performed in triplicate.

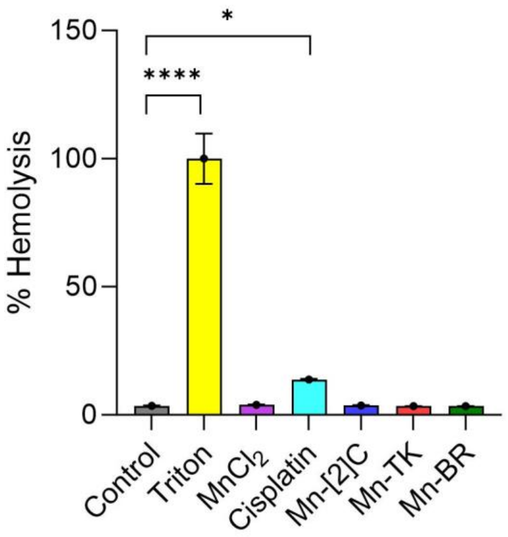

Figure S14. Hemolysis activity of Mn-based compounds compared to controls. Red blood cells (2% suspension) were incubated for 1 h at 37 °C with $MnCl_2$, cisplatin, Mn-[2]C, Mn-TK, or Mn-BR (10 µM Metal). Physiological saline (PBS) and 0.3% Triton X-100 were used as negative (C–) and positive (Triton) controls, respectively. Hemoglobin release was quantified at 540 nm, and the hemolysis rate (HR%) was calculated relative to Triton (100%). Data represent mean ± SD from three independent experiments; ****$p < 0.0001$ vs. negative control.

6.6. Kinetic of Uptake

Kinetic manganese uptake was quantified in U251-MG cells by ICP-MS. Cells were seeded in 6-well plates complete DMEM and allowed to adhere for 24 h. Monolayers were then exposed for 1, 4, or 24 h to no additive (control) or 10 µM Mn as $MnCl_2$, Mn-[2]C, Mn-TK, or Mn-BR. After treatment, the media were removed, and the cells were washed 3× with PBS. Cells were detached, counted, pelleted, and digested in ultrapure HNO_3 (trace-metal grade; final 2–3% v/v) for ≥2 h at room temperature. Digests were diluted 10× and analyzed on an Agilent 7800 ICP-MS against matrix-matched Mn standards (0–100 ppb). Manganese uptake was reported as µg Mn per 10^6 cells. Data were background-subtracted (acid and buffer blanks) and expressed as mean ± SD from ≥3 independent experiments.

6.7. Endocytic Inhibitor

U251-MG cells were seeded in 6-well plates in complete DMEM and allowed to adhere for 24 h. On the day of the assay, cells were pre-treated for 1 hour with pathway inhibitors at the following working concentrations: filipin III 2 μg mL^{-1} (caveolae/lipid rafts), methyl-β-cyclodextrin (MβCD) 5 mM (cholesterol depletion), cytochalasin D 2 μM (actin/macropinocytosis), chlorpromazine (CPZ) 10–15 μg mL^{-1} (clathrin), and amiloride (50–100 μM, macropinocytosis). For energy depletion, parallel plates were pre-equilibrated on ice, and all solutions were pre-chilled; cells were then handled and incubated at 4 °C. Immediately after pre-treatments, compounds were added to give 10 μM Mn as $MnCl_2$, Mn-[2]C, Mn-TK, or Mn-BR; inhibitors (except MβCD) were maintained during the 4 h uptake period, whereas MβCD-treated wells were washed once with warm buffer before compound addition. At 4 h, the media were removed, and the monolayers were washed 3× with PBS. Cells were detached, counted, pelleted, and digested in trace-metal-grade HNO_3. Digests were diluted 10 and Mn was quantified by ICP-MS. data (n = 3 wells/condition, ≥3 independent experiments) were analyzed by one-way ANOVA with appropriate post-hoc multiple-comparison testing versus control at 37 °C.

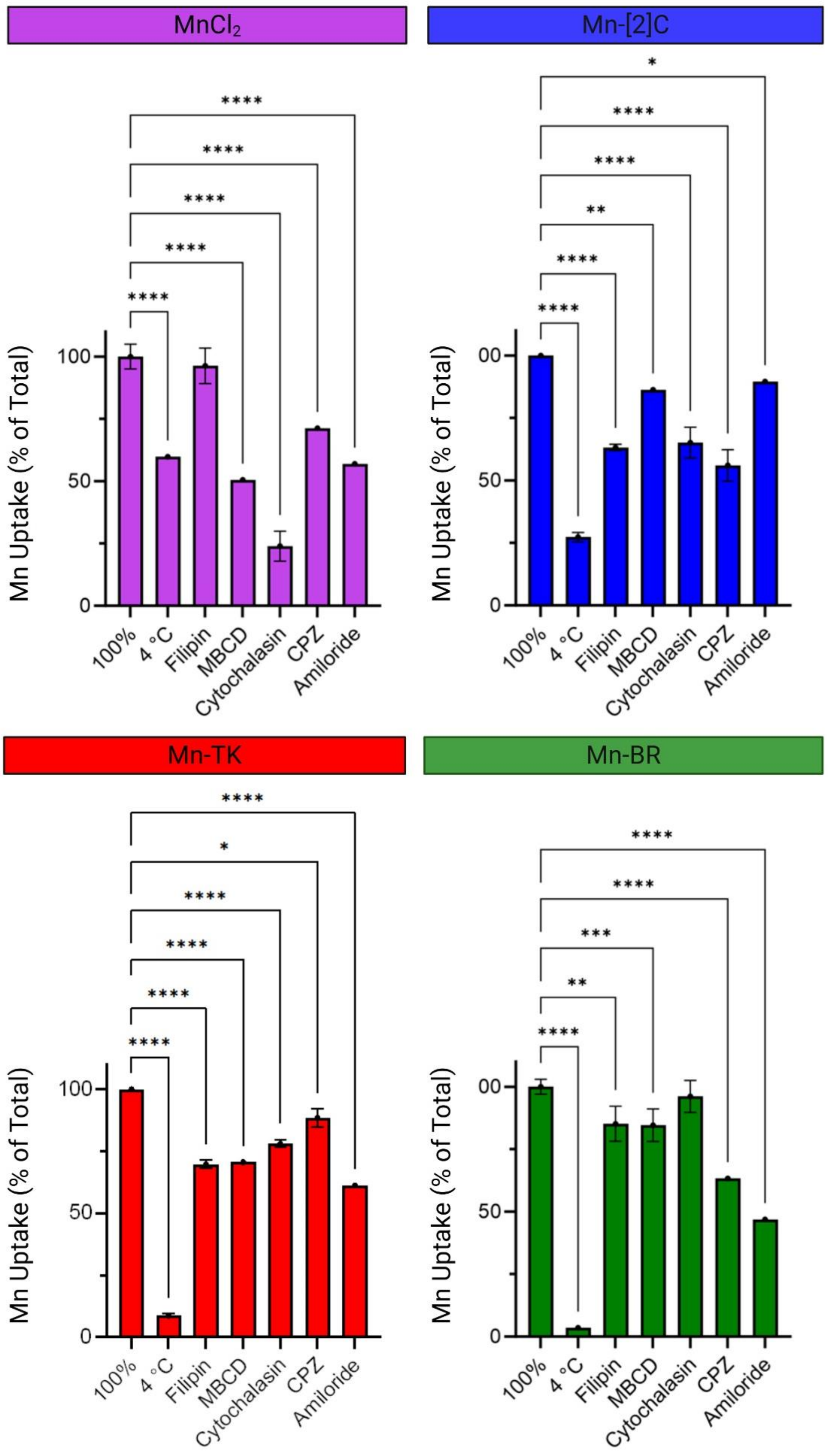

MnCl$_2$
Mn-[2]C
Mn-TK
Mn-BR
Mn Uptake (% of Total)
100
00
50
0
100%
4 °C
Filipin
MBCD
Cytochalasin
CPZ
Amiloride
****
***
**
*

Figure S15. Endocytosis inhibitor assay in U251 MG cells. Cellular uptake of $MnCl_2$ (purple), Mn-[2]C (blue), Mn-TK (red), and Mn-BR (green) was quantified by ICP-MS after 4 h incubation in the presence of energy depletion (4 °C) or specific endocytosis inhibitors: Filipin and MβCD (disrupt lipid rafts/caveolae-mediated endocytosis), Cytochalasin D (inhibits actin polymerization/macropinocytosis), CPZ (clathrin-mediated endocytosis inhibitor), and Amiloride (macropinocytosis inhibitor). Data represent mean ± SD (n = 3); significance was determined using one-way ANOVA with post-hoc tests (*$p < 0.05$; **$p < 0.01$; ***$p < 0.001$; ****$p < 0.0001$).

6.8. Subcellular Fractionation of U251-MG Cells: Nuclear, Cytosolic, Cytoplasmic, Mitochondrial, and Membrane Fractions

U251-MG glioblastoma cells were seeded in 6-well plates at a density of 5×10^5 cells per well and treated with Mn-[2]C, Mn-TK, or Mn-BR at a final manganese concentration of 10 µM for 24 hours. Each treatment condition was performed in triplicate. Following incubation, cells were subjected to subcellular fractionation using the Thermo Scientific™ Mitochondria Isolation Kit for Cultured Cells (Cat# 89874) and the PARIS™ Kit (Cat# AM1921), following the manufacturers' protocols.

Isolation of Mitochondrial and Cytosolic Fractions

Cells were harvested by centrifugation at 850 × g for 2 minutes at 4°C. The supernatant was discarded, and the pellet was resuspended in 800 µL of Mitochondria Isolation Reagent A. The suspension was vortexed for 5 seconds at medium speed and incubated on ice for 2 minutes.

Subsequently, 10 µL of Mitochondria Isolation Reagent B was added. The mixture was vortexed at maximum speed for 5 seconds, then incubated on ice for 5 minutes, with intermittent vortexing every minute. Next, 800 µL of Mitochondria Isolation Reagent C was added, and the suspension was centrifuged at 700 × g for 10 minutes at 4°C.

The resulting supernatant was transferred to a clean tube and centrifuged again at 12,000 × g for 15 minutes at 4°C to separate the cytosolic fraction (supernatant) from the mitochondrial pellet. The mitochondrial pellet was washed with 500 µL of Reagent C and centrifuged once more at

12,000 × g for 5 minutes. Both fractions were stored on ice for immediate analysis or frozen at −80°C for later use.

Isolation of Cytoplasmic, Membrane, and Nuclear Fractions

The remaining cells were lysed using the Cell Fractionation Buffer from the PARIS™ Kit and incubated on ice for 5–10 minutes to ensure complete plasma membrane disruption. The lysate was centrifuged at 500 × g for 5 minutes at 4°C to pellet the nuclei.

Cytoplasmic Fraction: The cytoplasmic supernatant was carefully collected and stored for RNA/protein isolation or additional analyses.

Nuclear Fraction: The nuclear pellet was resuspended in Cell Disruption Buffer, vortexed for 15 seconds, and incubated on ice for 10 minutes. Following lysis, the suspension was briefly centrifuged, and the supernatant containing solubilized nuclear proteins was collected for downstream analysis.

Membrane Fraction: To isolate the membrane fraction, the original cytoplasmic supernatant was subjected to differential centrifugation at 16,000 × g for 30 minutes at 4°C. The pellet, enriched in membrane-associated material, was collected and resuspended in an appropriate buffer for protein assays or immunoblotting.

6.9. Intracellular Distribution Study using TEM

For TEM analysis, U251-MG cells were seeded in T75 flasks in complete DMEM and incubated for 24 hours with cell-medium alone (control), Mn-[2]C, Mn-TK, and Mn-BR ([Mn] = 10 µM) in DMEM. After harvesting, the cell pellets were washed twice with PBS. The cells were cryo-fixed within a few milliseconds at a pressure of 2000 bar under liquid nitrogen using a high-pressure freezer (Leica Microsystems, Germany). After freezing, the sample pod was released automatically into a liquid nitrogen bath. While still in liquid nitrogen, the sample carrier was separated from the specimen pod using precooled fine-tipped tweezers and transferred to the cryo-transfer storage box for the flat specimen carrier, where the samples were stored in preparation for freeze substitution. Freeze

substitution was performed using an automatic freeze substitution (AFS) unit (Leica EM AFS2, Heerbrugg, Switzerland) in a 10 mL solution of cold, dry absolute acetone (v/v) containing 1 % osmium tetroxide (w/v), 0.5 % uranyl acetate (w/v) and 5 % distilled water (v/v). The AFS unit was slowly warmed from −90 °C to 0 °C (2 °C/hour), with the temperature being held at both −60 °C and −30 °C for 8 hours. Samples were transferred to room temperature in a closed container to prevent condensation, rinsed with absolute acetone (3 × 5 minutes), and infiltrated with 30, 60, and 100 % Epon resin for 3 hours each. Epon was exchanged, and individual samples were embedded in 1 mL Eppendorf® lids for 24 hours at 60 ˚C. Finally, the samples were sectioned with an ultra-microtome at room temperature using a diamond knife, and the ultrathin sections were examined under TEM (Talos F200X STEM). The experiment was performed in triplicate.

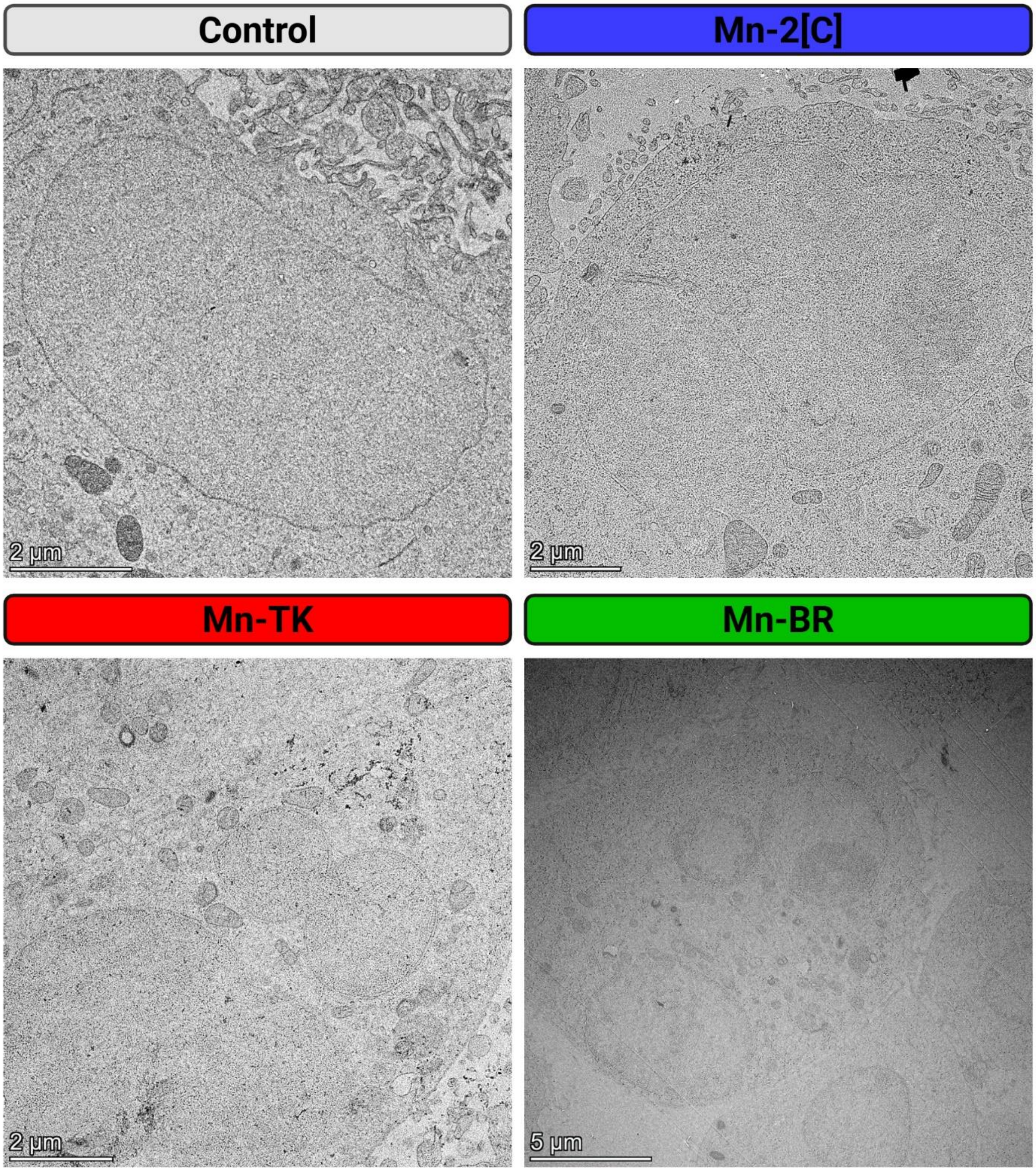

Figure S16. TEM zoom of nuclei in U251-MG cells. Control: intact nucleus. Mn-[2]C: early chromatin condensation. Mn-TK: nuclear fragmentation (karyorrhexis). Mn-BR: collapsed nuclear structure/apoptotic body.

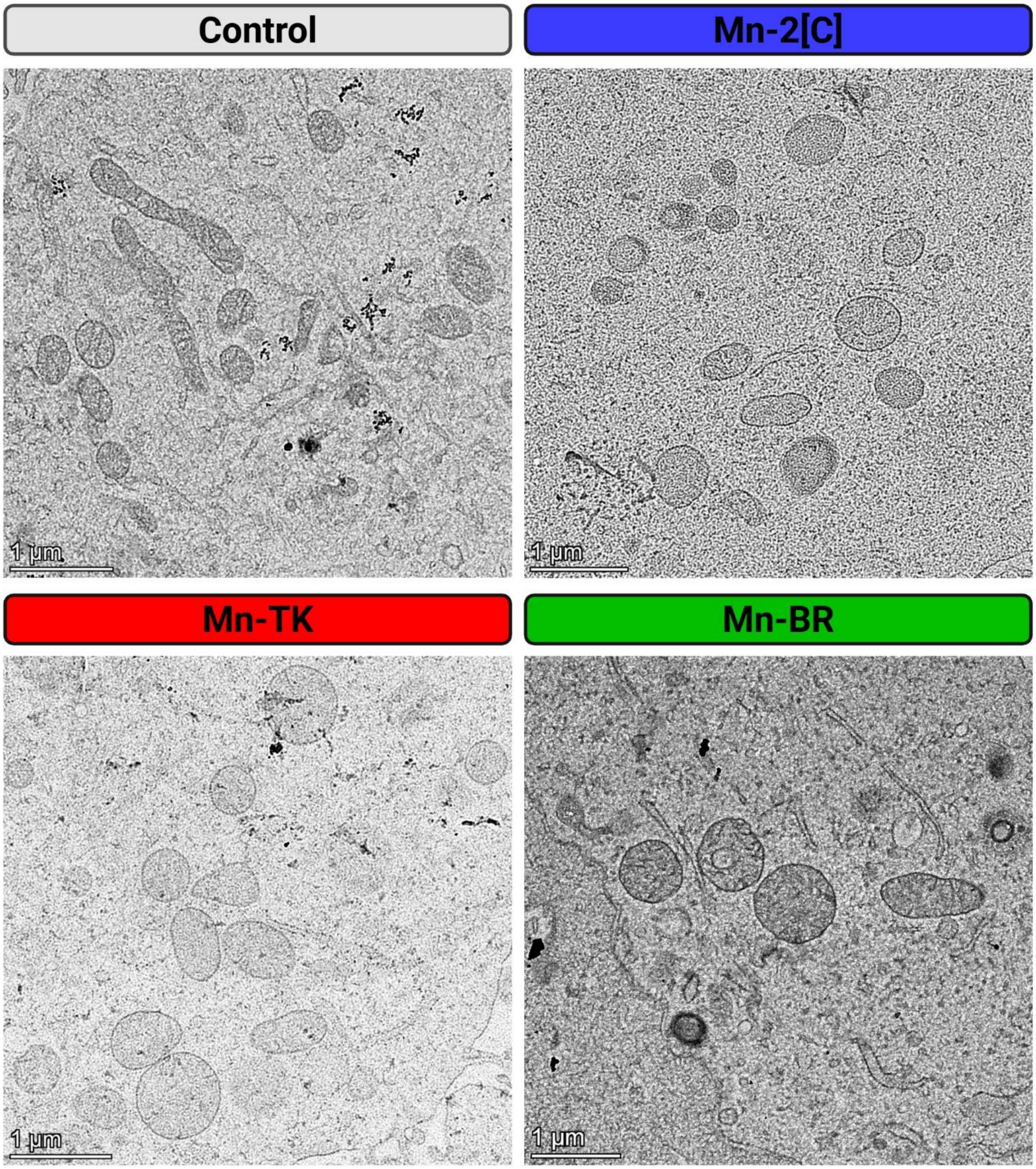

Figure S17. TEM zoom of mitochondria in U251-MG cells. Control: elongated mitochondria with dense, well-defined cristae. Mn-[2]C: mild swelling/rounding with subtle cristae rarefaction. Mn-TK: swollen mitochondria with clear cristae disruption. Mn-BR: severe swelling and marked loss of cristae, consistent with advanced mitochondrial damage. Scale bars: 1 µm.

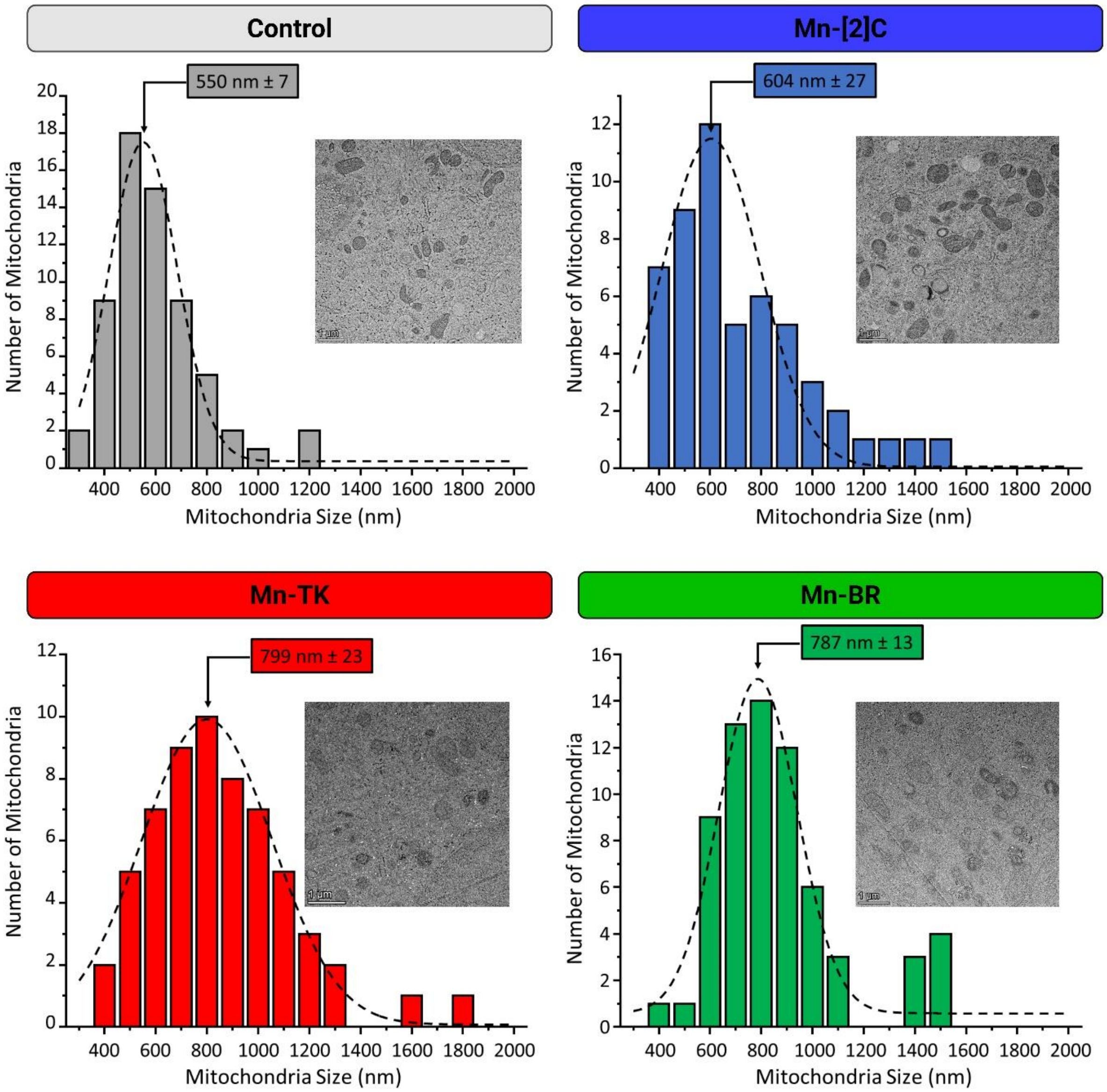

Figure S18. Mitochondrial Size Distribution in U251-MG Cells After Treatment with Metal-Organic Compounds. This figure presents histograms of mitochondrial size distributions measured by Transmission Electron Microscopy (TEM) for U251-MG glioma cells under various treatment conditions. Each panel corresponds to a different treatment, showing the frequency of mitochondria across a range of sizes: Control (Gray): The mitochondria predominantly have a size around 550 nm ± 7 nm, indicative of a typical population in untreated cells. Mn-[2]C (Blue): The peak size shifts slightly to 604 nm ± 27 nm, suggesting early signs of mitochondrial swelling possibly related to initial apoptotic changes. Mn-TK (Red): Displays a significant shift with most

mitochondria around 799 nm ± 23 nm, reflecting severe mitochondrial swelling associated with advanced apoptosis. Mn-BR (Green): Similar to Mn-TK, mitochondria show a substantial increase in size to approximately 787 nm ± 13 nm, also indicative of advanced apoptosis. The histograms are overlaid with dashed lines illustrating the average mitochondrial size for each treatment, and insets show representative TEM images of the mitochondria for each condition. These data highlight the morphological changes induced by each treatment, correlating mitochondrial swelling with the progression of apoptotic processes.

6.10. Cell Apoptosis Detection Using Annexin V-FITC and Propidium Iodide (PI) Staining

Apoptosis was evaluated using Annexin V-FITC/propidium iodide (PI) dual staining followed by flow cytometric analysis. During early apoptosis, phosphatidylserine (PS) translocates from the inner to the outer leaflet of the plasma membrane, where it is detected by Annexin V-FITC. PI, a membrane-impermeable nucleic acid dye, penetrates only late apoptotic or necrotic cells, enabling discrimination between viable, early apoptotic, and late apoptotic/necrotic populations.

U251-MG cells were seeded in 6-well plates in complete DMEM and allowed to adhere for 24 h. Cells were then treated with cisplatin, Mn-[2]C, Mn-TK, or Mn-BR at a final manganese (or equivalent drug) concentration of 10 µM for 24 h. Untreated cells served as control.

Following treatment, both adherent and floating cells were collected to ensure inclusion of all cell populations. Cells were washed twice with cold PBS and resuspended in 1× binding buffer at a concentration of approximately 1×10^6 cells/mL.

For each sample, 500 µL of cell suspension was transferred to a flow cytometry tube, and 5 µL Annexin V-FITC and 10 µL PI (BD Biosciences Apoptosis Detection Kit) were added. Samples were gently vortexed and incubated for 10 min at room temperature in the dark.

Flow cytometric analysis was performed using a BD FACSCalibur or Attune NxT cytometer equipped with a 488 nm excitation laser. Fluorescence emission was collected using the FITC channel (530 ± 15 nm) for Annexin V and the PI channel (585–620 nm) for PI. A minimum of 5×10^4 events per sample were acquired. Data were analyzed by quadrant gating to distinguish

viable (Annexin V⁻/PI⁻), early apoptotic (Annexin V⁺/PI⁻), late apoptotic (Annexin V⁺/PI⁺), and necrotic (Annexin V⁻/PI⁺) populations. Each condition was analyzed in duplicate, and all experiments were independently repeated three times. Results are presented as mean ± SD.

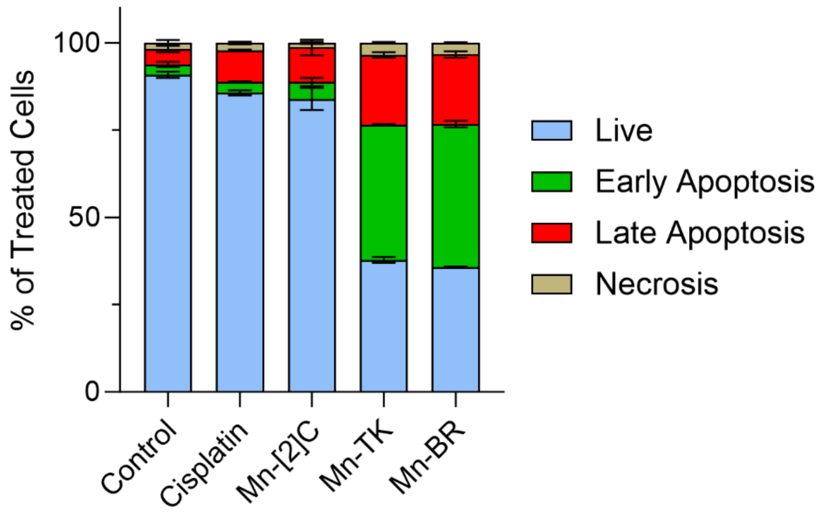

Figure S19. Quantitative Annexin V–FITC/Propidium Iodide (PI) apoptosis analysis of U251-MG cells after 24 h treatment. Cells were treated with Mn-[2]C, Mn-TK, Mn-BR, or cisplatin (10 µM Mn or equivalent drug concentration), and apoptotic populations were analyzed by flow cytometry. Stacked bar graphs represent the percentage of viable (Annexin V^-/PI^-, blue), early apoptotic (Annexin V^+/PI^-, green), late apoptotic (Annexin V^+/PI^+, red), and necrotic (Annexin V^-/PI^+, beige) cells. Data are presented as mean ± SD (n = 3 independent experiments).

Table S4. Quantitative analysis of apoptosis in U251-MG cells after 24 h treatment with Mn-[2]C, Mn-TK, Mn-BR, and cisplatin (10 µM Mn or equivalent drug concentration), as determined by Annexin V–FITC/propidium iodide (PI) flow cytometry. Data are presented as mean ± SD (%, n = 3 independent experiments) for viable (Annexin V^-/PI^-), early apoptotic (Annexin V^+/PI^-), late apoptotic (Annexin V^+/PI^+), and necrotic (Annexin V^-/PI^+) cell populations. Statistical analysis was performed using one-way ANOVA followed by Tukey's post hoc test, comparing each treatment group to the untreated control. Significance levels are indicated as ****$p < 0.0001$.

Treatment	Live (%)	Early Apoptosis (%)	Late Apoptosis (%)	Necrosis (%)
**Control**	90.85 ± 0.85	2.99 ± 0.75	4.36 ± 0.92	1.83 ± 0.80
**Cisplatin**	85.65 ± 0.75 ****	3.14 ± 0.19	9.01 ± 0.22 ****	2.18 ± 0.37
**Mn-[2]C**	83.90 ± 3.20 ****	4.90 ± 1.20	9.86 ± 2.24 ****	1.32 ± 0.25
**Mn-TK**	37.80 ± 0.80 ****	38.70 ± 0.20 ****	20.00 ± 0.80 ****	3.56 ± 0.21
**Mn-BR**	35.75 ± 0.15 ****	40.95 ± 0.95 ****	19.95 ± 0.95 ****	3.30 ± 0.20

6.11. Live-Cell Annexin V Assay

U251-MG cells were seeded in 96-well plates (5000 cells/well) with complete DMEM and allowed to adhere for 24 h. Cells were then exposed to $MnCl_2$, Mn-[2]C, Mn-TK, or Mn-BR, cisplatin (10 µM Metal), or no additive (control), and maintained at 37 °C with 5% CO_2. Annexin V Green (Sartorius, Cat. No. 4642) was added directly to the culture medium. Live-cell imaging was performed in situ at 24 h and 48 h using a Lionheart FX automated fluorescence microscope (BioTek). For imaging, brightfield and green fluorescence images were collected across the whole well. Annexin V–positive cells were quantified using Gen5 software with a predefined image-analysis pipeline. Data were expressed as mean ± SD from three independent experiments, and statistical significance at each time point was determined by one-way ANOVA with post-hoc tests; ns, p<0.05, *p<0.01, **p<0.001, ***p<0.0001.

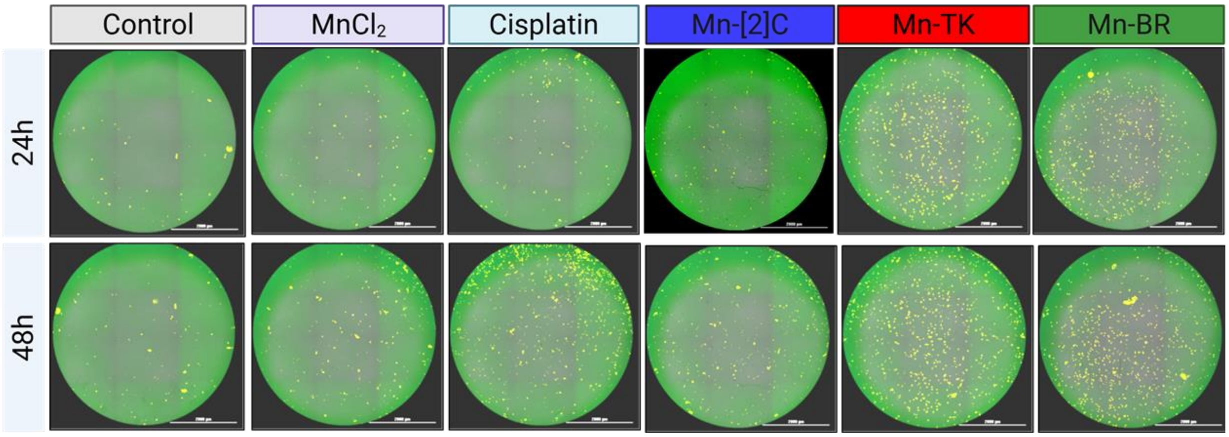

Figure S20. Live-cell Annexin V/PI images of U251-MG cells treated with $MnCl_2$, Mn-[2]C, Mn-TK, Mn-BR, and cisplatin (10 µM Metal) at 24 h and 48 h (cisplatin positive control) illustrate a time-dependent rise in Annexin-positive cells for Mn-TK and Mn-BR (scale bar 200 µm).

6.12. Western Blotting

U251-MG cells were seeded in 6-well plates and allowed to reach approximately 80% confluency in complete DMEM. After 24 h, cells were treated with vehicle (control), cisplatin, Mn-[2]C, Mn-TK, or Mn-BR at a final concentration of 10 µM Mn (or equivalent drug concentration for cisplatin) for 24 h.

Following treatment, both adherent and floating cells were collected, washed twice with ice-cold PBS, and pelleted by centrifugation. Cell pellets were lysed in 200 µL NP-40 lysis buffer (150 mM NaCl, 1% NP-40, 50 mM Tris-HCl, pH 8.0) supplemented with protease inhibitor cocktail (Roche, Cat# 11697498001). Lysates were incubated on ice for 30 min with intermittent vortexing and clarified by centrifugation at 12,000 × g for 15 min at 4 °C. The supernatants were collected, and protein concentrations were determined using a BCA assay.

Equal amounts of protein (30 µg per lane) were resolved on 10% Mini-PROTEAN® TGX™ precast gels (Bio-Rad, Cat# 4561033) and transferred onto nitrocellulose membranes. Membranes were blocked in 5% non-fat milk prepared in TBS-T (Tris-buffered saline containing 0.1% Tween-20) for 1 h at room temperature and then incubated overnight at 4 °C with primary antibodies against

Caspase-3 (BioLegend, Cat# 949802), Caspase-8 (BioLegend, Cat# 645501), and GAPDH (Santa Cruz Biotechnology, Cat# sc-32233).

After washing with TBS-T, membranes were incubated for 1 h at room temperature with HRP-conjugated anti-rat IgG secondary antibody (Cell Signaling Technology, Cat# 7074). Protein bands were detected using Clarity™ Western ECL substrate (Bio-Rad, Cat# 1705060) and visualized with a ChemiDoc™ imaging system (Bio-Rad). Band intensities were quantified using Fiji (ImageJ) software and normalized to GAPDH. All experiments were performed in triplicate.

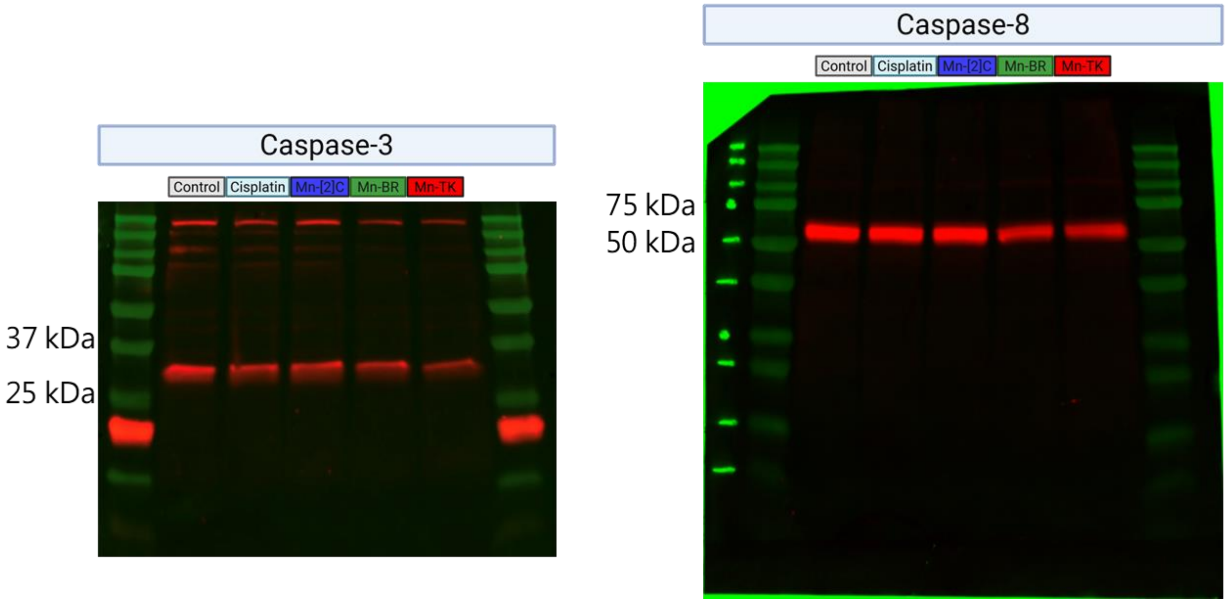

Figure S21. Western blot analysis of apoptotic markers in U251-MG cells. Representative immunoblots showing total caspase-3 and caspase-8 expression in U251-MG cells treated for 24 h with cisplatin, Mn-[2]C, Mn-BR, or Mn-TK (10 µM Mn or equivalent drug concentration). Control cells received no treatment. Reduced intensity of full-length caspase bands in treated samples is consistent with caspase activation and apoptotic processing. Molecular weight markers are shown on the sides. Blots are representative of three independent experiments.

6.13. LDH Release Experiment

U251-MG, HeLa, and HEK-293 cells were cultured in 96-well plates and treated for 24 h with no additive (control) or 10 µM Mn (Mn-[2]C, Mn-TK, or Mn-BR) in complete medium. LDH release

was quantified with the LDH-Glo™ Cytotoxicity Assay (Promega, J2380) per the manufacturer's protocol. At 24 h, 5 µL of conditioned medium from each well was mixed with 50 µL LDH Storage Buffer in a white 96-well plate, followed by 50 µL LDH Detection Reagent; plates were incubated 30 min at room temperature (protected from light), and luminescence was recorded on a Cytation 5 multimode reader (BioTek). Each plate included background controls (medium only) and maximum-LDH controls (cells treated 15 min with the kit lysis solution). Background-subtracted signals were expressed as % LDH release and reported relative to the matched no-additive control. Data are mean ± SD (n = 3 independent experiments, each in technical triplicate). Statistical significance was assessed by one-way ANOVA with Dunnett's post-hoc test versus the no-additive control ($\alpha = 0.05$); significance: ns ($p \geq 0.05$), * ($p < 0.05$), ** ($p < 0.01$), *** ($p < 0.001$), **** ($p < 0.0001$).

6.14. DNA Damage Assessed by TUNEL Assay

U251-MG cells were seeded in 6-well plates in complete DMEM and allowed to adhere for 24 h at 37 °C, 5% CO_2. Cultures were then exposed for 3 h to no additive (control), $MnCl_2$, Mn-[2]C, Mn-TK, Mn-BR (10 µM Mn), or cisplatin (10 µM). Cells were fixed in 4% paraformaldehyde (PFA) in PBS for 15 min at room temperature, rinsed 3× with PBS, and permeabilized on ice for 2 min with 0.1% Triton X-100/0.1% sodium citrate in PBS. DNA fragmentation was labeled using the DeadEnd™ Fluorometric TUNEL System (Promega) according to the manufacturer's instructions. Nuclei were counterstained with DAPI (2 µg mL^{-1}, 5 min). Fluorescence images were acquired on an automated epifluorescence microscope under identical exposure and objective settings across conditions (9–16 non-overlapping fields per well). Images were processed with a single predefined pipeline (DAPI-based nuclear segmentation; TUNEL-positive mask by fixed threshold with size filtering), and DNA damages were expressed as % TUNEL-positive nuclei = ($TUNEL^+$ nuclei / total $DAPI^+$ nuclei) × 100. Field-level values were averaged per well, then across biological replicates (n = 3 independent experiments); data are mean ± SD. Statistical significance was assessed by one-way ANOVA with Dunnett's post-hoc test versus the no-additive control (α = 0.05).

6.15. Intracellular ROS Quantification and Lysosomal pH-dependence (H_2DCFDA ± NH_4Cl)

U251-MG cells were seeded in 6-well plates in complete DMEM and allowed to adhere for 24 h. Cultures were then exposed for 3 h to no additive (control), $MnCl_2$, Mn-[2]C, Mn-TK, Mn-BR or cisplatin (10 µM Metal). To assess lysosome-dependent contributions to ROS, a parallel set of wells was pre-incubated with ammonium chloride (NH_4Cl, 20–30 mM, 1 h) to neutralize lysosomal pH, them cells were treated 3 h with test compound. At the end of treatment, both adherent cells and floating cells were collected, washed once with PBS, and resuspended in PBS containing 10 µM H_2-DCFDA (Molecular Probes). After 30 min at 37 °C protected from light, cells were washed twice with PBS and resuspended in PBS for immediate analysis by flow cytometry (488 nm excitation, 530/30 nm emission). For each sample, 5×10^4 events were acquired after doublet discrimination and debris exclusion; dead-cell exclusion was performed by forward/side scatter. ROS was quantified as the mean fluorescence intensity and, in a confirmatory analysis, as the percentage of ROS-positive cells using a threshold defined from the 95th percentile of the no-additive control. Values were background-subtracted (unstained cells) and normalized to the matched no-additive control for each run. Data are reported as mean ± SD (n = 3 independent experiments) and analyzed per condition by one-way ANOVA with Dunnett's post-hoc test versus no-additive control ($\alpha = 0.05$); significance notation: ns ($p \geq 0.05$), * ($p < 0.05$), ** ($p < 0.01$), *** ($p < 0.001$), **** ($p < 0.0001$).

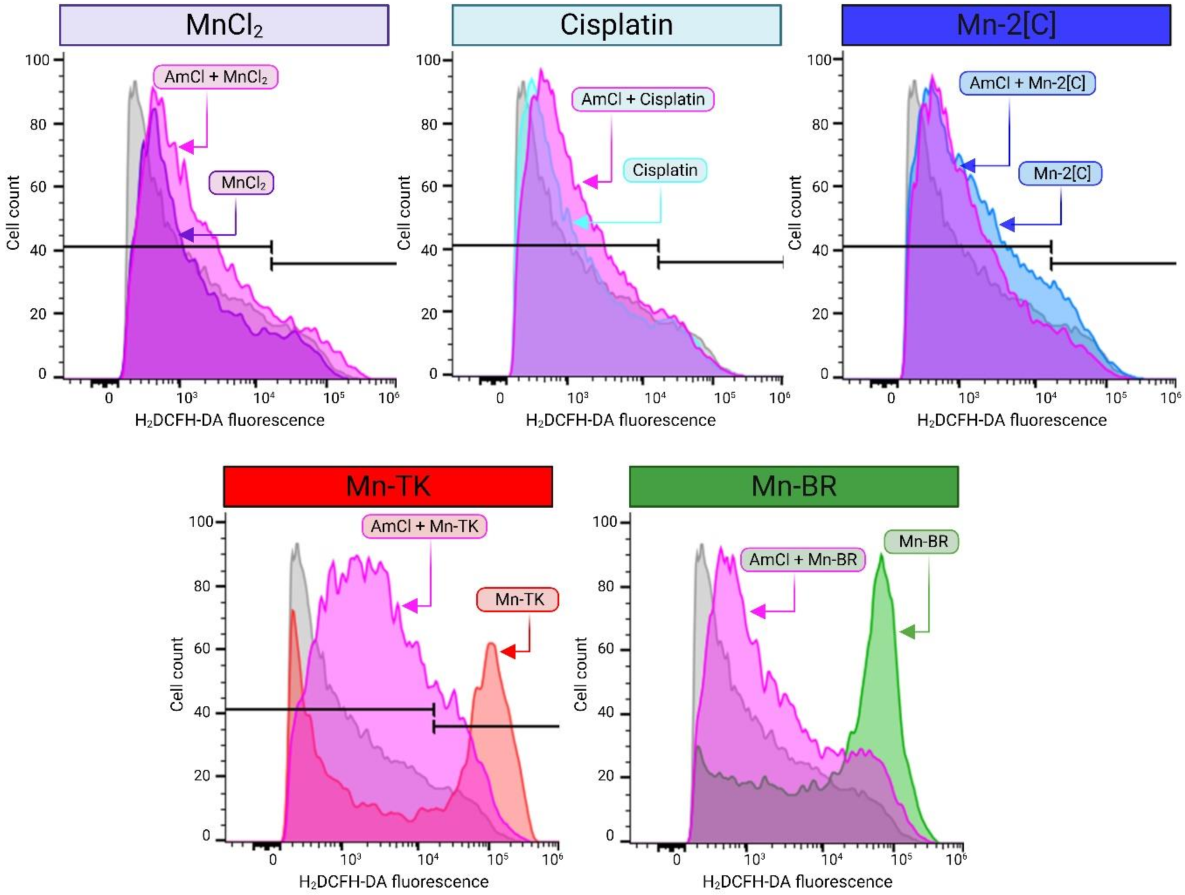

Figure S22. Reactive oxygen species (ROS) generation in U251-MG cells after 3 h exposure to Mn-TK, Mn-BR, Mn-[2]C, $MnCl_2$, or cisplatin (10 µM Metal), with or without ammonium chloride (NH_4Cl, 20 mM, 1 h) pretreatment to neutralize lysosomal pH. ROS levels were assessed by H_2DCFDA staining and quantified by flow cytometry, expressed as the percentage of ROS-positive cells relative to the no-additive control (mean ± SD, n = 3).

Table S5. Lysosome-dependent ROS readout in U251-MG cells (DCFH-DA). Cells were treated for 3 h with the indicated agents, with or without NH_4Cl pre-incubation (1 h) to neutralize lysosomal pH. Data are reported as ROS-positive cells (% of total, mean ± SD, n = 3).

Condition	ROS-positive cells (% of total), 3 h post-treatment	ROS-positive cells (% of total), +NH_4Cl pre-incubation (1 h) → 3 h treatment
Control	9.24 ± 3.06	9.24 ± 3.06

$MnCl_2$	12.8 ± 1.8	13.1 ± 0.7
Cisplatin	8.15 ± 2.65	12.54 ± 2.66
Mn-[2]C	11.9 ± 0.5	7.68 ± 0.34
Mn-TK	39.6 ± 11.4	16.25 ± 2.25
Mn-BR	46.65 ± 11.55	17.1 ± 0.93

6.16. Mitochondrial Membrane Potential (ΔΨm) Alterations

Mitochondrial membrane potential (ΔΨm) was assessed using the membrane-permeant cationic dye JC-1 (5,5′,6,6′-tetraethyl-benzimidazolylcarbocyanine iodide, Molecular Probes). In cells with intact ΔΨm, JC-1 accumulates in mitochondria and forms J-aggregates emitting red fluorescence. Upon mitochondrial depolarization, JC-1 remains in its monomeric form and emits green fluorescence.

U251-MG cells were seeded in 6-well plates in complete DMEM and allowed to adhere for 24 h. Cells were then treated with Mn-[2]C, Mn-TK, Mn-BR, or cisplatin at a final manganese (or equivalent drug) concentration of 10 μM for 3 h. For positive control of mitochondrial depolarization, cells were treated with H_2O_2 for 30 min under identical conditions.

Following treatment, both adherent and floating cells were collected to ensure inclusion of all cell populations. Cells were washed twice with PBS and resuspended in pre-warmed complete DMEM containing JC-1 (10 μg·mL^{-1}). Samples were incubated for 20 min at 37 °C in the dark.

After incubation, cells were washed once with PBS and resuspended in PBS supplemented with glucose for flow cytometric acquisition.

Fluorescence was analyzed using an Attune NxT flow cytometer equipped with a 488 nm excitation laser. Emission was collected in the green channel (530 ± 15 nm, FITC) for JC-1 monomers and in the red channel (585–590 nm, PE) for JC-1 aggregates. A minimum of 5×10^4 events per sample were acquired. Mitochondrial depolarization was quantified by calculating the

red/green fluorescence ratio. All experiments were performed in triplicate, and data are presented as mean ± SD.

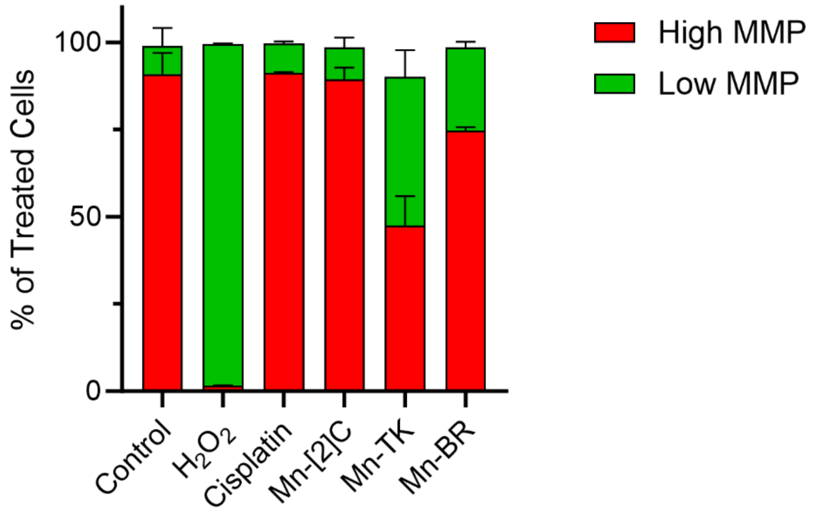

Figure S23. Quantitative analysis of mitochondrial membrane potential (ΔΨm) in U251-MG cells after 3 h treatment (10 µM). Mitochondrial membrane potential was assessed by JC-1 staining followed by flow cytometry. High MMP (red bars) represents polarized mitochondria with JC-1 aggregate fluorescence, while Low MMP (green bars) indicates depolarized mitochondria with JC-1 monomer fluorescence. Cells were treated with Mn-[2]C, Mn-TK, Mn-BR (10 µM), cisplatin (10 µM), or H_2O_2 (positive depolarization control). H_2O_2 induced near-complete mitochondrial depolarization, confirming assay sensitivity. Mn-TK and Mn-BR produced significant mitochondrial depolarization compared to control, whereas Mn-[2]C and cisplatin showed minimal changes at this early time point. Data represent mean ± SD from three independent experiments.

Table S6. Quantification of Mitochondrial Membrane Potential (ΔΨm) in U251-MG Cells After 3 h Treatment (10 µM). U251-MG cells were treated for 3 hours with Mn-[2]C, Mn-TK, Mn-BR (10 µM), cisplatin (10 µM), or H_2O_2 (positive depolarization control). Mitochondrial membrane potential was assessed by JC-1 staining followed by flow cytometry. High MMP corresponds to JC-1 red aggregates (polarized mitochondria), while Low MMP corresponds to JC-1 green monomers (depolarized mitochondria). Data are presented as mean ± SD from three

independent experiments. Statistical significance was determined relative to untreated control using one-way ANOVA with post hoc analysis (*$p < 0.05$, **$p < 0.01$, ***$p < 0.001$, ***$p < 0.0001$).

Condition	High MMP (%)	Low MMP (%)
Control	90.90 ± 6.00	8.04 ± 5.17
H_2O_2	1.57 ± 0.11 ****	97.90 ± 0.20 ****
Cisplatin	91.20 ± 0.20	8.50 ± 0.51
Mn-[2]C	89.35 ± 3.35	9.14 ± 2.86
Mn-TK	47.40 ± 8.50 ****	42.65 ± 7.75 ****
Mn-BR	74.70 ± 1.00 ***	23.75 ± 1.65 ***

6.17. Mitochondrial Bioenergetics Assessment by Seahorse XF96

U251-MG cells were seeded at 7,000 cells per well in poly-D-lysine-coated Seahorse XF96 microplates and allowed to adhere overnight at 37 °C, 5% CO_2. On the assay day, growth medium was replaced with Seahorse XF DMEM medium (Agilent, Cat. No. 103680-100]) supplemented with 10 mM glucose, 1 mM sodium pyruvate, and 2 mM L-glutamine (Agilent, Cat. No. 103680-100), and plates were equilibrated for 45–60 min in a non-CO_2 incubator at 37 °C.

The Seahorse XF Real-Time ATP Rate Assay (Agilent, Cat. No. 103592-100) was performed according to the manufacturer's protocol. Briefly, the assay measures the relative contribution of mitochondrial oxidative phosphorylation (OXPHOS) and glycolysis to cellular ATP production. Sequential injections of oligomycin and rotenone/antimycin A were applied to determine ATP production rates linked to mitochondrial and glycolytic activity. OCR (oxygen consumption rate) and ECAR (extracellular acidification rate) were recorded in real time using the Seahorse XFe96 metabolic analyzer, following cycles of mixing (3 min), waiting (0 min), and measuring (3 min). Cells were treated with Mn-[2]C, Mn-TK, or Mn-BR (10 µM Metal) for 24 h prior to the assay.

Table S7. Seahorse XF Real-Time ATP Rate Assay in U251-MG cells treated with Mn-based compounds. Cells were treated with Mn-[2]C, Mn-TK, or Mn-BR (10 µM Mn) for 24 h.

Group	Glycolysis (pmol/min)	Mito ATP (pmol/min)	Total ATP (pmol/min)	% Glycolysis	% OXPHOS
Control	264.0	128.4	392.4	67.5%	32.5%
Mn-[2]C	302.0	92.9	395.0	76.4%	23.6%

Mn-TK	294.8	146.9	441.7	66.7%	33.3%
Mn-BR	245.9	151.1	397.0	61.9%	38.1%

6.18. Cell Growth Analysis

U251-MG cells were seeded at a density of 1,000 cells per well in 96-well plates and treated with Mn-[2]C, Mn-TK, or Mn-BR, each at a final manganese concentration of 10 µM. Following treatment, cells were incubated under standard conditions (37 °C, 5% CO_2), and cell proliferation was monitored in real time using the Lionheart FX Automated Microscope (BioTek).

Brightfield images were acquired every 4 hours over a 72-hour period using a 4× objective lens. Image analysis and cell count quantification were performed using Gen5™ Image Analysis Software (BioTek), enabling the generation of growth curves for each condition.

Growth rates were calculated using GraphPad Prism software by fitting data to an exponential (Malthusian) growth model. The model yielded growth rate constants (k) and doubling times (DT) for each treatment group. The goodness of fit for each curve was assessed using the R^2 value.

All experiments were performed in triplicate, and data are presented as mean ± standard error of the mean (SEM). Statistical comparisons between groups were conducted to evaluate the impact of different Mn-based structures on cell proliferation.

These results support the observed therapeutic potential of Mn-[2]C, Mn-TK, and Mn-BR, highlighting treatment-specific differences in growth inhibition profiles.

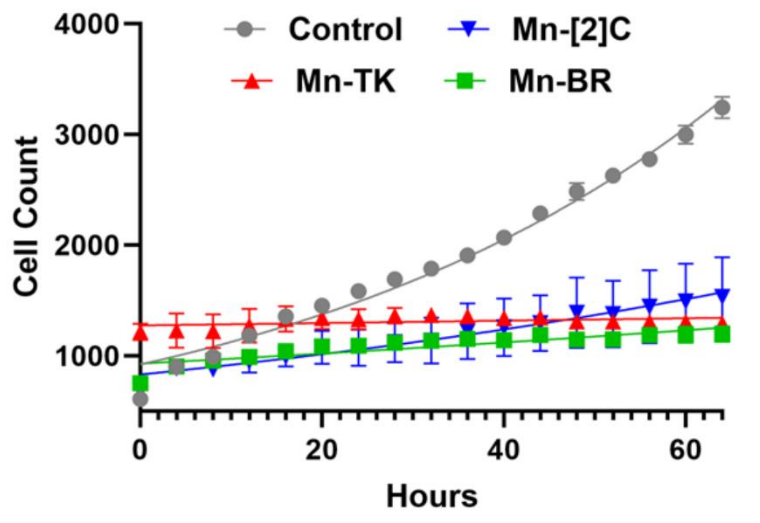

Figure S24. Growth curves of U251-MG cells over 72 h at 37 °C under control conditions or after exposure to Mn-[2]C, Mn-TK, or Mn-BR (10 µM Mn). Cell counts were recorded at defined intervals and normalized to the t_0 value; data are mean ± SD (n = 3).

Table S8. Population Doubling Times (PDTs) for Control, Mn-[2]C, Mn-TK, or Mn-BR Treated Cells. This table presents the population doubling times (PDTs) for U251-MG cell lines over a three-day period. PDTs, calculated from the slope of the natural log of cell numbers plotted against time, indicate the time required for the cell population to double during the early log growth phase. The data compares PDTs between control groups and cells treated with 10 µM of Mn-[2]C, Mn-TK, or Mn-BR.

	Control	MnBR	MnTK	Mn2C
Exponential (Malthusian) growth				
Best-fit values				
Y0	921.2	929	1274	830.7
k	0.02	0.004678	0.0008274	0.009975
DoublingTime	34.66	148.2	837.8	69.49
95% CI (profile likelihood)				
Y0	877.2 to 966.1	884.0 to 974.9	1201 to 1349	678.0 to 994.5
k	0.01902 to 0.02100	0.003478 to 0.005883	-0.0006938 to 0.002350	0.005683 to 0.01443
DoublingTime	33.01 to 36.45	117.8 to 199.3	294.9 to +infinity	48.02 to 122.0

7. *In vivo* Biological Studies

All animal experiments were conducted in accordance with the policies of the New York University (NYU) and the University of Utah (UU) Institutional Animal Care and Use Committees (IACUC). Athymic NU/J nude mice (4-6 weeks old, about 20 g) were fed under normal conditions of 12 h light and dark cycles and given access to food and water *ad libitum*. All animal experimentation was approved by the Institutional Animal Care and Use Committee of NYUAD and UU.

7.1. Biodistribution Study on Healthy Animals

The mice were divided randomly into groups (n=20). They were administered 0.2 mL of either saline (control) or one of the Manganese complexes (Mn-TK and Mn-BR) at a dose of 0.05 mmol-Mn/kg via intraperitoneal injection.

At predetermined time points—4 hours, 24 hours, 72 hours, and one week post-injection—5 mice per group were euthanized, and T_1-weighted spin echo MR imaging was conducted using a 3.0 T clinical MRI to assess the distribution of these compounds in the liver, kidney, and brain tissues. Following MRI analysis, the major organs (liver, kidney, brain, heart, lung, and spleen) were harvested to measure the manganese concentration per gram of tissue in each organ using ICP-MS, allowing for a comparative study of the biodistribution over time.

7.1.1. Quantitative T_1-Mapping with Spin echo MR Imaging

Postmortem mice were packed in sealed bags and placed in a transmit/receive quadrature knee coil within a clinical 3T MRI scanner (MAGNETOM Prisma, Siemens Healthineers, Erlangen, Germany) for quantitative T_1 mapping. A saturation recovery spin echo sequence was employed with the following parameters: variable repetition times (TR, in ms) of 150, 200, 300, 500, 800, 1000, 1500, 3000, 4000, and 8000 ms; echo time (TE) of 11 ms; field of view (FOV) of 130 mm × 130 mm; acquisition matrix of 320 × 320; and slice thickness of 3.5 mm.

The acquired data was processed using MATLAB 2024a (MathWorks Inc.) with a custom script for pixel-by-pixel nonlinear least squares fitting (utilizing the lsqcurvefit function). The signal at each voxel was plotted against its corresponding TR, and a T_1 recovery curve was fit using the $M(TR) = M_0\left(1 - e^{-TR/T_1}\right)$. The two unknowns,$M_0$ and T_1, were fitted for each voxel. T_1 values were reported in milliseconds (ms).

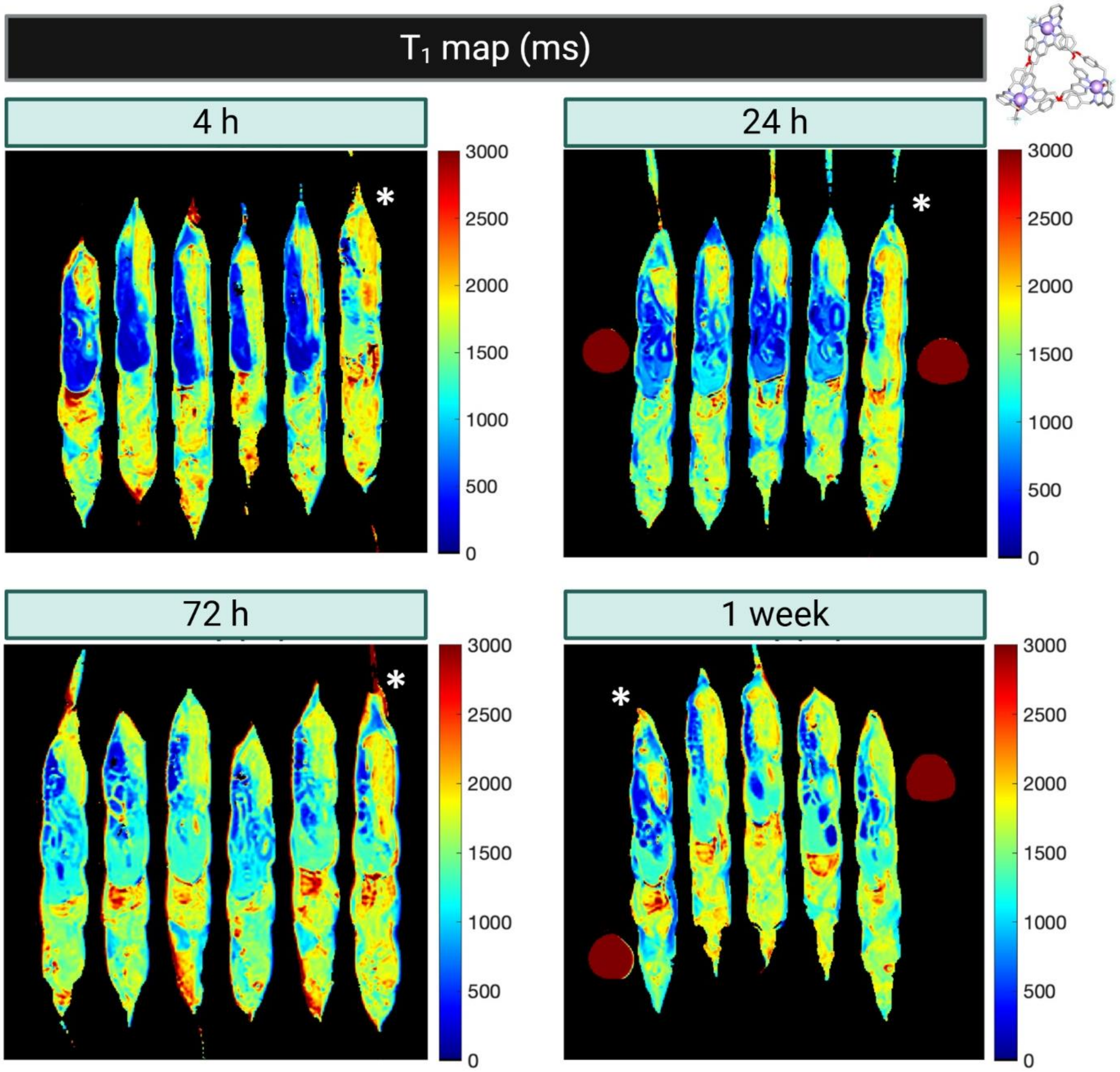

Figure S25. Post-mortem T_1-maps displaying full-body contrast enhancement in mice at 4 hours, 24 hours, 72 hours, and one week post-injection. Mice were administered Mn-TK at a dose of

0.05 mmol-Mn/kg via intraperitoneal injection. The images illustrate the temporal distribution of the contrast agent, with the control mouse (saline injected) denoted by a white asterisk.

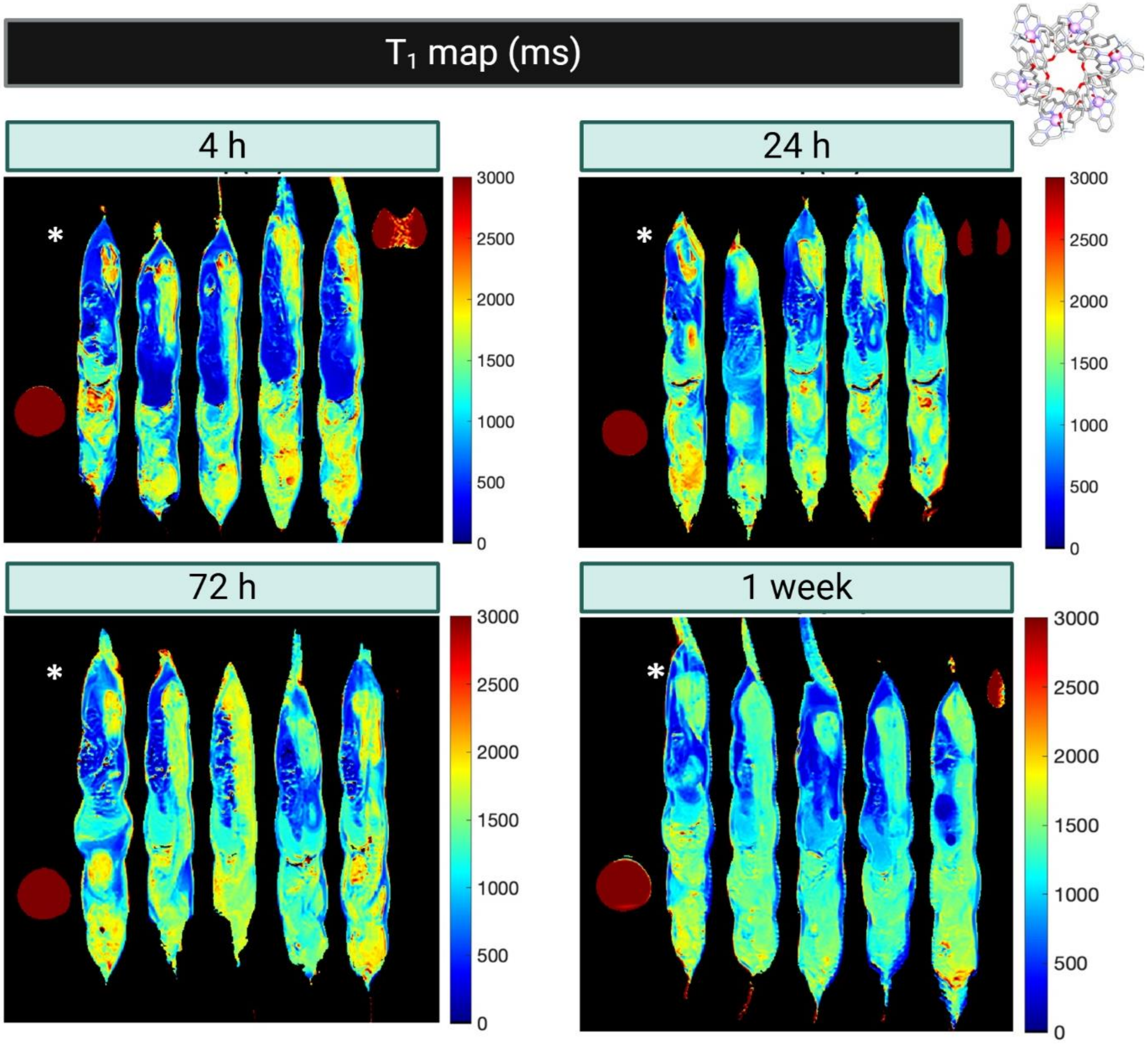

Figure S26. Post-mortem T_1-maps displaying full-body contrast enhancement in mice at 4 hours, 24 hours, 72 hours, and one week post-injection. Mice were administered Mn-BR at a dose of 0.05 mmol-Mn/kg via intraperitoneal injection. The images illustrate the temporal distribution of the contrast agent, with the control mouse denoted by a white asterisk.

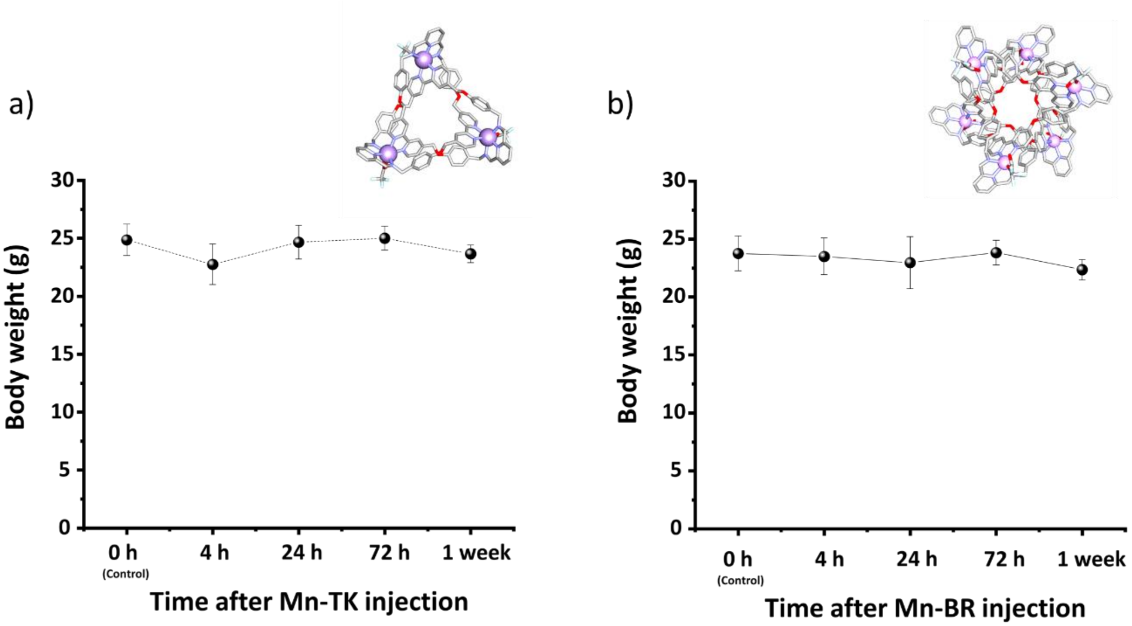

Figure S27. Body weight of healthy animals at baseline (t=0) and up to one week treated with a) Mn-TK, and b) Mn-BR at a dose of 0.05 mmol-Mn/kg via intraperitoneal injection (n=5), indicating no significant change in body weight post-treatment within the observation period.

7.1.2. Manganese Determination in Tissue

Manganese biodistribution in the major organs (liver, kidney, brain, heart, lung, and spleen) via ICP-MS was performed on an Agilent 7800 series ICP-MS. Mouse tissues (n = 5, <100 mg wet weight each sample) were dried at 95°C in a vacuum oven. Dried 5 to 10 mg samples were weighed into Pyrex tubes and digested in 1.5 mL concentrated HNO_3 at 110 °C for 4 h. Each sample was diluted as optimally with 18 MOhm water to prevent anomalously high readings. Manganese concentrations within the cells were subsequently measured using an Agilent 7800 series ICP-MS, and Tissue concentrations were determined as Mn µg/g dry tissue weight.

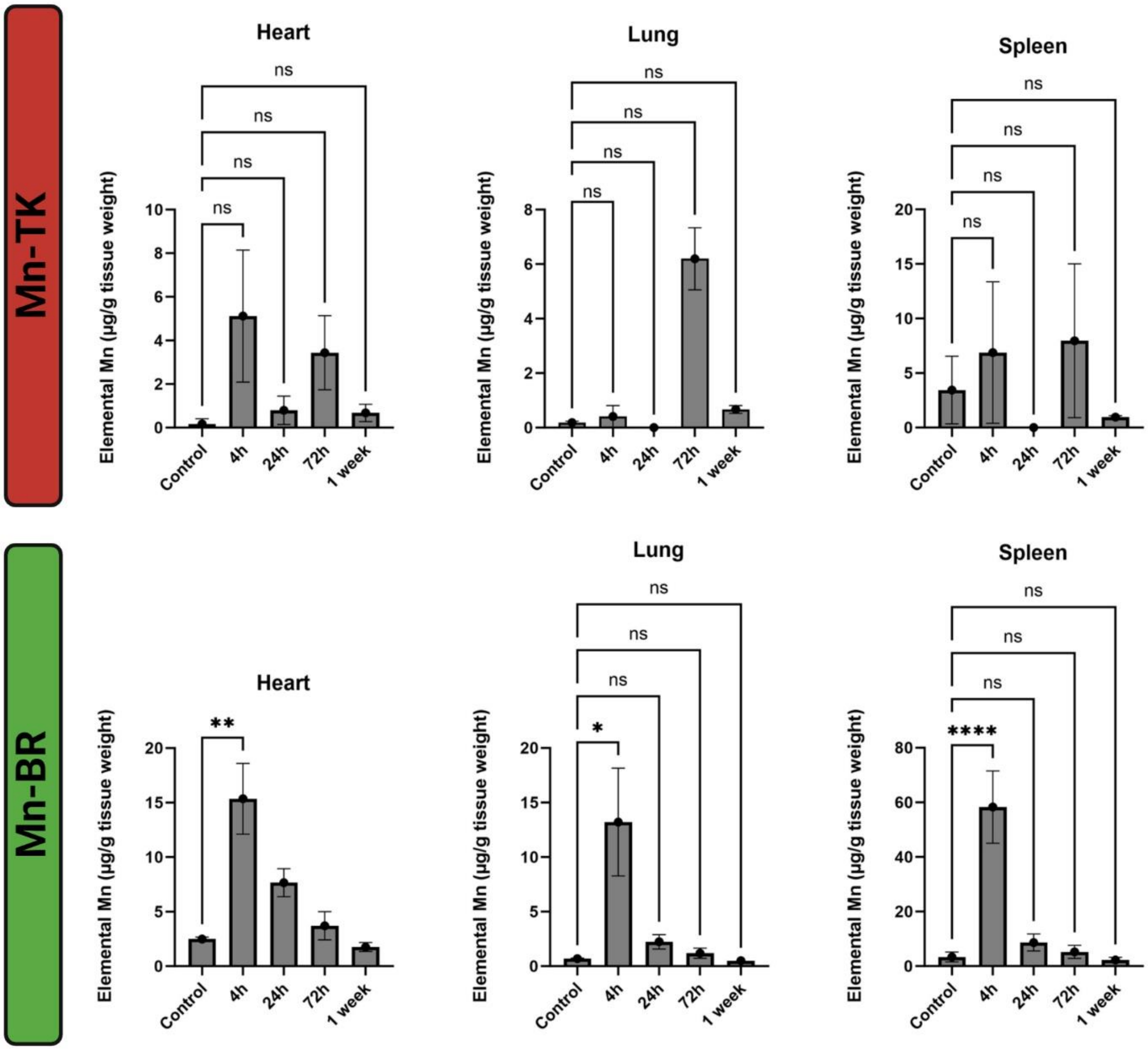

Figure S28. Manganese levels in the heart, lung, and spleen post-treatment with Mn-TK and Mn-BR, demonstrating the metabolism and clearance patterns of these metal-organic structures

7.2. In Vivo Biosafety Evaluation

7.2.1. Acute Toxicity Study and Body-Weight Monitoring

An acute toxicity study was conducted in healthy male CD-1 mice (6–8 weeks old, n = 3 per group). Animals were housed under standard laboratory conditions with free access to food and water and maintained under a 12 h light/dark cycle. All experimental procedures were performed in accordance with institutional animal care and use guidelines.

Mn-TK or Mn-BR were administered as a single intravenous injection at a dose of 0.10 mmol-Mn·kg^{-1} body weight. This dose corresponds to four times the therapeutic dose used in tumor studies and two times the imaging dose. Control animals received an equivalent volume of saline.

Following administration, animals were monitored daily for seven consecutive days for survival, general health status, behavioral changes, grooming behavior, posture, mobility, and signs of injection-site irritation. Body weight was recorded prior to injection (baseline) and daily throughout the 7-day observation period using a calibrated analytical balance. Body-weight changes were calculated relative to baseline values. A weight loss exceeding 15% of initial body weight was predefined as a humane endpoint criterion.

At the 7-day endpoint, animals were euthanized and blood and major organs were collected for biochemical and histopathological analyses.

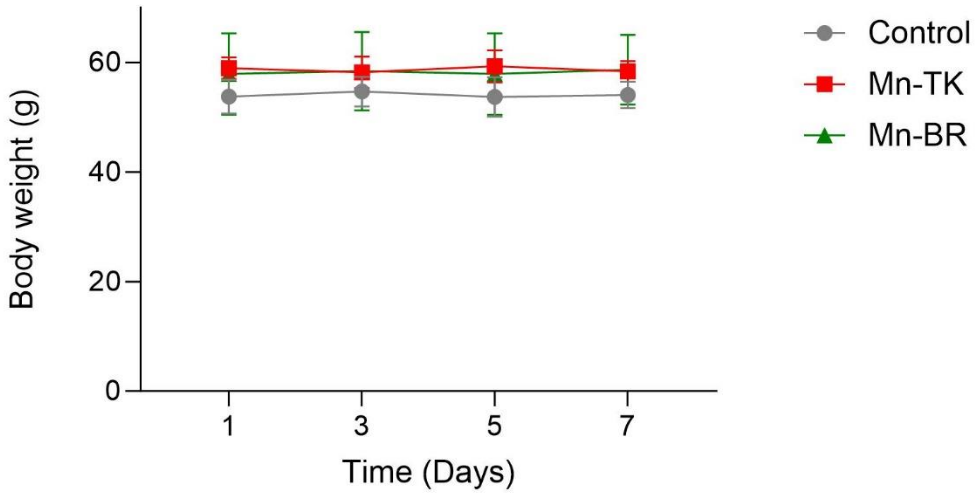

Figure S29. Body weight monitoring during the acute toxicity study of Mn-TK and Mn-BR. Body weight changes of mice treated with saline (control), Mn-TK, or Mn-BR following single-dose administration and monitored over a 7-day period. No statistically significant differences in body weight were observed among the three groups at any time point, indicating good acute tolerability of both materials. Data are presented as mean ± SD.

7.2.2. Blood Biochemical Analysis

At the endpoint of the acute toxicity study, whole blood was collected by cardiac puncture under anesthesia and allowed to clot at room temperature. Samples were centrifuged at 3,000 rpm for 10 min to obtain serum, which was used for biochemical analysis.

Serum biochemical parameters were measured to evaluate hepatic and renal function. The following biomarkers were quantified: total bilirubin, total protein, albumin, aspartate aminotransferase (AST), alanine aminotransferase (ALT), alkaline phosphatase (ALP), gamma-glutamyl transferase (GGT), urea, and creatinine.

Biomarker quantification was performed using the Beckman Coulter AU480 clinical chemistry analyzer (Beckman Coulter) employing standard automated spectrophotometric methods. All measurements were validated according to established standard operating procedures using calibration standards and multi-level quality control (QC) materials to ensure analytical accuracy and precision.

The analyzed liver biomarkers included total bilirubin, total protein, albumin, aspartate aminotransferase (AST), alanine aminotransferase (ALT), and alkaline phosphatase (ALP), together with renal function markers urea and creatinine.

Across all groups, serum biochemical values remained within physiological ranges, with no evidence of treatment-related hepatic dysfunction. Total bilirubin levels were low in control and treated animals (control: 0.08 ± 0.06 $mg.dL^{-1}$; Mn-TK: 0.17 ± 0.05 $mg.dL^{-1}$; Mn-BR: 0.13 ± 0.03 $mg.dL^{-1}$), indicating the absence of hepatobiliary injury or cholestasis. Markers of hepatic synthetic function, including total protein and albumin, were comparable among groups, with no consistent decrease suggestive of impaired liver function.

Transaminase activities showed moderate inter-animal variability, a common feature in tumor-bearing nude mice, but no systematic elevation associated with Mn-TK or Mn-BR treatment. Mean AST values were 178.8 ± 83 $IU.L^{-1}$ in controls, 162.3 ± 32 $IU.L^{-1}$ in Mn-TK-treated mice, and 205.6 ± 201 $IU.L^{-1}$ in Mn-BR-treated mice, while ALT values remained within a narrow range across groups (35.6 ± 2, 37.4 ± 1.6, and 42.4 ± 5.1 $IU.L^{-1}$ for control, Mn-TK, and Mn-BR, respectively). No treatment-dependent trend indicative of hepatocellular injury was observed.

Markers of cholestatic injury further supported the absence of liver toxicity. ALP values did not display treatment-related increases, and γ-glutamyl transferase (GGT) levels remained below the detection limit (< 5 $IU.L^{-1}$) in all animals.

Renal function was likewise preserved following Mn-TK and Mn-BR administration. Serum creatinine levels were tightly clustered across all groups (0.11–0.13 $mg.dL^{-1}$), and although urea values exhibited modest variability, mean levels were comparable between control and treated mice (48.7 ± 11, 51.4 ± 6, and 53.9 ± 12 $mg.dL^{-1}$ for control, Mn-TK, and Mn-BR, respectively), with no evidence of nephrotoxicity.

Overall, the extent of biochemical variability observed in Mn-TK- and Mn-BR-treated animals was comparable to that of control animals, and no consistent or treatment-specific abnormalities were detected. These results demonstrate that both Mn-TK and Mn-BR exhibit a favorable acute biosafety profile at the 7-day endpoint, with preserved hepatic and renal function following single-dose administration.

Table S9. Serum biochemical parameters following Mn-TK and Mn-BR treatment. Data are presented as mean ± SD. Statistical significance is indicated where applicable.

Parameter	Control	Mn-TK	Mn-BR
**Total bilirubin ($mg.dL^{-1}$)**	0.08±0.06	0.17±0.05	0.13±0.03
**Urea ($mg.dL^{-1}$)**	48.7±11	51.4±6	53.9±12
**Total protein ($g.dL^{-1}$)**	4.9±0.11	5.29±1.03	5.04±0.15
**Albumin ($g.dL^{-1}$)**	2.62±0.13	2.99±0.69	2.82±0.11
**AST ($IU.L^{-1}$)**	178.8±83	162.3±32	205.6±201

**ALT (IU.L^{-1})**	35.6±2	37.35±1.6	42.4±5.1
**ALP (IU.L^{-1})**	36.4±10.9	24.7±5.3	47.5±11.6
**GGT (IU.L^{-1})**	<5	<5	<5
**Creatinine (mg.dL^{-1})**	0.12±0.004	0.13±0.02	0.11±0.009

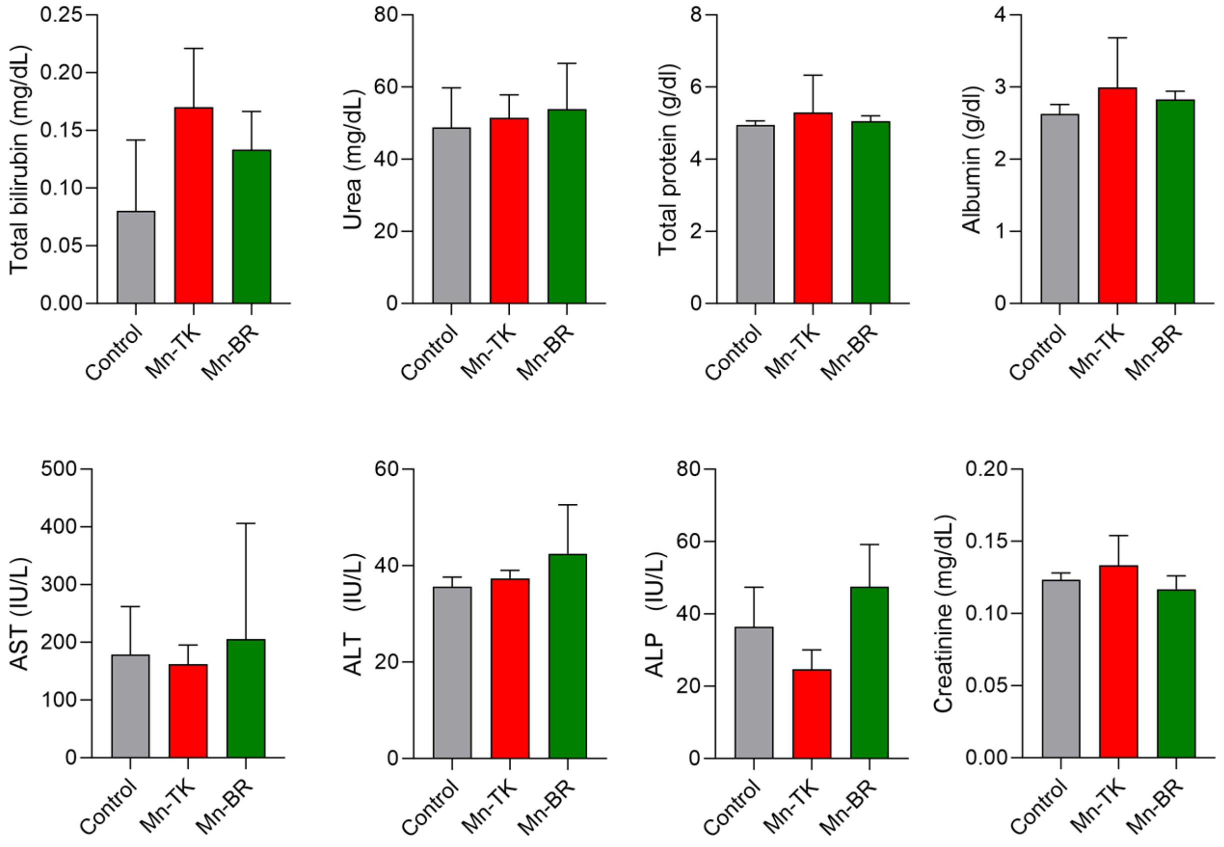

Figure S30. Blood biochemical analysis at the 7-day endpoint of the acute toxicity study. Serum biochemical parameters measured in control, Mn-TK–treated, and Mn-BR–treated nude mice (*n* = 3 per group) at the 7-day endpoint following single-dose administration (0.10 mmol/kg). Liver function markers (total bilirubin, total protein, albumin, AST, ALT, and ALP) and renal function markers (urea and creatinine) remained within physiological ranges across all groups. No treatment-related elevations indicative of hepatic or renal toxicity were observed for Mn-TK or Mn-BR. Notably, AST levels displayed high inter-animal variability in the control group, whereas Mn-TK and Mn-BR groups showed lower and more consistent values. Data are presented as mean ± SD. Statistical significance is indicated where applicable.

7.2.3. Histopathological Examination (Hematoxylin and Eosin Staining)

At the endpoint of the acute toxicity study, major organs including the heart, liver, kidney, and spleen were harvested immediately following euthanasia. Tissues were gently rinsed in cold phosphate-buffered saline (PBS) to remove residual blood and subsequently fixed in 10% neutral-buffered formalin for 24–48 h at room temperature. After fixation, samples were dehydrated through a graded ethanol series, cleared, and embedded in paraffin blocks according to standard histological procedures.

Paraffin-embedded tissues were sectioned into 4–5 µm thick slices using a microtome and mounted onto glass slides. Sections were stained with hematoxylin and eosin (H&E) following routine staining protocols. Stained sections were examined under bright-field microscopy by trained personnel blinded to treatment groups for assessment of tissue architecture, cellular morphology, inflammatory infiltration, necrosis, and structural abnormalities. Representative images were acquired at appropriate magnifications.

In control animals, all organs displayed normal tissue architecture and cellular morphology. Importantly, organs from Mn-TK– and Mn-BR–treated mice showed no discernible histopathological abnormalities when compared with controls.

Cardiac tissue exhibited intact myocardial fiber organization without evidence of inflammation or degeneration. Renal sections showed preserved glomerular and tubular structures, with no signs of tubular necrosis or interstitial damage. Liver sections maintained normal lobular architecture, with well-organized hepatocytes and no detectable inflammation, steatosis, or necrotic lesions. Splenic tissue retained intact red and white pulp organization, with no evidence of lymphoid depletion or abnormal infiltration. These findings confirm the absence of off-target organ damage following Mn-TK or Mn-BR administration.

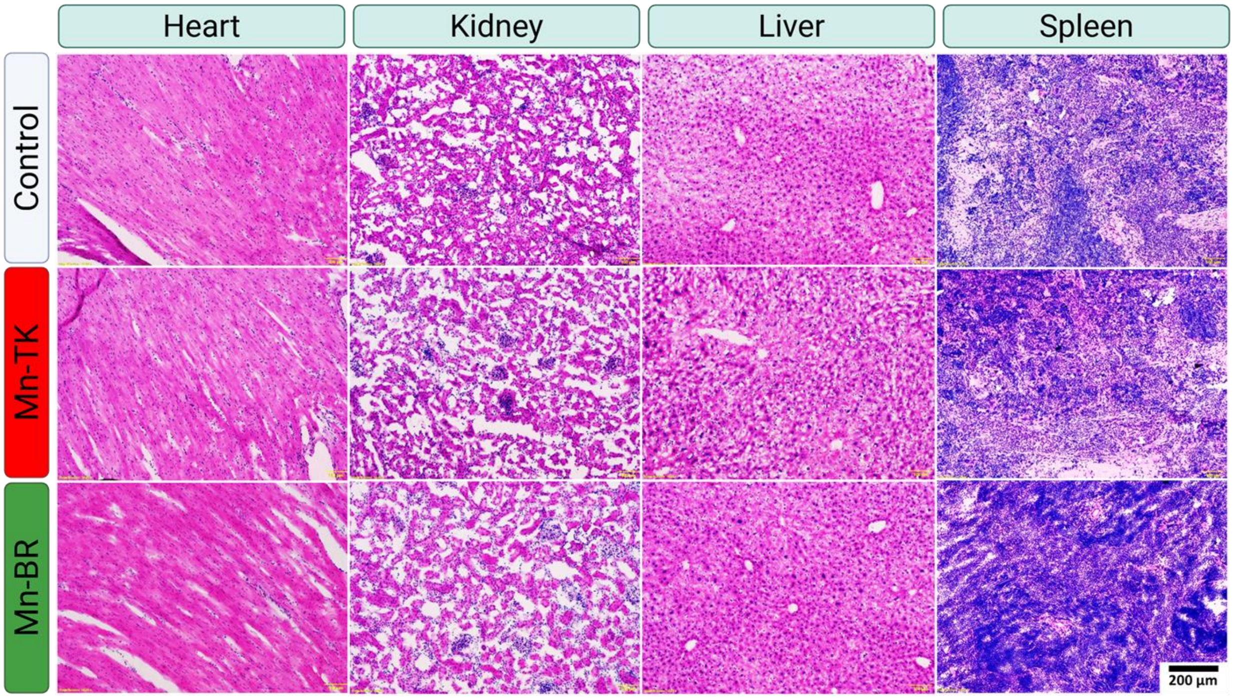

Figure S31. Histopathological evaluation of major organs following Mn-TK and Mn-BR administration. Representative H&E-stained sections of the heart, kidney, liver, and spleen collected from control, Mn-TK–treated, and Mn-BR–treated mice at the endpoint of the acute toxicity study (0.10 mmol/kg). All examined organs from Mn-TK and Mn-BR groups display preserved tissue architecture and normal cellular morphology comparable to control animals, with no evidence of inflammation, necrosis, degeneration, or structural abnormalities. These results indicate the absence of off-target organ toxicity following Mn-TK or Mn-BR administration. Scale bar: 200 µm.

7.3. Tumor Model Establishment

The U251-MG cancer model was employed as an example of aggressive brain cancer to evaluate the therapeutic effect of Mn-TK and Mn-BR. 5×10^6 U251-MG cells in 200 µL of DMEM medium were injected subcutaneously into the right axillary region of nude mice. The mice were utilized in subsequent experiments after the tumor size had reached approximately 50-100 mm^3.

7.4. Biodistribution Study on Tumor-bearing Mice

The tumor-bearing mice were divided randomly into groups and administered 0.2 mL of either saline (control) or one of the manganese structures (Mn-TK and Mn-BR) at a dose of 0.05 mmol-Mn/kg via intraperitoneal injection. At predetermined time points—24 hours and 96 hours post-injection—5 mice per group were euthanized.

Subsequently, T_1-weighted spin-echo MR imaging was conducted using a 3.0 T clinical MRI to assess the distribution of these compounds in the tumor, liver, kidney, and brain tissues. Following MRI analysis, the major organs and tumors were harvested to measure the manganese concentration per gram of tissue in each organ using ICP-MS, allowing for a comparative study of the biodistribution over time.

Table S10. T_1 Relaxation Times (ms) in Organs after Mn-TK and Mn-BR Administration. Values represent mean ± SD (n = 3). Measurements performed at 3T, 20 °C.

Condition	Tumor	Liver	Brain	Kidney
Control	2435.5 ± 69	1690.0±15	2191 ± 82	2258 ± 112
Mn-TK 24 h	2018.8±11	1413.0 ± 90	2026 ± 143	1592 ± 232
Mn-TK 96 h	1807.3 ± 328	1620.5 ± 28	1951 ± 127	1984 ± 72
Mn-BR 24 h	1980.0± 10	921.1 ± 256	2184 ± 128	1289 ± 434
Mn-BR 96 h	1912.0 ± 158	1407.5 ± 28	2086 ± 91	1817 ± 64

Table S11. Elemental Mn Concentrations (μg/g Tissue) in Organs after Mn-TK and Mn-BR Administration. Values represent mean ± SD (n = 3). Quantification performed by ICP-MS.

Condition	Liver	Kidney	Heart	Lung	Spleen	Brain	Tumor
Control	2.37 ± 1.74	8.84 ± 2.93	0.087 ± 0.023	0.092 ± 0.209	3.44 ± 3.11	0.54 ± 0.53	0.087 ± 0.023
Mn-TK 24 h	12.63 ± 2.31	11.75 ± 4.48	0	0	9.00 ± 1.28	0.62 ± 0.47	11.06 ± 1.11
Mn-TK 96 h	3.92 ± 0.39	3.31 ± 0.36	0	0	3.69 ± 0.30	0.99 ± 0.99	8.21 ± 0.82
Mn-BR 24 h	8.95 ± 0.70	5.36 ± 0.87	0.50 ± 0.31	0.78 ± 0.38	10.48 ± 1.64	0.68 ± 0.02	12.97 ± 0.53
Mn-BR 96 h	4.52 ± 0.59	3.63 ± 0.71	0	0	2.45 ± 0.18	0.90 ± 0.12	9.05 ± 0.55

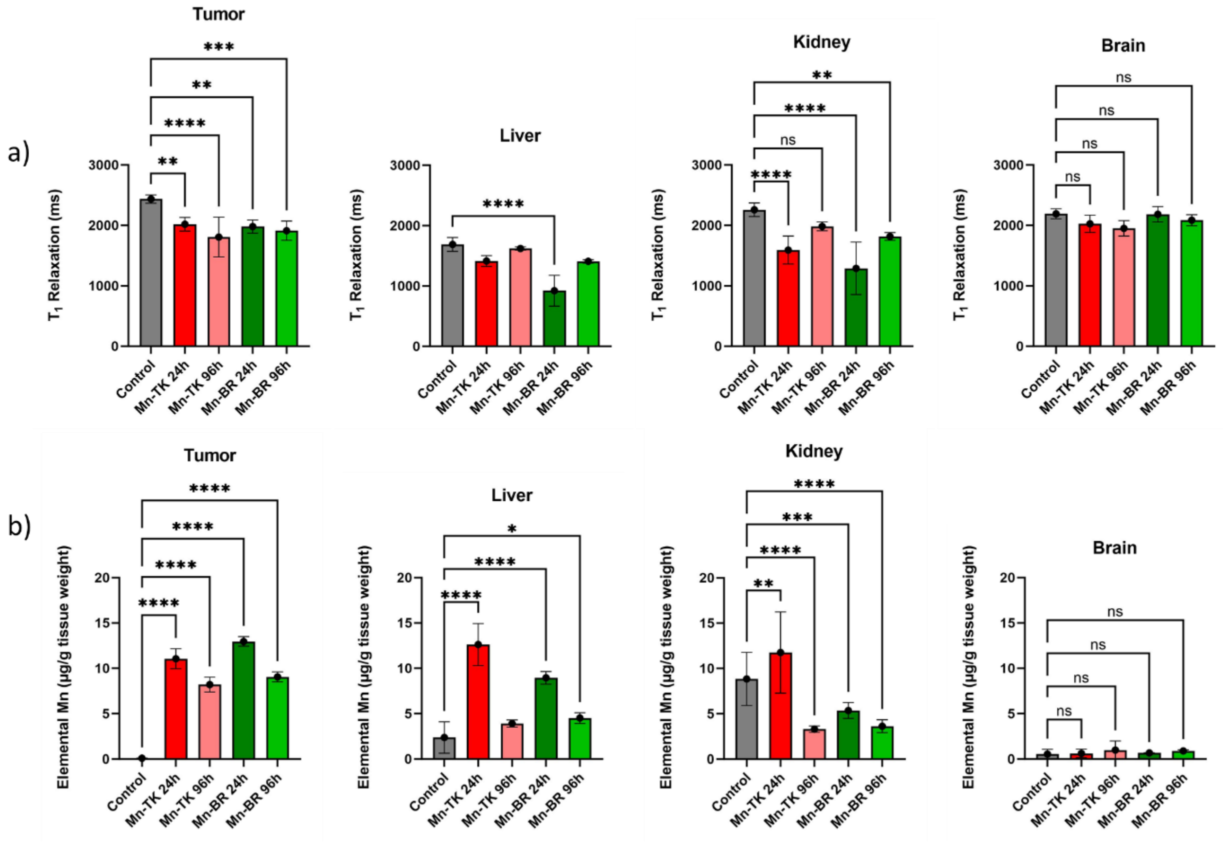

Figure S32. Ex vivo T_1 and Manganese biodistribution after a single IP dose (0.05 mmol Mn·kg^{-1}) in tumor bearing mice. a) T_1 (ms) and b) ICP-MS Mn (µg·g^{-1}) in tumor, liver, kidney, and brain at 24 h and 96 h of animal treated with Mn-TK (red) and Mn-BR (green). Data are mean ± SD (n = 3); one-way ANOVA with post-hoc tests; ns, p<0.05, *p<0.01, **p<0.001, ***p<0.0001.

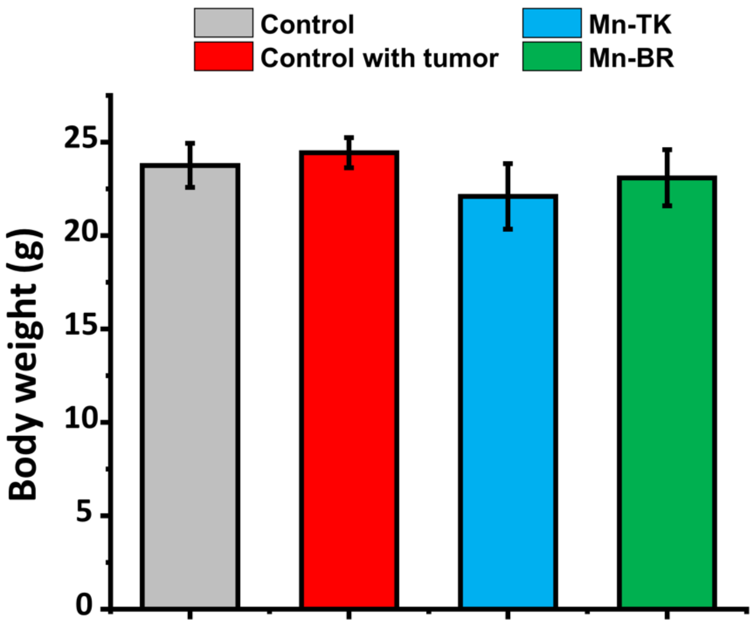

Figure S33. Body weight of tumor-bearing mice at baseline (t=0) and after 24 hours, indicating no significant change in body weight post-treatment within the 24-hour observation period.

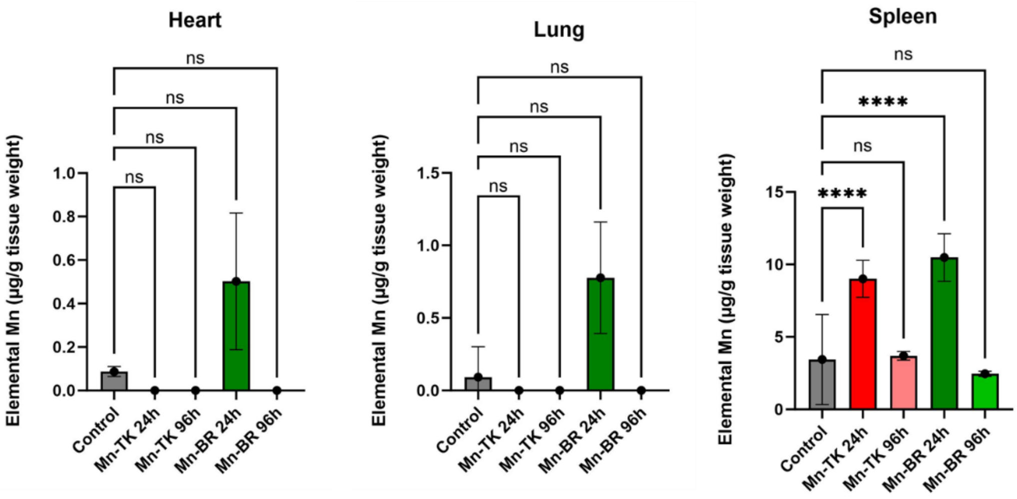

Figure S34. Manganese levels in heart, lung and spleen in tumor-bearing mice following treatment with Mn-TK (red) and Mn-BR (green) complexes at a dose of 0.05 mmol-Mn/kg via intraperitoneal injection. Data points represent measurements at 24 hours (solid colors) and 96 hours (lighter colors) post-injection.

7.5. *In vivo* Antitumor Efficacy

To evaluate the antitumor efficacy, tumor-bearing mice were randomized into three groups: Control, Mn-TK, and Mn-BR (n=5 per group). Each group received an intraperitoneal injection of either 200 µL of saline (Control), Mn-TK, or Mn-BR at a dosage of 0.025 mmol-Mn/kg (200 µL), administered every two days over 13 days.

Animal weight and tumor volume were measured before each injection. Tumor size was monitored via caliper measurement every 2 days, and the tumor volume was estimated using *Equation (1)*:

$$V=0.5\times length\times(width)^2 \quad (1)$$

On day 13, the mice were sacrificed, and the tumor masses were harvested, weighed, and photographed.

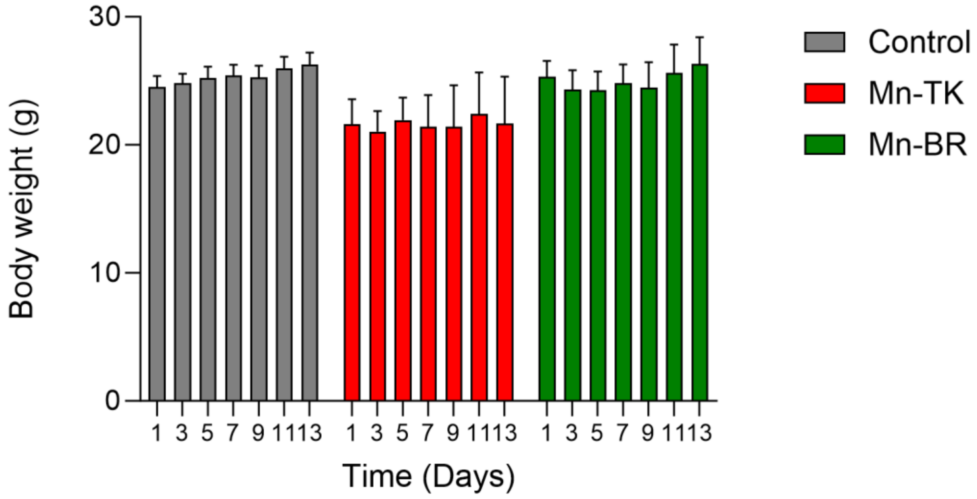

Figure S35. Body-weight monitoring during Mn-TK and Mn-BR treatment. Body-weight changes of mice treated with saline (control), Mn-TK, or Mn-BR over the course of the experiment (days 1–13). Body weights remained stable in all groups throughout the study, and no statistically significant differences were observed between Mn-TK- or Mn-BR-treated mice and controls at

any time point, indicating good tolerability of both materials and the absence of treatment-related weight loss. Data are presented as mean ± SD.

7.6. In Vivo Tumor Apoptosis Analysis by TUNEL Staining

At the conclusion of the therapeutic efficacy study (day 14), tumors were excised immediately following euthanasia and processed for apoptosis analysis. Tumor tissues were rinsed in cold phosphate-buffered saline (PBS) to remove residual blood and fixed in 4% paraformaldehyde at 4 °C for 24 h. Following fixation, samples were cryoprotected in 30% sucrose solution until complete infiltration, embedded in optimal cutting temperature (OCT) compound, and frozen for cryosectioning.

Tumor cryosections (8–10 µm thickness) were prepared using a cryostat and mounted onto glass slides. Apoptotic DNA fragmentation was detected using a terminal deoxynucleotidyl transferase dUTP nick end labeling (TUNEL) assay kit according to the manufacturer's protocol. Briefly, tissue sections were permeabilized, incubated with terminal deoxynucleotidyl transferase enzyme and labeled nucleotides to allow incorporation at sites of DNA strand breaks, and subsequently washed to remove unbound reagents.

Cell nuclei were counterstained with 4',6-diamidino-2-phenylindole (DAPI) to visualize total nuclear content. Sections were mounted using antifade mounting medium and imaged using a laser-scanning confocal microscope under identical acquisition settings for all treatment groups. TUNEL-positive nuclei were identified by red fluorescence, while total nuclei were visualized by DAPI staining (blue). Representative images were acquired from multiple tumor regions per sample to ensure reproducibility.

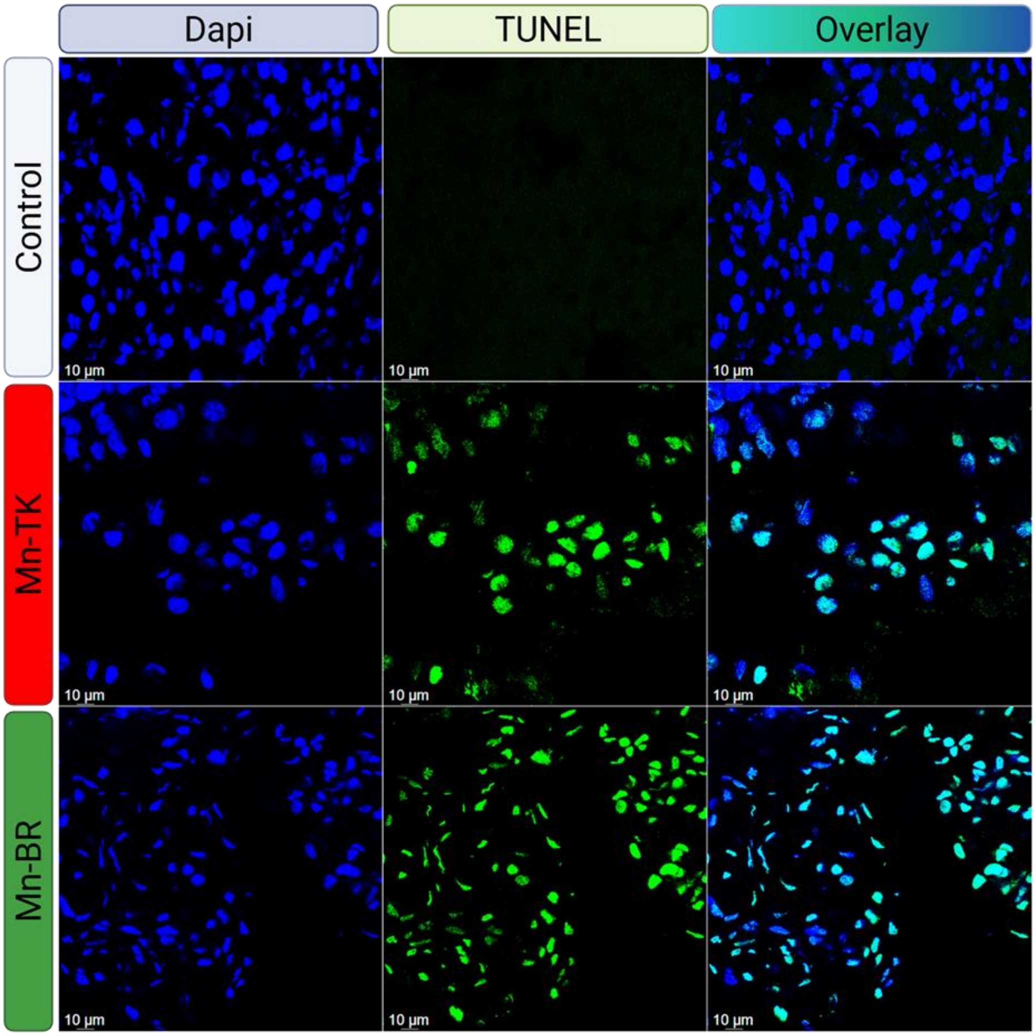

Figure S36. In vivo apoptosis analysis of tumors following Mn-TK and Mn-BR treatment. TUNEL staining of tumor cryosections collected after 14 days of treatment showing apoptotic DNA fragmentation in vivo. DAPI (blue) marks cell nuclei, TUNEL (red) indicates apoptotic nuclei, and merged images show colocalization of TUNEL signal with nuclear staining. Control tumors exhibit minimal TUNEL signal, indicating low basal apoptosis. In contrast, Mn-TK–treated tumors show a substantial increase in TUNEL-positive nuclei distributed throughout the tumor tissue. Mn-BR–treated tumors display the strongest apoptotic response, with extensive regions of TUNEL positivity, confirming robust apoptosis induction. These results demonstrate that Mn-TK and Mn-BR exert their antitumor effects through apoptosis-mediated tumor cell death in vivo. Scale bar: 10 µm.

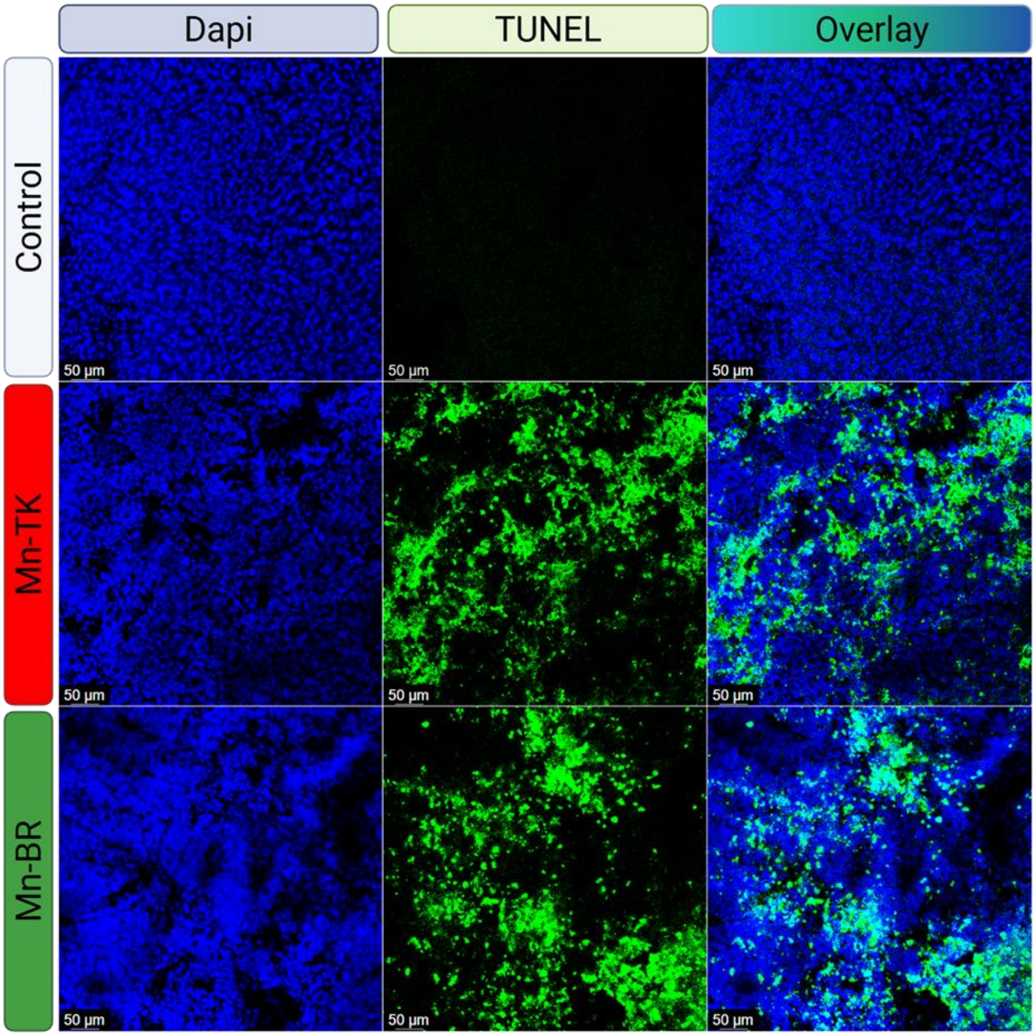

Figure S37. In vivo apoptosis analysis of tumors following Mn-TK and Mn-BR treatment. TUNEL staining of tumor cryosections collected after 14 days of treatment showing apoptotic DNA fragmentation in vivo. DAPI (blue) marks cell nuclei, TUNEL (red) indicates apoptotic nuclei, and merged images show colocalization of TUNEL signal with nuclear staining. Control tumors exhibit minimal TUNEL signal, indicating low basal apoptosis. In contrast, Mn-TK–treated tumors show a substantial increase in TUNEL-positive nuclei distributed throughout the tumor tissue. Mn-BR–treated tumors display the strongest apoptotic response, with extensive regions of TUNEL positivity, confirming robust apoptosis induction. These results demonstrate that Mn-TK and Mn-BR exert their antitumor effects through apoptosis-mediated tumor cell death in vivo. Scale bar: 50 µm

8. Enhancing MRI-Guided Chemotherapy for Brain Tumors with Metal-Organic Structures Mn-TK and Mn-BR

8.1. Spontaneous Glioblastoma (GBM) Mouse Model

In our study, we employed a spontaneous glioblastoma (GBM) mouse model that mirrors the classical subtype of human GBM via expression of heparin-binding epidermal growth factor-like growth factor (HBEGF), a ligand for the epidermal growth factor receptor (EGFR), and Cre-recombinase, which deleted floxed regions of the Ink4a/Arf and Pten genes, in Nestin-expressing cells in the brain as previously described by Shin et al.[28]

On day 28 following tumor induction, mice underwent MRI scans enhanced with gadolinium (Gd) (Multihance) to confirm tumor presence. Only animals with clearly detectable tumors at this time point were included in subsequent analyses. Four days later (day 32), the mice received intravenous injections of their assigned contrast agents. Animals were randomly divided into two groups: the Mn-TK group (n = 3), which received a 0.05 mmol-Mn/kg dose of the Mn-TK agent, and the Mn-BR group (n = 3), which received an equivalent dose of Mn-BR. Four hours post-injection, follow-up MRI scans were conducted to evaluate the in vivo distribution and imaging performance of the respective contrast agents.

8.2. MRI Imaging

In vivo MRI imaging was conducted under isoflurane anesthesia using a Bruker Biospec USR 70/30 7T MRI system (Bruker Biospin, Billerica, USA) equipped with a quadrature surface-only receive head coil and a transmit-only 72 mm diameter volume coil. A T_1-weighted multi spin multi echo (MSME) sequence was used for imaging, with the following parameters: TR of 700 ms, TE of 11 ms, signal averages 1, field of view (FOV) of 20 mm, matrix size of 256 x 256 pixels, and an in-plane resolution of 78 x 78 µm, with a slice thickness of 0.75 mm. During imaging, animal breathing was monitored using SAI Instruments respiration pillow, and heated pads were used to maintain the animals' body temperature. Imaging data were exported to DICOM format and analyzed using Horos software.

8.3. Histological Analysis

Brain tissue from tumor-bearing mice was isolated, fixed in neutral buffered formalin (NBF), and paraffin-embedded. Five µm sections from tissue blocks were adhered to glass slides, stained by H&E, or left unstained for further analysis.

8.4. Immunohistochemistry (IHC)

Tissue sections were deparaffinized as previously described.[29] IHC was performed with the Ki67 antibody at a 1:100 dilution. Specific details regarding the staining procedures are available upon request. Slides were imaged on a Zeiss Axio microscope equipped with a 5-megapixel camera.

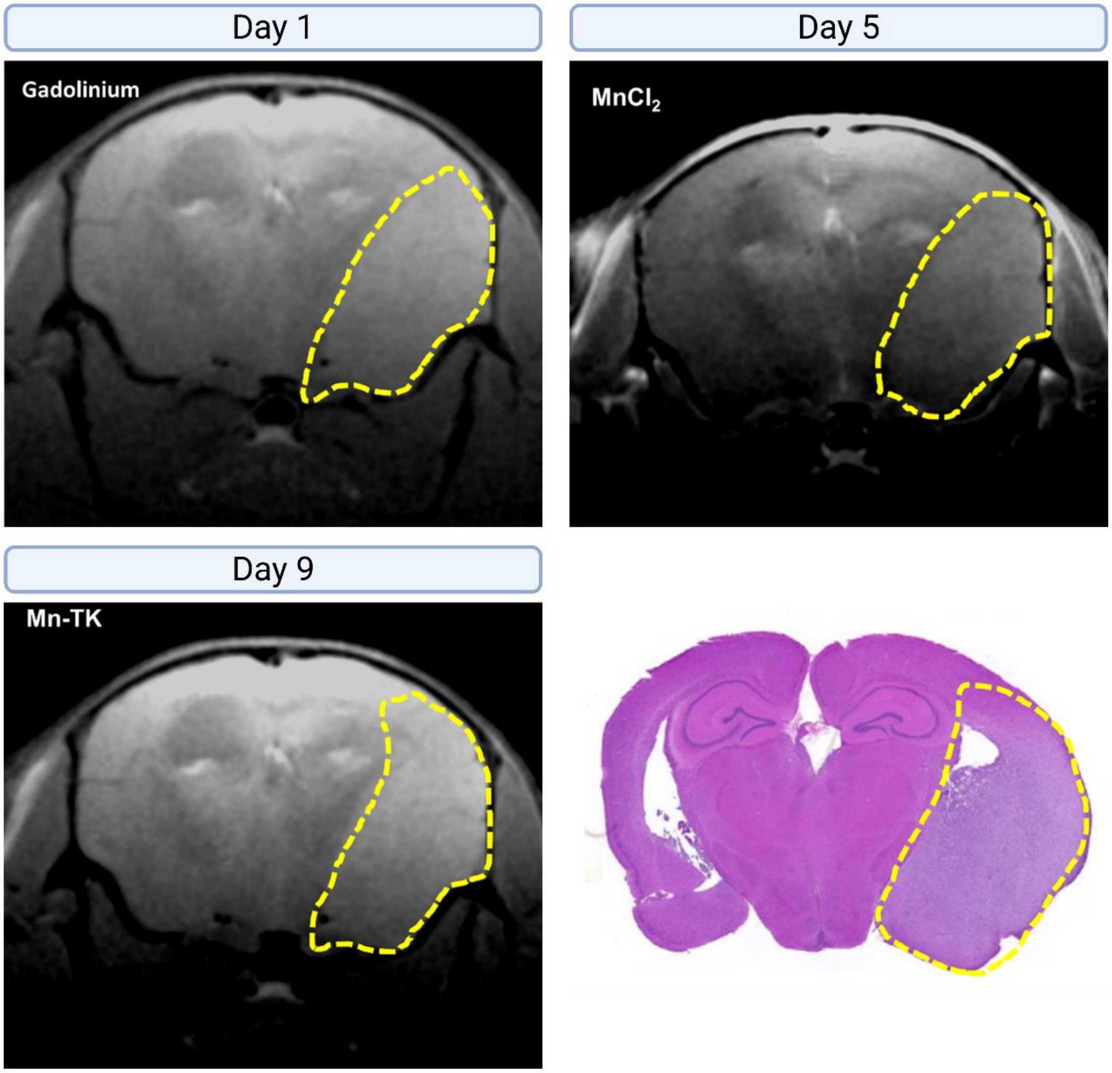

Figure S38. Within-subject MRI comparison of contrast agent performance in a spontaneous GBM model. Coronal T_1-weighted MR images acquired 4 h after a single administration of each contrast agent in the same mouse (4-day washouts between sessions). Gadolinium (Multihance) provided faint and heterogeneous tumor enhancement, indicating rapid clearance and poor retention. $MnCl_2$ produced diffuse, non-selective brain hyperintensity with low tumor-to-brain contrast. In contrast, Mn-TK generated a strong, sharply defined T_1 hyperintensity confined to the tumor with minimal background signal. The enhanced region precisely coincides with the tumor boundary observed in the corresponding H&E section (yellow dashed line), confirming selective intra-tumoral retention and clear delineation of tumor margins.

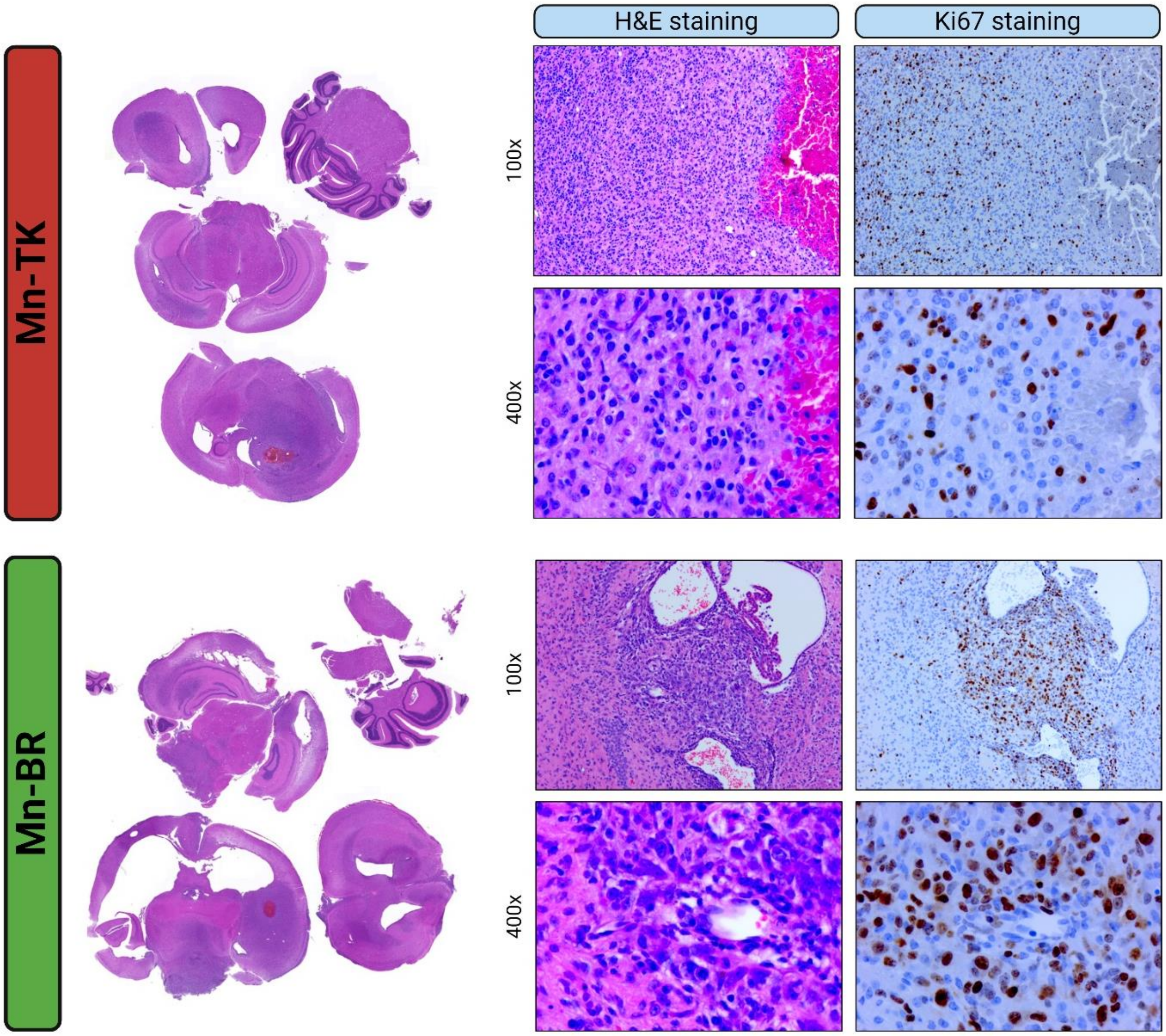

Figure S39. Immunohistochemical staining of tumors. Representative brain sections of mice treated with Mn-TK (top panel) and Mn-BR (bottom panel) (0.05 mmol Mn/kg body weight, 100

µL/mice) and sacrificed (immediately after the scans) were subjected to hematoxylin and eosin (H&E) staining and assessed for proliferation by immunohistochemistry (IHC) for Ki67 (100 and 400X magnification).

8.5. Transwell Permeability Assay

The ability of the Mn-based structures to cross an endothelial monolayer mimicking the blood–brain barrier (BBB) was evaluated using a Transwell transport assay. Mouse brain endothelial bEnd.3 cells (ATCC CRL-2299) were cultured in Dulbecco's Modified Eagle Medium (DMEM) supplemented with 10% fetal bovine serum (FBS) and 1% penicillin–streptomycin at 37 °C under 5% CO_2. For permeability studies, cells were seeded at a density of 1.0×10^6 cells per insert onto collagen-coated polyester Transwell membranes (pore size 0.4 µm, 12-well format, Corning) and allowed to form confluent monolayers for 4–5 days. The integrity of the cell layer was confirmed visually and by measuring trans-endothelial electrical resistance (TEER) using an EVOM² epithelial voltohmmeter, ensuring values above 150 $\Omega \cdot cm^2$ prior to the experiment.

On the day of the assay, cell culture medium in both the apical (upper) and basolateral (lower) chambers was replaced with pre-warmed Hanks' Balanced Salt Solution (HBSS, Ca^{2+}/Mg^{2+}) containing 10 mM HEPES (pH 7.4). Mn-TK and Mn-BR were added to the apical chamber at a final Mn concentration of 10 µM in 0.5 mL, while the basolateral chamber contained 1.5 mL of blank HBSS/HEPES buffer. Plates were incubated at 37 °C for 4 h.

At each time point, 200 µL aliquots were collected from the basolateral compartment and replaced with fresh buffer to maintain constant volume. Samples were acidified with trace-metal grade nitric acid and analyzed for manganese content by inductively coupled plasma mass spectrometry (ICP-MS, Agilent 7800 series). The apparent permeability (P_{app}) was calculated from the cumulative amount of manganese detected in the basolateral chamber as a function of time and membrane surface area.

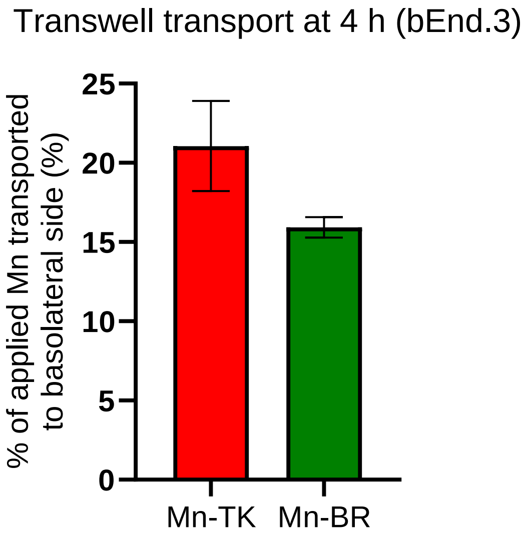

Figure S40. Transendothelial transport of Mn complexes across bEnd.3 monolayers. Quantification of manganese transport through confluent bEnd.3 brain endothelial monolayers grown on Transwell inserts. Cells were incubated with 10 μM of each Mn complex for 4 h in HBSS (± Ca^{2+}/Mg^{2+}, 10 mM HEPES). The percentage of Mn successfully crossing from the apical (luminal) to the basolateral (abluminal) compartment was determined by ICP-MS analysis of Mn content in the receiver chamber. Data represent mean ± SD from n = 3 independent experiments performed on monolayers with TEER > 150 Ω·cm².

9. Statistical Analysis.

All statistical analysis was performed with GraphPad PRISM 8. All data are expressed as mean ±SD. Data were analyzed using one-way ANOVA with post hoc Tukey tests SPSS (IBM, SPSS Statistics, version 23, USA). ** p <0.05; ** p <0.01; *** p <0.001.*

References

1. Gottlieb, H. E.; Kotlyar, V.; Nudelman, A., NMR chemical shifts of common laboratory solvents as trace impurities. *J. Org. Chem.* **1997,** *62* (21), 7512-7515.

2. Fulmer, G. R.; Miller, A. J.; Sherden, N. H.; Gottlieb, H. E.; Nudelman, A.; Stoltz, B. M.; Bercaw, J. E.; Goldberg, K. I., NMR chemical shifts of trace impurities: common laboratory solvents, organics, and gases in deuterated solvents relevant to the organometallic chemist. *Organometallics* **2010,** *29* (9), 2176-2179.

3. Meyer, C. D.; Forgan, R. S.; Chichak, K. S.; Peters, A. J.; Tangchaivang, N.; Cave, G. W. V.; Khan, S. I.; Cantrill, S. J.; Stoddart, J. F., The Dynamic Chemistry of Molecular Borromean Rings and Solomon Knots. *Chemistry – A European Journal* **2010,** *16* (42), 12570-12581.

4. Wang, J.; Wang, H.; Ramsay, I. A.; Erstad, D. J.; Fuchs, B. C.; Tanabe, K. K.; Caravan, P.; Gale, E. M., Manganese-Based Contrast Agents for Magnetic Resonance Imaging of Liver Tumors: Structure-Activity Relationships and Lead Candidate Evaluation. *J Med Chem* **2018,** *61* (19), 8811-8824.

5. Islam, M. K.; Kim, S.; Kim, H. K.; Park, S.; Lee, G. H.; Kang, H. J.; Jung, J. C.; Park, J. S.; Kim, T. J.; Chang, Y., Manganese Complex of Ethylenediaminetetraacetic Acid (EDTA)-Benzothiazole Aniline (BTA) Conjugate as a Potential Liver-Targeting MRI Contrast Agent. *J Med Chem* **2017,** *60* (7), 2993-3001.

6. Mallik, R.; Saha, M.; Sarmah, A.; Singh, V.; Mohan, H.; Bhat, P.; Kumaran, S. S.; Mukherjee, C., A Bis(Aquated) Mn(II)-Based MRI Contrast Agent with a Rigid Hydroquinazoline Unit: Synthesis, Characterization, and in Vivo MR Imaging Study. *ACS Applied Bio Materials* **2024,** *7* (3), 1831-1841.

7. Islam, M. K.; Baek, A.-R.; Yang, B.-W.; Kim, S.; Hwang, D. W.; Nam, S.-W.; Lee, G.-H.; Chang, Y., Manganese (II) Complex of 1,4,7-Triazacyclononane-1,4,7-Triacetic Acid (NOTA) as a Hepatobiliary MRI Contrast Agent. *Pharmaceuticals* **2023,** *16* (4), 602.

8. Bertin, A.; Steibel, J.; Michou-Gallani, A.-I.; Gallani, J.-L.; Felder-Flesch, D., Development of a Dendritic Manganese-Enhanced Magnetic Resonance Imaging (MEMRI) Contrast Agent: Synthesis, Toxicity (in Vitro) and Relaxivity (in Vitro, in Vivo) Studies. *Bioconjugate Chemistry* **2009,** *20* (4), 760-767.

9. Gale, E. M.; Zhu, J.; Caravan, P., Direct Measurement of the Mn(II) Hydration State in Metal Complexes and Metalloproteins through 17O NMR Line Widths. *Journal of the American Chemical Society* **2013,** *135* (49), 18600-18608.
10. Findeisen, M.; Brand, T.; Berger, S., A 1H-NMR thermometer suitable for cryoprobes. *Magnetic Resonance in Chemistry* **2007,** *45* (2), 175-178.
11. Zech, S. G.; Eldredge, H. B.; Lowe, M. P.; Caravan, P., Protein binding to lanthanide(III) complexes can reduce the water exchange rate at the lanthanide. *Inorg Chem* **2007,** *46* (9), 3576-84.
12. Nofiele, J. T.; Cheng, H.-L. M., Ultrashort Echo Time for Improved Positive-Contrast Manganese-Enhanced MRI of Cancer. *PloS one* **2013,** *8* (3), e58617.
13. Rohrer, M.; Bauer, H.; Mintorovitch, J.; Requardt, M.; Weinmann, H. J., Comparison of magnetic properties of MRI contrast media solutions at different magnetic field strengths. *Investigative radiology* **2005,** *40* (11), 715-24.
14. Islam, M. K.; Kim, S.; Kim, H.-K.; Park, S.; Lee, G.-H.; Kang, H. J.; Jung, J.-C.; Park, J.-S.; Kim, T.-J.; Chang, Y., Manganese Complex of Ethylenediaminetetraacetic Acid (EDTA)–Benzothiazole Aniline (BTA) Conjugate as a Potential Liver-Targeting MRI Contrast Agent. *Journal of Medicinal Chemistry* **2017,** *60* (7), 2993-3001.
15. Botta, M.; Carniato, F.; Esteban-Gómez, D.; Platas-Iglesias, C.; Tei, L., Mn(II) Compounds as an Alternative to Gd-Based MRI Probes. *Future Medicinal Chemistry* **2019,** *11* (12), 1461-1483.
16. Islam, M. K.; Baek, A. R.; Yang, B. W.; Kim, S.; Hwang, D. W.; Nam, S. W.; Lee, G. H.; Chang, Y., Manganese (II) Complex of 1,4,7-Triazacyclononane-1,4,7-Triacetic Acid (NOTA) as a Hepatobiliary MRI Contrast Agent. *Pharmaceuticals (Basel, Switzerland)* **2023,** *16* (4).
17. Henoumont, C.; Devreux, M.; Laurent, S., Mn-Based MRI Contrast Agents: An Overview. *Molecules* **2023,** *28* (21).
18. Zhu, J.; Gale, E. M.; Atanasova, I.; Rietz, T. A.; Caravan, P., Hexameric MnII Dendrimer as MRI Contrast Agent. *Chemistry – A European Journal* **2014,** *20* (44), 14507-14513.
19. Nagendraraj, T.; Kumaran, S. S.; Mayilmurugan, R., Mn(II) complexes of phenylenediamine based macrocyclic ligands as T1-MRI contrast agents. *Journal of Inorganic Biochemistry* **2022,** *228*, 111684.

20. Geraldes, C. F. G. C.; Castro, M. M. C. A.; Peters, J. A., Mn(III) porphyrins as potential MRI contrast agents for diagnosis and MRI-guided therapy. *Coordination Chemistry Reviews* **2021,** *445*, 214069.
21. Huang, C. C.; Khu, N. H.; Yeh, C. S., The characteristics of sub 10 nm manganese oxide T1 contrast agents of different nanostructured morphologies. *Biomaterials* **2010,** *31* (14), 4073-8.
22. Taylor, K. M.; Rieter, W. J.; Lin, W., Manganese-based nanoscale metal-organic frameworks for magnetic resonance imaging. *J Am Chem Soc* **2008,** *130* (44), 14358-9.
23. Forgács, A.; Regueiro-Figueroa, M.; Barriada, J. L.; Esteban-Gómez, D.; de Blas, A.; Rodríguez-Blas, T.; Botta, M.; Platas-Iglesias, C., Mono-, bi-, and trinuclear bis-hydrated Mn(2+) complexes as potential MRI contrast agents. *Inorg Chem* **2015,** *54* (19), 9576-87.
24. Dobrovolskaia, M. A.; Aggarwal, P.; Hall, J. B.; McNeil, S. E., Preclinical Studies To Understand Nanoparticle Interaction with the Immune System and Its Potential Effects on Nanoparticle Biodistribution. *Mol. Pharm.* **2008,** *5* (4), 487-495.
25. Dobrovolskaia, M. A.; McNeil, S. E., Understanding the correlation between in vitro and in vivo immunotoxicity tests for nanomedicines. *J. Control. Release* **2013,** *172* (2), 456-466.
26. Morera, D.; MacKenzie, S. A., Is there a direct role for erythrocytes in the immune response? *Vet. Res.* **2011,** *42* (1), 89-89.
27. Macías-Martínez, B. I.; Cortés-Hernández, D. A.; Zugasti-Cruz, A.; Cruz-Ortíz, B. R.; Múzquiz-Ramos, E. M., Heating ability and hemolysis test of magnetite nanoparticles obtained by a simple co-precipitation method. *J. Appl. Res. Technol.* **2016,** *14* (4), 239-244.
28. Shin, C. H.; Robinson, J. P.; Sonnen, J. A.; Welker, A. E.; Yu, D. X.; VanBrocklin, M. W.; Holmen, S. L., HBEGF promotes gliomagenesis in the context of Ink4a/Arf and Pten loss. *Oncogene* **2017,** *36* (32), 4610-4618.
29. Philip, B.; Yu, D. X.; Silvis, M. R.; Shin, C. H.; Robinson, J. P.; Robinson, G. L.; Welker, A. E.; Angel, S. N.; Tripp, S. R.; Sonnen, J. A.; VanBrocklin, M. W.; Gibbons, R. J.; Looper, R. E.; Colman, H.; Holmen, S. L., Mutant IDH1 Promotes Glioma Formation In Vivo. *Cell reports* **2018,** *23* (5), 1553-1564.
